# Tumor evolution metrics predict recurrence beyond 10 years in locally advanced prostate cancer

**DOI:** 10.1038/s43018-024-00787-0

**Published:** 2024-07-12

**Authors:** Javier Fernandez-Mateos, George D. Cresswell, Nicholas Trahearn, Katharine Webb, Chirine Sakr, Andrea Lampis, Christine Stuttle, Catherine M. Corbishley, Vasilis Stavrinides, Luis Zapata, Inmaculada Spiteri, Timon Heide, Lewis Gallagher, Chela James, Daniele Ramazzotti, Annie Gao, Zsofia Kote-Jarai, Ahmet Acar, Lesley Truelove, Paula Proszek, Julia Murray, Alison Reid, Anna Wilkins, Michael Hubank, Ros Eeles, David Dearnaley, Andrea Sottoriva

**Affiliations:** 1 Evolutionary Genomics and Modelling Lab, Centre for Evolution and Cancer, The Institute of Cancer Research, London, UK; 2 St. Anna Children’s Cancer Research Institute, Vienna, Austria; 3 The Royal Marsden NHS Foundation Trust, London, UK; 4 Oncogenetics Team, The Institute of Cancer Research, London, UK; 5 Division of Radiotherapy and Imaging, The Institute of Cancer Research, London, UK; 6 St. George’s Hospital Healthcare NHS Trust, London, UK; 7 Division of Surgery and Interventional Science, UCL, London, UK; 8 Computational Biology Research Centre, Human Technopole, Milan, Italy; 9 Molecular Pathology Section, The Institute of Cancer Research, London, UK; 10 Clinical Genomics, The Royal Marsden NHS Foundation, London, UK; 11 University of Milano Bicocca, Milan, Italy; 12 Bob Champion Cancer Unit, The Institute of Cancer Research and Royal Marsden NHS Foundation Trust, London, UK; 13 Department of Biological Sciences, Middle East Technical University, Ankara, Turkey; 14 Academic Urology Unit, The Royal Marsden NHS Foundation Trust, London, UK

**Keywords:** Prostate cancer, Phylogenetics, Prognostic markers, Tumour heterogeneity, Cancer

## Abstract

Cancer evolution lays the groundwork for predictive oncology. Testing evolutionary metrics requires quantitative measurements in controlled clinical trials. We mapped genomic intratumor heterogeneity in locally advanced prostate cancer using 642 samples from 114 individuals enrolled in clinical trials with a 12-year median follow-up. We concomitantly assessed morphological heterogeneity using deep learning in 1,923 histological sections from 250 individuals. Genetic and morphological (Gleason) diversity were independent predictors of recurrence (hazard ratio (HR) = 3.12 and 95% confidence interval (95% CI) = 1.34–7.3; HR = 2.24 and 95% CI = 1.28–3.92). Combined, they identified a group with half the median time to recurrence. Spatial segregation of clones was also an independent marker of recurrence (HR = 2.3 and 95% CI = 1.11–4.8). We identified copy number changes associated with Gleason grade and found that chromosome 6p loss correlated with reduced immune infiltration. Matched profiling of relapse, decades after diagnosis, confirmed that genomic instability is a driving force in prostate cancer progression. This study shows that combining genomics with artificial intelligence-aided histopathology leads to the identification of clinical biomarkers of evolution.

## Abstract (web summary)

Sottoriva and colleagues combine next-generation sequencing and AI-aided histopathology to assess tumor evolvability in patient samples with long-term follow-up and find that it can be a strong predictor of recurrence in high-risk prostate cancer.

## Main

A substantial proportion of localized and locally advanced prostate cancers can be cured with radiotherapy, usually in combination with androgen deprivation therapy (ADT) or radical prostatectomy. Nevertheless, a substantial group of individuals will experience recurrence. Distinguishing potentially lethal cancers that need additional treatment from those that only need localized treatment is currently a clinical challenge. Risk stratification is an important unmet clinical need in prostate cancer, and novel predictive and prognostic biomarkers are needed. Predicting relapse is difficult, and current clinical standards for risk stratification, such as Gleason score, International Society of Urological Pathology (ISUP) risk group classification^1,2^, prostate-specific antigen (PSA) levels, clinical risk classifier algorithms^3,4^ or even genomic signatures^5,6^, are inadequate to determine the preferred treatment for individuals. Moreover, the extensive heterogeneity of prostate cancer, both between^7^ and within individuals^8–10^, makes genomic data hard to interpret in a clinically meaningful way. Clonal evolution is the fundamental paradigm used to make sense of tumor biology^11^, and, therefore, evolutionary metrics are hypothesized to be powerful predictors of future tumor progression, as demonstrated in the progression of Barrett’s esophagus to esophageal cancer^12,13^. However, there is a general lack of studies measuring spatial intratumor genetic and phenotypic heterogeneity in clinically annotated cohorts of individuals with long-term follow-up information that would enable the predictive power of new evolutionary biomarkers to be tested.

In prostate cancer, seminal studies have evaluated genomic measurements^14–16^, sometimes in combination with microenvironmental^17^ and proteomic measurements^10^, as prognostic biomarkers. However, those studies were limited to single samples per individual and focused on early disease, and most were not performed within a clinical trial setting. Although many biomarkers work reasonably well for very early disease, for locally advanced cancers, prognostication is particularly challenging. Moreover, previous investigations mostly considered individuals that underwent radical prostatectomy^14,18^, representing only one clinical subgroup of individuals with low-to-intermediate risk. Importantly, treatment decisions need to be made using diagnostic biopsies rather than postoperative tissue, as was the case in previous studies. Here, we link spatial genetic variation, measured by next-generation sequencing, with morphological variation, measured with artificial intelligence (AI)-aided computational histopathology, to assess the power of applying evolutionary measures to predict long-term recurrence in high-risk and locally advanced prostate cancer.

## Results

### Study design

**Fig. 1:**
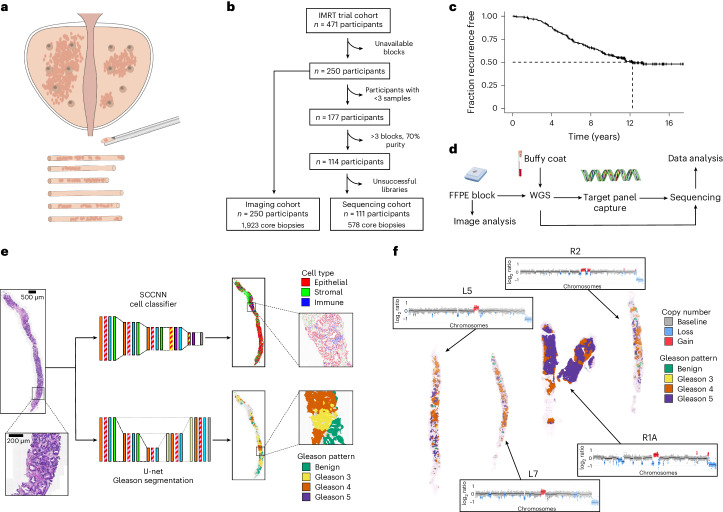
Study design. **a**, Within the IMRT clinical trial (NCT00946543), 6–12 ultrasound-guided diagnostic needle biopsies were taken per individual for routine diagnosis and were embedded in paraffin. **b**, Decision tree for the imaging cohort (*n* = 250 individuals, *n* = 1,923 biopsies) and sequencing cohort (*n* = 111 individuals, *n* = 578 biopsies). The DELINEATE trial cohort was not included. **c**, Kaplan–Meier curve for time to recurrence in the imaging cohort (*n* = 250 individuals). **d**, Experimental workflow for FFPE biopsies and matched germline (buffy coat/normal FFPE tissue). Figure created with BioRender.com. **e**, Computational histopathology analysis with deep learning both for Gleason segmentation and single-cell classification on H&E sections. Sample input and output is shown for FI-115-S8; SCCNN, spatially constrained convolutional neural network. **f**, Example of individual FI-132, where computational Gleason segmentation and CNA genomic data were compared.

The IMRT clinical trial (NCT00946543) recruited 471 individuals who received neoadjuvant/adjuvant ADT and intensity-modulated radiotherapy to the prostate and pelvic lymph nodes, as per the trial guidelines^19^, which represents the current standard of care for radiation treatment of prostate cancer^20–22^. Enrolled individuals were considered high or very high risk according to National Comprehensive Cancer Network guidelines, with a previously reported recurrence rate of 40%. Six to 12 multiregion, spatially separated formalin-fixed paraffin-embedded (FFPE) needle biopsies were available per participant (Fig. 1a). Independent of the original clinical assessment, pathology was also reviewed on a core-by-core basis by a single specialist uropathologist (C.M.C.).

From the IMRT clinical cohort, we selected individuals with available tissue blocks and an associated hematoxylin and eosin (H&E)-stained section (*n* = 250). From this set, we selected individuals with at least three biopsies containing cancer tissue at sufficient purity (70% cancer) for sequencing (*n* = 114). Genomic library preparation failed in 3 individuals, yielding a final set of 111 individuals with available sequencing data (Fig. 1b). To assess the extent of spatial tumor variation, we also selected three individuals from a subgroup of the DELINEATE clinical trial (ISRCTN04483921), where 48 needle biopsies were available per case^23,24^. All biopsies had been prospectively collected and reviewed by a specialist uropathologist (C.M.C.), and informed written consent was given by all participants. Full clinical data are available for this cohort, including participant and tumor characteristics, treatments received and prostate cancer outcomes and survival, with a median of 12.5 years of follow-up following radiotherapy (Fig. 1c). Recurrence included biochemical-only recurrence, local recurrence or metastatic recurrence of prostate cancer. A biochemical-only recurrence was defined as a PSA level of >2 ng ml^–1^ above the nadir PSA after radiotherapy in the absence of disease seen on imaging. Time to recurrence was defined as the time from the completion date of radiotherapy to first recurrence. A summary of the clinical characteristics of the IMRT trial cohorts is reported in Supplementary Table 1.

We performed low-pass whole-genome sequencing (WGS) in 642 tumor samples (median per participant = 5) from the 114 participants (111 IMRT and 3 DELINEATE), henceforth referred to as the sequencing cohort. The whole set of somatic copy number calls is available in Supplementary Table 2. We also performed deep targeted sequencing of a prostate cancer gene panel (Supplementary Table 3) with unique molecular identifiers (UMIs) in 588 tumor samples (median per participant = 5, median coverage after UMI compression: 141×). The whole set of somatic calls for single-nucleotide variants (SNVs) and small deletions and insertions is available in Supplementary Table 4. For 100 participants, we had available fresh-frozen buffy coat samples from the UK Genetic Prostate Cancer Study (UKGPCS; NCT01737242) trial and performed WGS with a median coverage of 36× (Fig. 1d). We also performed computational histopathology analysis with deep learning on 1,923 H&E sections from all 250 participants with available tissue blocks (Fig. 1e). All IMRT participants and samples included in the sequencing cohort were also a part of this imaging cohort. The resulting dataset provided matched intratumor genetic and morphological heterogeneity, both in terms of Gleason grade and cellular composition, for a large set of participants in the IMRT trial (Fig. 1f and Supplementary Table 1).

As part of the study, those involved in sample preparation and data analysis were blinded until completion of the primary phase of data analysis. As a result, the selection of samples for sequencing and imaging and the selection and computation of genomic and histological metrics were finalized before unblinding. Review pathology, including Gleason grade, was also undertaken blinded to the original pathology and clinical data. To further explore associations that were identified in the primary phase, a secondary phase of data analysis was conducted after unblinding, which was focused on newly generated cell-free DNA (cfDNA) and multiplex immunohistochemistry data.

### The landscape of spatial genetic variation

**Fig. 2:**
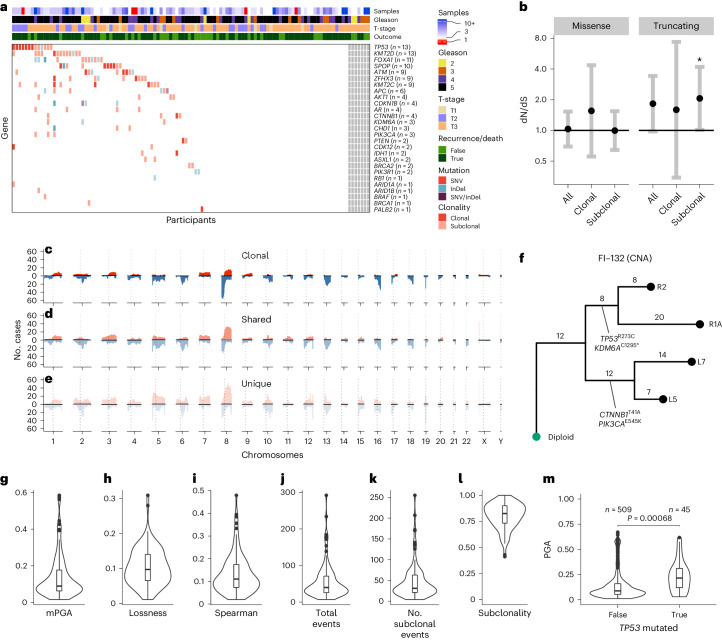
Genetic intratumor heterogeneity landscape of locally advanced prostate cancer. **a**, Heat map representing the mutational landscape of the cohort (*n* = 114 participants), including number of low-pass WGS samples with detected CNAs, ISUP grade group (reviewing pathologist for IMRT participants, original specialist uropathologist S. Hazell (Royal Marsden NHS Foundation Trust) for DELINEATE, where participants were not reviewed), T-stage and recurrence/death status. Mutations are colored and shaded by their type (SNV and insertion/deletion (InDel)) and clonality status (clonal/subclonal). **b**, dN/dS analysis of all mutations using dNdScv for missense and truncating mutations shows subclonal truncating mutations to be under positive selection. Clonal and subclonal mutations were taken only from participants with three or more targeted sequencing samples (all *n* = 107 participants, clonal/subclonal *n* = 98 participants). Intervals represent 95% CI, and the centers represent the maximum likelihood estimate. **c**–**e**, CNA landscape of prostate cancer defined by phylogenetic status per case. Gains (red) and losses (blue) are represented relative to ploidy of the samples (*n* = 111 participants). **f**, An example of MEDICC2-inferred CNA phylogeny in FI-132 with manually annotated driver SNVs. **g**–**l**, Genomic metrics of genomic instability and heterogeneity were calculated before outcome unblinding (*n* = 109 participants, sequencing cohort participants with three or more samples with a PGA of ≥0.01). **m**, *TP53*-mutant samples presented with significantly higher PGA (linear mixed effects model, two-sided *t*-test on gradient, s.e. = 0.02, d.f. = 552, *t* = 3.4, samples with a PGA of ≥0.01, *n* = 554 samples). Box plots show the center line as the median and box limits as upper and lower quartiles. Whiskers extend no further than 1.5× the interquartile range past the box limits, and points represent outliers. Source data

**Extended Data Fig. 1:**
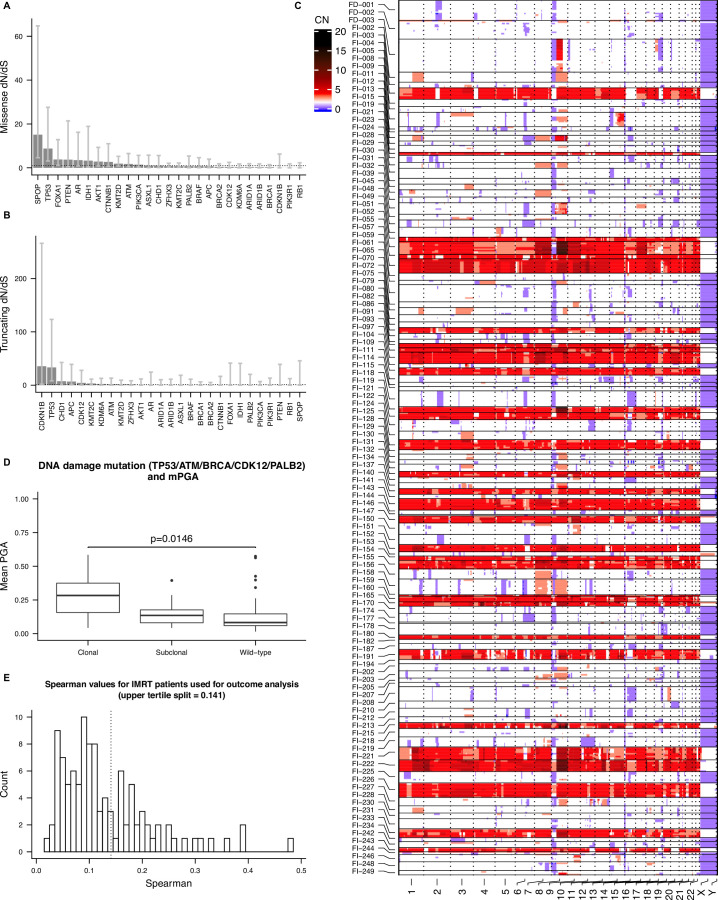
Genomic analysis of locally-advanced prostate cancer. (**A**) Estimates of per-gene dN/dS for missense mutations only. dN/dS maximum likelihood estimates are calculated per gene by dNdScv. Intervals represent 95% confidence level, the bars represent the maximum likelihood estimate and dotted line represents dNdS **=** 1 (neutral). Missense mutations are positively selected in *SPOP*, *TP53* and *FOXA1* as the lower bounds of the intervals are greater than 1. (**B**) Estimates of per-gene dN/dS for truncating mutations only. Intervals represent 95% confidence level, the bars represent the maximum likelihood estimate and dotted line represents dNdS **=** 1 (neutral). Truncating mutations are positively selected in *CDKN1B* and *TP53*. Number of mutation types per gene are provided in source material. (**C**) A heatmap of per sample absolute copy number calls. Chromosomes are represented on the x-axis and samples are grouped by participant on the y-axis, separated by lines (n **=** 609 samples, n **=** 114 participants). Copy numbers are not normalised relative to baseline ploidy. (**D**) DNA damage mutations versus mean PGA. Boxplots represent mean PGA (mPGA) separated based on the clonality of mutations involved in DNA damage response (*TP53*, *ATM*, *BRCA1/2*, *CDK12*, *PALB2*). The status is considered clonal if any of the mutations are detected in all samples. mPGA is significantly higher in participants with a clonal DNA damage mutation compared to participants with no DNA damage mutations (2-sided t-test, standard error **=** 0.055, d.f. **=** 10.1, t **=** 2.94). Mutations are split into clonal (n **=** 10 participants), subclonal (n **=** 13 participants) and wild-type (n **=** 75 participants). Boxplots show centre line as median, box limits as upper and lower quartiles, whiskers extend no further than 1.5x interquartile range past the box limits and points represent outliers. (**E**) Distribution of Spearman values for cohort (n **=** 106 participants) used for outcome analysis displays a long tail of high values that are discriminated by a threshold of the upper tertile (dotted line). Source data

In our analysis of the sequencing cohort samples with successful targeted sequencing, we found mutations in a putative prostate cancer driver gene in at least one sample in 61/107 participants, with many mutations detected being subclonal (79.4% of mutations in participants with three or more biopsies; Fig. 2a). Indeed, the ratio of non-synonymous to synonymous substitutions (dN/dS) analysis indicated that subclonal truncating mutations were under significant positive selection (dN/dS = 2.06, 95% confidence interval (95% CI) = 1.01–4.19; Fig. 2b). Given the extensive multifocality and polyclonal origin of prostate cancer previously reported^25^, many of these subclonal mutations may represent different independent tumors rather than subclonal expansions within an established single malignancy. The most common clonal mutations were found in *TP53* (*n* = 6) and *SPOP* (*n* = 5), and these genes were also the only genes found to be under significant positive selection across all substitution types (*TP53:* *q* = 2.42 × 10^−5^; *SPOP*: *q* = 7.46 × 10^−4^; Extended Data Fig. 1a,b). *CDKN1B* and *TP53* were under positive selection when considering only truncating mutations (*CDKN1B*: dN/dS = 35.4, 95% CI = 1.8–266; *TP53*: dN/dS = 33.3, 95% CI = 8.3–123; Extended Data Fig. 1b). When analyzed together with copy number profiles, split between clonal, shared (intermediate phylogenetic tree branches) and unique (tip branches) copy number alterations (CNAs; Fig. 2c–e and Extended Data Fig. 1c), the genomic patterns confirm the likely multifocality of the disease. Recurrent focal amplifications were found in *MDM2* (*n* = 4), *MYCN* (*n* = 4), *FGFR1* (*n* = 3) and *MYC* (*n* = 3). Of note, *MDM2* and *MYCN* were amplified together in three individuals. We could reconstruct the phylogenetic tree based on CNAs in 111 individuals, including the 3 DELINEATE participants (Fig. 2f and Supplementary Note Fig. 1; see Methods). We then calculated multiple heterogeneity and genomic instability metrics, including mean proportion genome altered (mPGA; Fig. 2g), patterns of lossness of small copy number fragments (Fig. 2h), heterogeneity measurement of mean Spearman correlation (1 – Spearman’s *ρ*, referred to as ‘Spearman’ for the rest of the manuscript) between the log_2_ ratios (raw copy number signal) of all pairwise comparisons of samples within a participant (Fig. 2i and Methods) and the total number of phylogenetic CNA events (Fig. 2j), subclonal events (Fig. 2k) and their proportions (Fig. 2l). These metrics were computed before unblinding of outcome data. *TP53* mutations were associated with higher burden of chromosomal alterations (0.069 greater PGA in *TP53*-mutant samples, *P* = 0.0007 linear mixed effects model; Fig. 2m). Furthermore, clonal DNA damage gene mutations were also associated with higher mPGA (Extended Data Fig. 1d).

### Spatial genetic divergence predicts time to recurrence

**Fig. 3:**
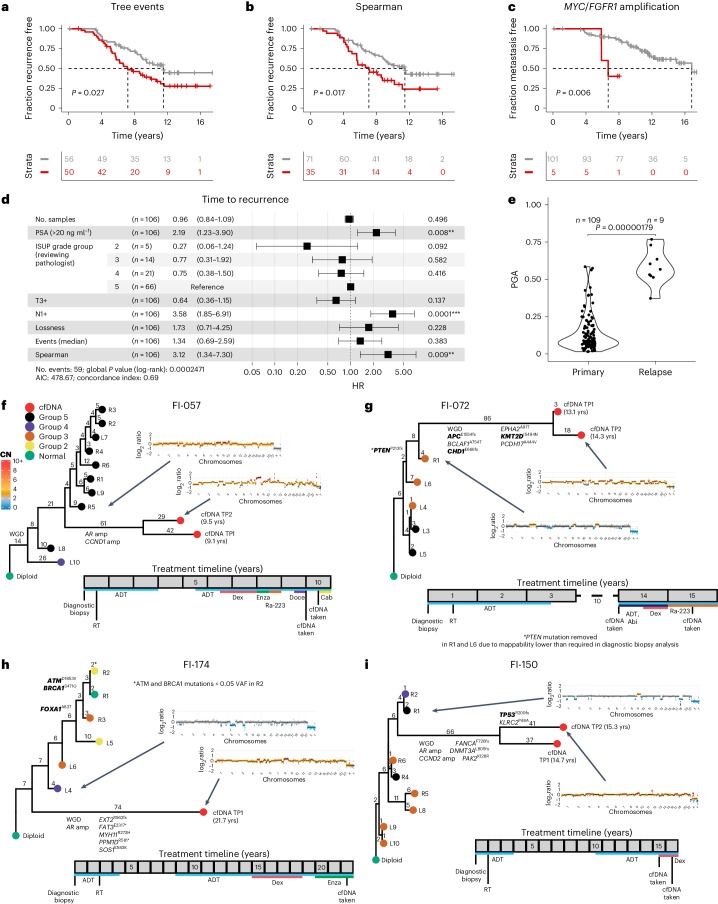
Spatial genetic diversity and phylogenetic events predict recurrence. **a**,**b**, Total phylogenetic tree events (two-sided log-rank test, *χ*^2^ = 4.9, d.f. = 1; **a**) and the Spearman metric (two-sided log-rank test, *χ*^2^ = 5.7, d.f. = 1; **b**) predict earlier time to recurrence (*n* = 106 participants, sequencing cohort IMRT participants with three or more samples with a PGA of ≥0.01). **c**, Amplification in *MYC* and/or *FGFR1* (coamplified in one participant) predicts earlier time to metastasis (two-sided log-rank test, *χ*^2^ = 7.5, d.f. = 1, *n* = 106 participants). **d**, Cox proportional hazards (CPH) model of time to recurrence using clinical covariates and number of low-pass WGS samples with CNAs. Three metrics significant in a univariate CPH model (*P* < 0.1) are also included in the model (natural log of lossness, total phylogenetic events split by median value and Spearman). The forest plot shows 95% CI of HRs and the covariate *P* values, derived from a Wald test (*n* = 106 participants, *P < 0.05, **P < 0.01, ***P < 0.001). HRs for lossness and Spearman represent the increase in hazard between their 5th and 95th percentile values (within the sequencing cohort). **e**, mPGA per participant in primary samples (*n* = 109 participants) compared to the mPGA of individual relapse samples (*n* = 9 samples, two-sided Mann–Whitney *U-*test, *W* = 962). **f**–**i**, Phylogenetic analysis of primary and relapse samples (cfDNA) taken at recurrence. Tips of tumor nodes represent either the automated classifier ISUP grade group (primary diagnostic biopsies) or a cfDNA sample (red). Time since the diagnostic biopsy is labeled next to the cfDNA nodes in years (yrs). Representative copy number profiles are shown for a single cfDNA sample and the primary diagnostic biopsy that is most related to the cfDNA. Edges are labeled with phylogenetic events plus specific CNA events (for example, whole-genome duplication (WGD) or gene amplification (amp)) or detected point mutations. Genes present in the diagnostic biopsy panel are highlighted in bold and may be detected in both the primary and relapse samples. Genes not in bold are only detectable in the relapse samples and may also be present in the diagnostic biopsies. Below each tree, the timeline shows treatment history. Each event is rounded to the nearest 6 months. Each square represents a year. Treatment descriptions are written in shorthand; Abi, abiraterone acetate; Cab, cabazitaxel; CN, copy number; Dex, dexamethasone; Doce, docetaxel; Enza, enzalutamide; Ra-223, radium-223; RT, radiotherapy; Salv. HiFU, salvage high-intensity focused ultrasound; VAF, variant allele frequency. Source data

At the onset of this study, we hypothesized that evolutionary patterns measurable only through multiregion sequencing, such as intratumor heterogeneity, would predict clinical outcome. Within the sequencing cohort, we found that the number of CNA events in the phylogenetic tree predicted shorter time to recurrence in the univariate analysis when split using the median value (*P* = 0.027 log-rank test, median time to recurrence 7.2 and 11.5 years; Fig. 3a). Additionally, the upper tertile of the Spearman heterogeneity index also predicted a shorter time to recurrence (*P* = 0.017 log-rank test, median time to recurrence 7.1 and 11.5 years; Fig. 3b and Methods). The upper tertile threshold captured the long tail of high Spearman values in the cohort (Extended Data Fig. 1e). We also found a small subgroup of participants (*n* = 5) with focal amplifications in either *MYC* or *FGFR1* that showed particularly poor prognosis for time to metastasis specifically (*P* = 0.006 log-rank test, median time to metastasis 6.7 for *MYC* or *FGFR1* amplified versus 16.8 years for no amplification; Fig. 3c).

**Extended Data Fig. 2:**
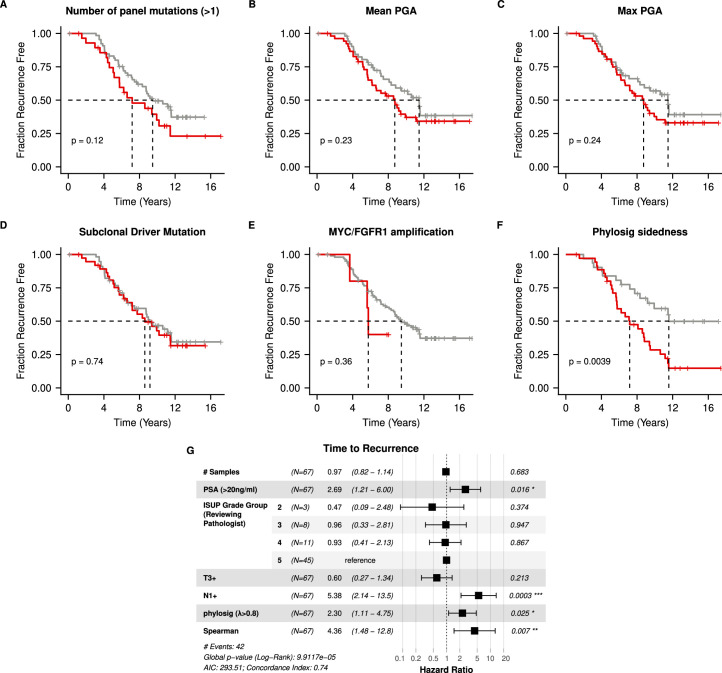
Time to recurrence analysis of genomic metrics. (**A**) Participants are split between those with >1 mutated gene on the panel (red, n = 29 participants) and those with 1 or 0 (grey, n = 66 participants). This threshold produced the best split of the data in time to recurrence analysis. (**B**) Participants are split by the median mPGA. Participants with high mPGA (red, n = 53 participants) do not have significantly shorter time to recurrence than those with low mPGA (grey, n = 53 participants). (**C**) Participants are split by the median max PGA. Participants with high max PGA (red, n = 53 participants) do not have significantly shorter time to recurrence than those with low max PGA (grey, n = 53 participants). (**D**) Participants are split between those that had a subclonal mutation on the driver gene panel (red, n = 38 participants) and those without (grey, n = 57 participants). *KMT2C* and *KMT2D* were excluded from this analysis. (**E**) Participant with an amplification in *MYC* and/or *FGFR1* (red, n = 5 participants) did not have significantly different time to recurrence than those with an absence of either amplification (grey, n = 101 participants). All p values are calculated using a log-rank test. (**F**) Participants with strong sidedness (greater clustering of right and left regions across the MEDICC2 tree, lambda > 0.8, red, n = 36 participants) show a significantly shorter time to recurrence (log-rank test) than the remaining participants for which phylogenetic signal analysis was possible (grey, n = 31 participants). (**G**) Cox proportional hazards (CPH) model of time to recurrence using clinical co-variates, phylosig sidedness classification and Spearman (n = 67 participants). Forest plot shows 95% confidence interval of hazard ratios, and the covariate *P* values, derived from a Wald test (*P < 0.05, ** *P* < 0.01, ***P < 0.001). Hazard ratio for Spearman represents the increase in hazard between the 5th and 95th percentile values (within the Sequencing Cohort). Source data

**Extended Data Fig. 3:**
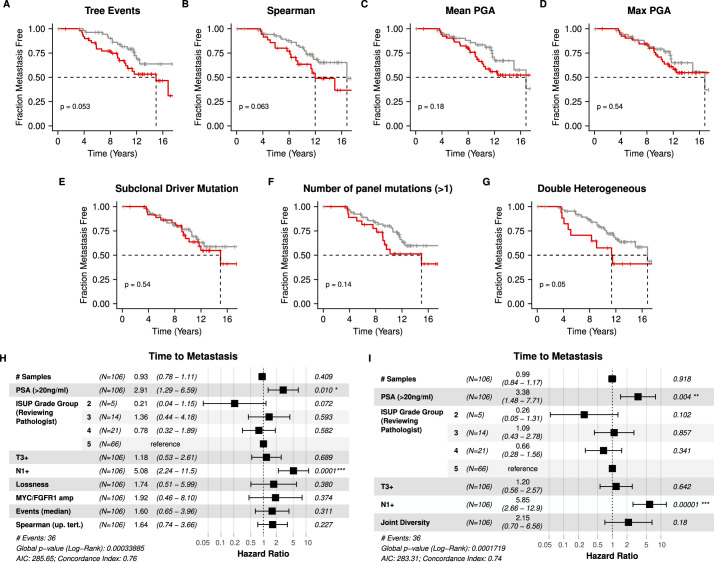
Time to metastasis analysis of genomic metrics. (**A**) Participants with a high number of events (greater than median, red, n = 50 participants) did not have a significantly shorter time to metastasis than those with fewer events (grey, n = 56 participants). (**B**) Participants split by the upper tertile of the Spearman metric (red, n = 35 participants) and the lower two tertiles (grey, n = 71 participants). (**C**) Participants are split by the median mPGA. Participants with high mPGA (red, n = 53 participants) do not have significantly shorter time to metastasis than those with low mPGA (grey, n = 53 participants). (**D**) Participants are split by the median max PGA. Participants with high max PGA (red, n = 53 participants) do not have significantly shorter time to metastasis than those with low max PGA (grey, n = 53 participants). (**E**) Participants are split between those that had a subclonal mutation on the driver gene panel (red, n = 38 participants) and those without (grey, n = 57 participants). *KMT2C* and *KMT2D* were excluded from this analysis. (**F**) Participants are split between those with >1 mutated gene on the panel (red, n = 29 participants) and those with 1 or 0 (grey, n = 66 participants). (**G**) Participants split equally to Fig. 5G. Double heterogeneous participants (red, n = 17 participants) had a significantly shorter time to metastasis than the rest (grey, n = 89 participants, *P* = 0.0497). P values are calculated using a log-rank test. (**H**) CPH model, using clinical co-variates and genomic metrics with p < 0.1 in a univariate CPH model, for time to metastasis. (**I**) Cox proportional hazards (CPH) model, using clinical co-variates and Joint Diversity metric, for time to metastasis. Forest plots show 95% confidence interval of hazard ratios, and the covariate *P* values, derived from a Wald test (*P < 0.05, **P < 0.01, ***P < 0.001). Hazard ratios for Lossness and Joint Diversity represent the increase in hazard between their 5th and 95th percentile values (within the Sequencing Cohort). Source data

Most importantly, multivariate analysis for time to recurrence confirmed that the Spearman heterogeneity measure was a powerful independent prognostic factor, with a hazard ratio (HR) of 3.12 (95% CI = 1.34–7.3, *P* = 0.009), providing additional prognostic power to N(nodal)-stage greater than N0 at diagnosis, which showed an HR of 3.58 (95% CI = 1.85–6.9, *P* < 0.001) and PSA of >20 ng ml^–1^, with an HR of 2.19 (95% CI = 1.23–3.9, *P* = 0.008; Fig. 3d and Supplementary Table 5). Genomic burden, either expressed by number of mutations in driver genes (Extended Data Fig. 2a) or PGA (mean or maximum), which has been previously reported as associated with survival^17^, was not prognostic in our cohort (Extended Data Fig. 2b,c). Participants with subclonal driver mutations did not have significantly worse time to recurrence (Extended Data Fig. 2d). We report the univariate KM curves for time to metastasis in Extended Data Fig. 3.

We next sought to investigate the relationship between phylogenetic history and location within the prostate. For 108 participants in the IMRT cohort for which phylogenetic trees were available, there were 68 participants with sufficient data to assess the clustering of left and right regions (see Methods). Thirty-seven participants (54.4%) showed strong clustering of the left and right regions (*λ* > 0.8), and this was significant for 14 participants (*P* < 0.05). Alternatively, 26 participants (38.2%) showed mixing of left and right sides (*λ* < 0.2). Participants showing a strong phylogenetic clustering of the right and left sides had shorter time to recurrence (Extended Data Fig. 2f; *P* = 0.039 log-rank test, *n* = 36 versus 31, median 7.2 versus 11.6 years until recurrence; 1 participant did not overlap with the 106 participants used for outcome analysis). Phylogenetic signal sidedness was also robust to multivariate analysis (Extended Data Fig. 2g).

### Genomic instability is enhanced at relapse

**Extended Data Fig. 4:**
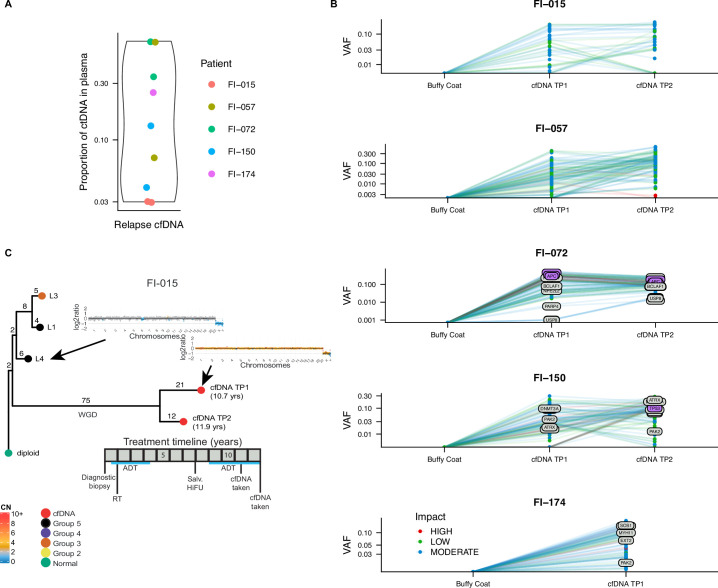
Time series genomic analysis of ctDNA samples at recurrence. (**A**) Distribution of the proportion of ctDNA in plasma across 9 cfDNA samples in 5 participants within fragments between 90 and 150 bp in size. (**B**) Trajectories of changes in variant allele frequency (VAF) across cfDNA timepoints for 5 participants. Lines and dots represent synonymous and non-synonymous mutations derived from whole exome sequencing and their impact as determined by VEP. Buffy coats represent control samples. Mutations are labelled if they belong to the targeted panel used for the primary samples (purple) or if they overlap with the IntOGen (www.intogen.org) pan-cancer driver list (grey) (n = 35 mutations, n = 100 mutations, n = 111 mutations, n = 94 mutations, n = 84 mutations, top to bottom). (**C**) MEDICC2 copy number alteration phylogenetic tree for FI-015 with cfDNA and primary diagnostic biopsy samples. Tips of nodes represent either the ISUP Grade group (primary diagnostic biopsies) or if the sample is a cfDNA sample (red). Time since the diagnostic biopsy is labelled next to the cfDNA nodes in years (yrs). Representative copy number profiles are shown for a single cfDNA sample and the primary diagnostic biopsy that is most related to the cfDNA. Edges are labelled with copy number alteration events (for example, WGD, whole genome duplication). Below the tree the timeline shows treatment history. Each event is rounded to the nearest 6 months. Each square represents a year. Treatment descriptions are written in shorthand (ADT = Androgen Deprivation Therapy, RT = Radiotherapy, Salv. HiFU = Salvage High-intensity Focused Ultrasound). Source data

We tracked participants in the IMRT trial who returned to the clinic with progressive disease many years later. Due to feasibility and risks involved in tissue biopsies for metastatic disease, we focused on collecting plasma for circulating tumor DNA (ctDNA) analysis. We collected ten plasma samples at relapse from five participants taken, on average, 12.9 years (8.1–21.7 range) after the diagnostic biopsies. We used low-pass WGS and whole-exome sequencing combined with UMIs to achieve high sensitivity for low-frequency mutations, with a median coverage of 645× after UMI collapse and a base error rate as low as 0.01%. One sample was excluded from the analysis due to a lack of detectable ctDNA. We inferred the copy number profile of the samples, enhancing the tumor purity with in silico fragment size selection and achieving a reasonable set of tumor purities (Extended Data Fig. 4a). We detected a large number of mutations at relapse (Extended Data Fig. 4b), including one found in the primary tumor with panel sequencing. We found a significantly increased burden of CNAs at relapse, with high PGA levels (Fig. 3e), supporting the finding that chromosomal instability is a driving force of tumor progression in prostate cancer. All four of five participants who had diagnostic biopsies with ploidy of approximately two presented at relapse with whole-genome duplication. We then used the copy number profiles to add the recurrent sample to the phylogenetic trees calculated from the primary multiregion biopsies (Fig. 3f–i and Extended Data Fig. 4c). The recurrent sample originated from distinct locations in the tree in different participants, corroborating the predictive value and biological importance of divergence as a rate of chromosomal instability (that is, a dynamic measure of an evolutionary mutation rate) compared to a static measurement of the most altered clones (for example, mean or maximum PGA at diagnosis), which were not prognostic in this study.

### Spatial morphological heterogeneity predicts recurrence

**Fig. 4:**
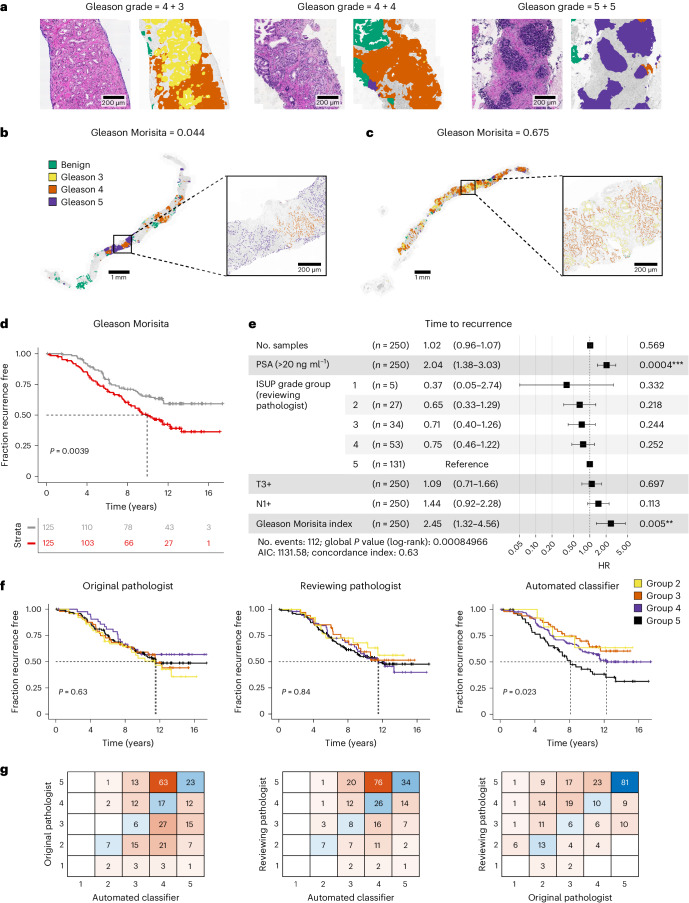
Morphological spatial heterogeneity with deep learning-based Gleason grading. **a**, Example output from the automated Gleason classifier, with primary and secondary pattern assessment. **b**,**c**, Examples of the Gleason Morisita assessment. Cells identified as epithelial cells by the cell classifier are subdivided into Gleason grades using the region’s automated Gleason segmentation. Regions with high segregation of patterns (**b**) will be assigned a low Gleason Morisita index, whereas regions with high mixing between Gleason grades (**c**) will be assigned a high Gleason Morisita index. **d**, Participants with greater within-section heterogeneity of Gleason pattern, as assessed by Gleason Morisita index, are associated with a shorter time to recurrence (two-sided log-rank test, *χ*^2^ = 8.33, d.f. = 1, *P* = 0.0039; imaging cohort, *n* = 250 participants). **e**, CPH model of time to recurrence using clinical covariates and the Gleason Morisita index (imaging cohort, *n* = 250 participants, *P < 0.05, **P < 0.01, ***P < 0.001). The forest plot shows 95% CI of HRs and the covariate *P* values, derived from a Wald test. HR for the Gleason Morisita index represents the increase in hazard between the 5th and 95th percentile values (within the imaging cohort). **f**, ISUP grade group as a predictor of time to recurrence. A comparison is shown for the grade groups assessed by the original reporting pathologist, the reviewing pathologist for the trial and the automated classifier (imaging cohort, *n* = 250 participants). Grade groups are calculated from the assessed primary and secondary patterns, according to the 2014 ISUP criteria. Only the automated Gleason assessment was able to stratify the participants by time to recurrence (two-sided log-rank test, *χ*^2^ = 9.52, d.f. = 3, *P* = 0.023). **g**, Confusion matrices showing the pairwise agreement of the ISUP grade groups reported by the original reporting pathologist, the reviewing pathologist for the trial and the automated classifier (imaging cohort, *n* = 250 participants). Source data

**Extended Data Fig. 5:**
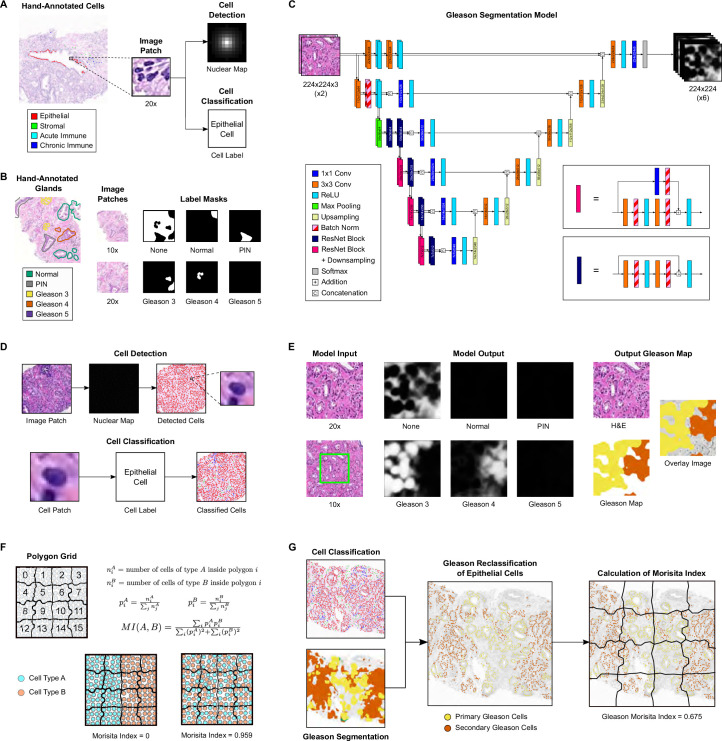
Digital Pathology methods outline. (**A**) Cell detection and classification model training. Cells are manually annotated onto the slide. For cell detection, annotated cells are converted into an input image patch and an output nuclear probability map. For cell classification, annotated cells are converted into an input image patch and a cell label. (**B**) Gleason segmentation model training. Glandular regions are manually annotated onto the slide, which are converted into input images patches and output segmentation masks. Input images are at 20x and 10x magnification, each centred at the same point. Output masks represent the segmentation of each class on the 20x input image. (**C**) Neural network architecture for Gleason segmentation model. Layers are represented by rectangles, colour coded by their type. Arrows indicate connections between layers. Dimensions of the output neurons are listed for each layer. (**D**) Outline of cell detection and classification. From an input image patch, the cell detector outputs a cell probability map, which is converted into cell detections. For each detected nuclei, an image patch is extracted. This is processed by the cell classifier, producing a label for the cell. (**E**) Outline of Gleason segmentation. As input, the network receives 20x and 10x images (20x image region is indicated on the 10x image in green). The network outputs probability maps for each label, corresponding to the 20x image. An output Gleason map is generated from the probability maps. (**F**) Outline of cellular Morisita index. Metric estimates the degree of mixing between two cell populations. Visual examples are shown for both high segregation and high mixing of cells. (**G**) Calculation of Gleason Morisita index. Epithelial cells are reclassified by the Gleason grade of their associated gland. The Gleason Morisita index is computed between the epithelial cells of the section’s primary and secondary Gleason patterns. For further details see ‘Computational Analysis’ supplementary note.

**Extended Data Fig. 6:**
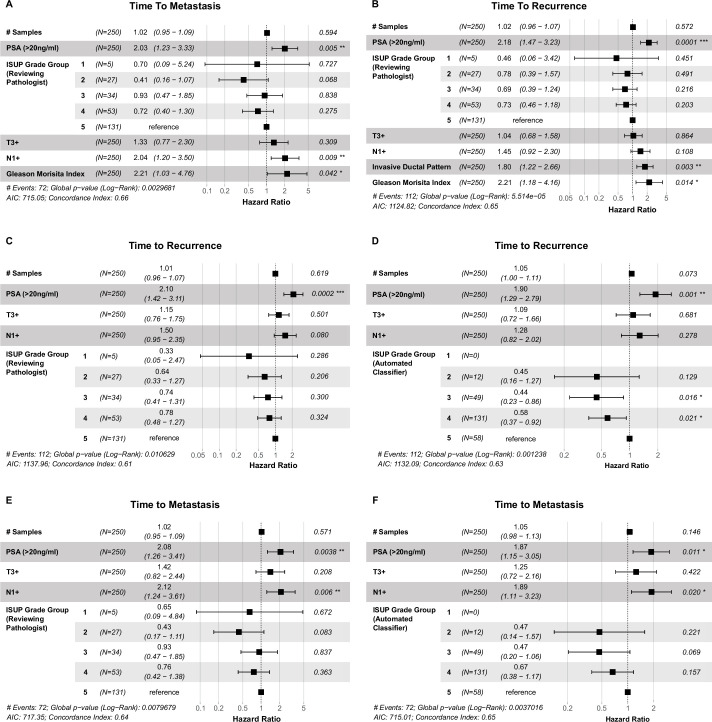
Supplementary Cox proportional hazards (CPH) models from Digital Pathology analysis. (**A**) Time to metastasis using clinical co-variates and Gleason Morisita index (Imaging Cohort, n = 250 participants). Increased Gleason Morisita index is significant associated with shorter time to recurrence (p < 0.05), in line with what is seen in time to recurrence (Fig. 4E). Hazard ratio for Gleason Morisita index represents the increase in hazard between the 5th and 95th percentile values (within the Imaging Cohort). (**B**) Time to recurrence using clinical co-variates, presence of Invasive Ductal Pattern, and Gleason Morisita index (Imaging Cohort, n = 250 participants). Both Gleason Morisita index and Invasive Ductal Pattern are independently significant predictors of risk of patient recurrence (p < 0.05). Presence of Invasive Ductal Pattern was identified at a per-patient level by Reviewing Pathologist. Hazard ratio for Gleason Morisita index represents the increase in hazard between the 5th and 95th percentile values (within the Imaging Cohort). (**C**) Time to recurrence, including clinical covariates and Reviewing Pathologist’s grade grouping (Imaging Cohort, n = 250 participants). Model hazard ratios suggest a decreasing risk of recurrence as grade group decreases, with respect to reference group 5, albeit without significance. (**D**) Time to recurrence, including clinical covariates and grade grouping from automated classifier (Imaging Cohort, n = 250 participants). Grade groups 3 and 4 show significantly lower risk of recurrence (*P* = 0.0164 and *P* = 0.0214, respectively) compared to reference group 5. (**E**) Time to metastasis, including clinical covariates and Reviewing Pathologist’s grade grouping (Imaging Cohort, n = 250 participants). There is no clear trend for grade group. (**F**) Cox model of time to metastasis, including clinical covariates and grade grouping from automated classifier (Imaging Cohort, n = 250 participants). Model hazard ratios suggest a decreasing risk of metastasis from groups 3–5, albeit without significance. All forest plots show 95% confidence interval of hazard ratios and the covariate *P* values, derived from a Wald test (*P < 0.05, **P < 0.01, ***P < 0.001). Source data

Using our automated Gleason classifier (Methods and Extended Data Fig. 5b,c,e), we called gland-level Gleason grade in 1,923 sections from the 250 IMRT trial participants in the imaging cohort. Heterogeneous Gleason grade was widespread, with regions dominated by Gleason patterns 3, 4 and 5 all being observed within the cohort (Fig. 4a). Concomitantly, we used our cell classifier (Methods and Extended Data Fig. 5a,d) to determine if each cell in each biopsy was an epithelial, stromal or immune cell. Leveraging on our ability to automatically assign Gleason grade to all regions of each biopsy, a task that would be extremely difficult to do manually in such a large cohort, we also assessed heterogeneity of tissue morphology in terms of variation in Gleason pattern within a biopsy. We measured spatial heterogeneity of Gleason pattern with the Morisita index^26^ (see Extended Data Fig. 5f,g for details). Low ‘Gleason Morisita’ (defined as <0.30, the median value in the imaging cohort; *n* = 250), identified biopsies with segregated Gleason patterns (Fig. 4b), whereas high scores highlighted biopsies with high intermixing of different Gleason grades in the same patch (Fig. 4c). We found that Gleason Morisita was indeed significantly prognostic (Fig. 4d; *P* = 0.0039) and robust to multivariate analysis with Cox regression (Fig. 4e and Supplementary Table 6; *P* = 0.0046). Gleason Morisita had an HR of 2.45 (95% CI = 1.32–4.56) versus an HR of 2.04 (95% CI = 1.38–3.03) for the best conventional marker for this cohort, which is a PSA of >20 at diagnosis. Gleason Morisita was also significant in the multivariate analysis when considering time to metastasis as an endpoint (Extended Data Fig. 6a; HR = 2.21, 95% CI = 1.03–4.76, *P* = 0.042). We next wanted to investigate whether the Gleason Morisita was just a surrogate of some aggressive subpathology in prostate cancer. We found no significant association between the prevalence of Gleason pattern 5 and Gleason Morisita (*P* = 0.83; data were analyzed by one-way analysis of variance). Interestingly, we found that the invasive ductal pattern was significantly prognostic (HR = 1.8, 95% CI = 1.22–2.66, *P* = 0.003) but was independent of the Gleason Morisita, which remained significant (Extended Data Fig. 6b).

**Extended Data Fig. 7:**
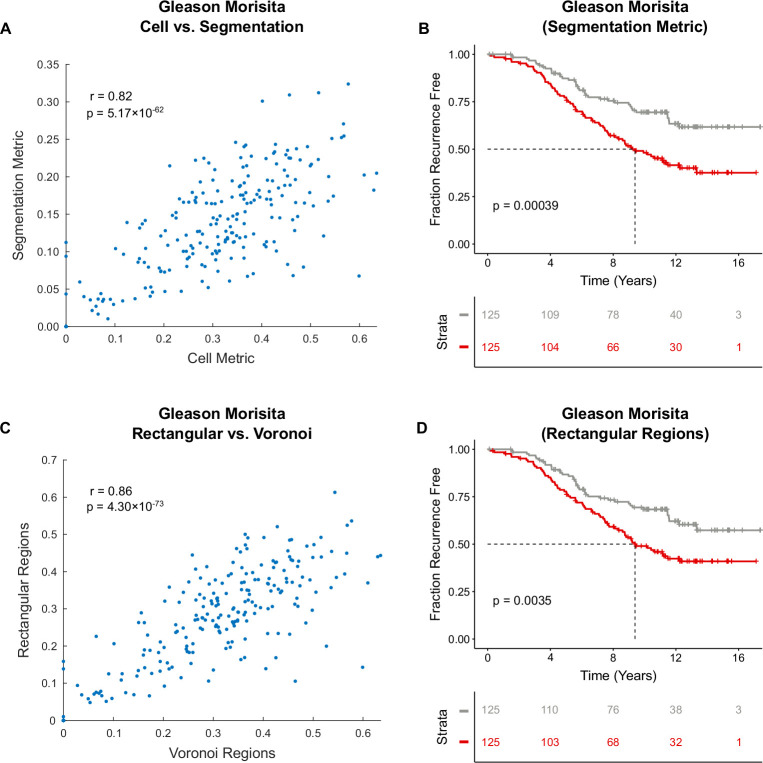
Evaluation of the robustness of Gleason Morisita index. To evaluate the robustness of Gleason Morisita index, two comparisons are made: balance of epithelial cells vs. balance of segmented pixels (**A**, **B**), and Voronoi sub-regions vs. rectangular subregions (C, D). (A) Scatter plot comparing patient-level Gleason Morisita indices from the cell and segmentation-based metrics. The two metrics are well correlated, with a Pearson correlation of 0.82 (Imaging Cohort, n = 250 participants, d.f. = 248, *P* = 5.17 × 10^−62^). (**B**) KM curve of time to recurrence for segmentation-based Gleason Morisita index, split by median. Segmentation-based metric is also a significant predictor of time to recurrence (Imaging Cohort, n = 250 participants, two-sided log-rank test, χ^2^ = 10.43, d.f. = 1, *P* = 0.00039), with the pattern of survival closely resembling that of the cell-based metric (Fig. 4D). (**C**) Scatter plot comparing patient-level Gleason Morisita indices from the Voronoi and rectangular regions. The two metrics are well correlated, with a Pearson correlation of 0.86 (Imaging Cohort, n = 250 participants, d.f. = 248, *P* = 4.3 × 10^−73^). (**D**) KM curve of time to recurrence for Gleason Morisita index from rectangular regions, split by median. Rectangular region metric is also a significant predictor of time to recurrence (Imaging Cohort, n = 250 participants, two-sided log-rank test, χ^2^ = 5.94, d.f. = 1, *P* = 0.0035), with the pattern of survival closely resembling that of the Voronoi region metric (Fig. 4D). Source data

To evaluate the robustness of the Gleason Morisita to differences in computational methodology, we compared the proposed version of the metric to two alternatives (see Methods for details). Both alternative metrics were well correlated with our chosen metric (Extended Data Fig. 7a,c) and demonstrated the same significant prediction of shorter time to recurrence for greater heterogeneity in Gleason pattern (Extended Data Fig. 7b,d), suggesting that the metric is not especially sensitive to the specific methodology.

We then compared our Gleason classifier to two sets of pathologist Gleason scores that were available for the IMRT trial: a per-participant Gleason score performed by the original pathologists, undertaken at the various referring centers between 1997 and 2012 when the participant was first diagnosed, and a core-by-core rescoring by a single specialist uropathologist (C.M.C.), undertaken between 2017 and 2018 using the updated 2014 ISUP criteria^27,28^. This was an opportunity to analyze the degree of interobserver variability of human assessment and change in diagnostic practice over time. To compare pathologists and our deep learning classifier, all assessments were first converted to a grade group (1–5) using the 2014 ISUP criteria^28^. The IMRT trial focuses on high-risk and locally advanced prostate cancers with generally high Gleason score. These may not be adequately stratified by Gleason grade or ISUP grade grouping^29^, which in general have been derived from surgically treated cohorts with less advanced cancers^1^. We confirmed that neither the original scoring of the pathologist nor rescoring by a single expert demonstrated a statistically significant trend in ISUP grade groups. However, automatic Gleason scoring with deep learning showed significant differences in time to recurrence between the different ISUP grade groups (Fig. 4f). In multivariate analysis, although all scorings indicated a pattern of increasing risk for higher grade groups, the reviewing pathologist’s grades did not stratify significantly for recurrence (Extended Data Fig. 6c), whereas the deep learning classifier did (Extended Data Fig. 6d). No scoring remained significant considering time to metastasis as an endpoint (Extended Data Fig. 6e,f). We observed that the degree of disagreement between the reporting and reviewing pathologists was comparable to the deep learning classifier’s disagreement with both the reporting and reviewing pathologists (Fig. 4g). The mean difference in grade groups between the reporting and reviewing pathologists was 0.84, compared to 1.1 between the reporting pathologists and the classifier or 0.92 between the reviewing pathologist and the classifier. Disagreement between original and review pathologists may, in part, be influenced by changes in grading protocol. Although the reviewing pathologist’s assessments were made using the 2014 ISUP criteria^28^, the original pathologist’s assessments all occurred before the 2014 revision, with a subset also predating the 2005 revision^30^. The most common source of disagreement between human and computational assessment was from participants assessed as grade group 5 by the pathologist and group 4 by the classifier. Comparing the review pathologist with the deep learning classifier, it was apparent that results were concordant in 75 cases, upgraded in deep learning in 62 cases but downgraded in 113 cases. This means that many of the highest-grade cancers were subdivided into lower- or higher-risk strata.

### Impact of genomic alterations on cellular morphology

**Fig. 5:**
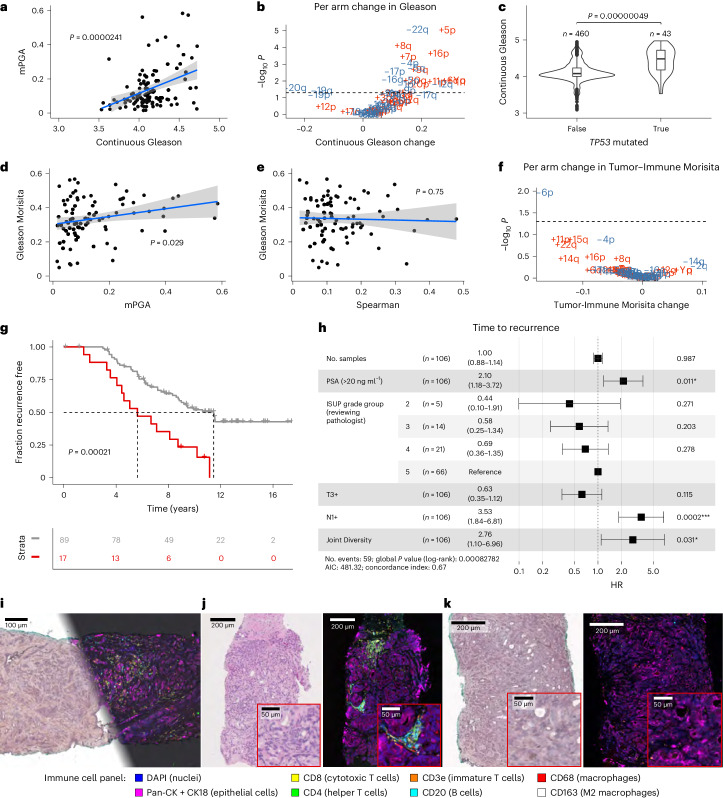
Combining genetic and morphological measurements. **a**, mPGA is associated with higher continuous Gleason (*n* = 106 participants, IMRT participants with three or more samples with a PGA of ≥0.01, linear model, two-sided *t*-test on gradient, estimate = 0.19, s.e. = 0.04, *t* = 4.4, d.f. = 104). Shaded area represents 95% CI in all scatter plots. **b**, Twenty-four chromosome arm changes are associated with a change in continuous Gleason (gains are displayed in red, and losses are displayed in blue; *n* = 62 chromosome arm changes, *P* values were adjusted using the Benjamini–Hochberg method and are derived from two-sided *t*-tests on gradient per arm linear mixed effects model; continuous Gleason change derived from gradient estimate). **c**, The *TP53* mutation is associated with higher continuous Gleason (linear mixed effects model, two-sided *t*-test on gradient, s.e. = 0.06, d.f. = 371, *t* = 5.1, *n* = 503 samples). Box plots show center lines as the median and box limits as upper and lower quartiles. Whiskers extend no further than 1.5× interquartile range past the box limits, and points represent outliers. **d**,**e**, mPGA (linear model, two-sided *t*-test on gradient, estimate = 0.23, s.e. = 0.103, *t* = 2.2, d.f. = 85; **d**), but not Spearman (estimate = −0.05, s.e. = 0.15, *t* = −0.3, d.f. = 85; **e**), is associated with increased mixing of Gleason grades (*n* = 87 participants, sequencing cohort omitting participants with a Gleason Morisita equal to 0, that is, a homogenous Gleason grade). **f**, Chromosome 6p loss is uniquely associated with a reduction in Tumor-Immune Morisita (changes are colored and *P* values were adjusted and derived as in **b**; *n* = 62 chromosome arm changes). Samples in **b**, **c** and **f** have a PGA of ≥0.01. **g**, The most genetically and morphologically heterogeneous tumors are associated with shorter time to recurrence (two-sided log-rank test, *χ*^2^ = 13.7, d.f. = 1, *n* = 106 participants). **h**, The Joint Diversity metric shows significant association with greater risk of recurrence in a CPH model with clinical covariates. The forest plot shows 95% CI of HRs, and the covariate *P* values are derived from a Wald test. The HR for Joint Diversity represents the increase in hazard between the 5th and 95th percentile values (within the sequencing cohort, *n* = 106 participants, *P < 0.05, **P < 0.01, ***P < 0.001). **i**, Multiplex immunohistochemistry and H&E staining was performed on the same section. Immunohistochemistry experiments were run once following optimization and validation. **j**, Example of an immune-hot region on matched H&E (left) and multiplex immunohistochemistry (right) images. **k**, Example of an immune-cold region on matched H&E (left) and multiplex immunohistochemistry (right) images. Source data

In cancer, the genotype–phenotype map that connects DNA information inside the cell with its behavior and morphology is largely missing. Using our sequencing cohort, which contains matched histology and sequencing data for all 111 IMRT participants, we aimed at exploiting this multimodal data to identify associations between variation of genomic features and diversity of phenotypic (morphological) Gleason patterns. We were able to measure Gleason scores by mean grade as a function of area that we term ‘continuous Gleason’ (see Methods), which would be very difficult to achieve at this scale without deep learning image analysis. We found that continuous Gleason significantly correlates with mPGA (Fig. 5a; *P* = 0.000024, linear model) and total phylogenetic events (*P* = 0.0004; Extended Data Fig. 9a). We found 24 chromosome arm-level CNAs to be associated with a change in continuous Gleason (Fig. 5b), of which 22 changes correlated with an increase in Gleason, indicating a set of specific CNAs associated with tumor dedifferentiation. In the subset of chromosome arms displaying a significant association with increased Gleason, gains and losses showed an overrepresentation of oncogenes and tumor suppressors^31^, respectively (Extended Data Fig. 9b). Higher continuous Gleason was also associated with *TP53* mutations (Fig. 5c; *P* = 5 × 10^−7^, linear mixed effects model), further supporting the link of this gene with advanced disease. One chromosome arm alteration associated with increased Gleason was 5p gain, an event that is enriched in metastatic prostate cancers versus primary prostate cancers^32^. A significant correlation was also found between mPGA and mean Gleason Morisita (Fig. 5d; *P* = 0.029, linear model; Methods), indicating that increased copy number burden is also associated with increased Gleason mixing and dedifferentiation. The Spearman metric of genetic heterogeneity did not correlate with Gleason Morisita, suggesting that genetic and morphological diversity capture distinct biology (Fig. 5e; *P* = 0.75, linear model).

Using our deep learning cell-type classifier trained on epithelial, stromal and immune cells, we found high levels of infiltration of inflammatory cells (which can include lymphocytes, macrophages, neutrophils and plasma cells) in a proportion of IMRT cases (Supplementary Table 7), suggesting that at least a subgroup of locally advanced prostate cancer is not completely immune cold, as reported for early prostate cancer^33^. Indeed, we found an association between higher PGA and reduced immune infiltration, as measured by the Tumor-Immune cell Morisita index (Extended Data Fig. 9c). Notably, the only chromosomal arm that was associated with reduced immune infiltration was chromosome 6p, containing the *HLA* locus (Fig. 5f and Extended Data Fig. 9d; *P* = 0.00017, linear mixed effects model, adjusted *P* = 0.011, Benjamini–Hochberg method). Also, when using the percentage of immune cells identified by the cell classifier, we found that 6p loss significantly reduced the number of immune cells in the sample (Extended Data Fig. 9e). These results suggest that chromosomal instability and *HLA* loss of heterozygosity are associated with immune evasion in prostate cancer, as previously reported in lung cancer^34^, ovarian cancer^35^ and melanoma^36^, among others^37^.

Combining the previously used upper tertile of the highest genetic heterogeneity index (Spearman) and the upper half of the morphological heterogeneity (Gleason Morisita), we were able to identify a subgroup of 17/106 (16%) participants with much poorer prognosis (Fig. 5g; *P* = 0.00021, log-rank test). Next, we sought to combine these metrics into a single measurement that we termed ‘Joint Diversity’, calculated as the geometric mean of the Spearman and Gleason Morisita measurements, allowing us to identify the most genetically and morphologically diverse individuals. Joint Diversity was robust to multivariate analysis (Fig. 5h and Supplementary Table 8), with an HR of 2.76 (95% CI = 1.095–7, *P* = 0.031).

### Immune hot spots are detectable by multiplexed immunohistochemistry

To further investigate the role of immune cells in our cohort, we used highly multiplexed immunohistochemistry (Fig. 5i) with 15 markers (Supplementary Table 9). We selected a subset of 20 samples from seven participants within the imaging cohort to include those with both high and low Tumor-Immune Morisita indexes. We found clear hot spots of immune infiltration in a substantial subset of sections (13 samples; Fig. 5j and Extended Data Fig. 10a,b). Other samples were almost devoid of any immune cells (Fig. 5k). Multiplex immunohistochemistry images were then visually inspected, and the abundance of each marker was scored on a scale from 0 to 5 (Supplementary Table 10). We found that the CD20 marker of B cells correlated significantly with the Immune Morisita index (*P* = 0.0065). This suggests that B lymphocytes may be playing a role in immune infiltration in prostate cancer (see other examples in Extended Data Fig. 10c,d). In addition, CD68^+^ cells were abundant in most of our samples; however, CD163^+^ cells were almost completely absent (Extended Data Fig. 10e,f), indicating the presence of M1, but not M2, macrophages. Together, these data suggest that even when tumor-immune infiltration is present, immune cells remain inactive or potentially repressed by tumor cells. Further functional investigation will be needed to unveil the cross-talk between cancer and immune cells in locally advanced prostate cancer.

## Discussion

The lack of powerful prognostic markers in prostate cancer leads to suboptimal treatment stratification. There is a need to identify high-risk nonmetastatic individuals that will benefit from early adjuvant use of new life-prolonging treatments, such as abiraterone acetate^38^. Conversely, although chemotherapy with adjuvant docetaxel used with ADT may not improve overall survival for individuals with localized high-risk prostate cancer^39^, it is possible that biomarkers might identify high-risk subgroups for whom this treatment does produce improved outcomes. It would also be of value to define subpopulations of individuals who could avoid the detriments of long-term systemic treatments but maintain good outcomes. Cancer is a complex disease governed by evolutionary rules^11,40^. Evolution is about ‘change over time’, emphasizing the need to understand the dynamic behavior of tumors^41^ to make future clinical predictions. Although following tumors longitudinally in humans remains difficult, intratumor heterogeneity can be seen as a looking glass into cancer evolution^42^ as it encodes the tumor’s history and can help predict its future. Evolvability is a central feature of cancer and contains information on its future adaptation, for example, in the form of mutation rate. Seminal multiregion studies have radically changed the way we understand human cancers from an evolutionary perspective^43,44^, but multisampling remains laborious, expensive and difficult to apply within a clinical trial. Moreover, most of these studies are still small, involve a few samples per individual and, with the exception of the TRACERx trials in lung^45^ and renal cancer^46^, have not yet been linked to clinical trial information, especially in prostate cancer. Here, we leveraged the ultrasound-guided multiregion biopsy strategy that is standard of care for the diagnosis of prostate cancer to collect data that are amenable to evolutionary studies.

We report that spatial genomic and morphological divergence were significantly associated with recurrence. Thanks to the clinical trial design, we could ensure that these metrics were robust to multivariate analysis. Different from previous investigations, which used single samples per individual and focused on early-stage prostate cancer, we did not find PGA to be prognostic, even when using the same thresholds (from ref. ^17^, threshold = 7.49% and *P* = 0.26; from ref. ^47^, threshold = 5.4% and *P* = 0.057). As a continuous metric, mPGA was also not prognostic in a multivariate analysis (*P* = 0.235). This suggests that measures of heterogeneity and evolvability may be more effective in predicting recurrence than static measurements of burden of alterations in the cancer cell genome. One may note that the Spearman metric measures heterogeneity between samples, whereas Gleason Morisita measures morphological heterogeneity within samples. Given that we observed that it was specifically individuals with both high genetic and high morphological heterogeneity that had significantly worse time to recurrence, it may indicate that diversity must be present both locally and globally across the tumor for risk of recurrence to increase.

Moreover, AI-driven Gleason scoring allowed unprecedented associations between genomic alterations and aberrant cell morphology. We found that increased aneuploidy was linked to both higher Gleason grades and greater local heterogeneity of Gleason pattern. This suggests that progressive alterations of chromosomes may drive dedifferentiation. The association of a plethora of chromosome arm changes with increased Gleason grade suggests a set of chromosomal alterations that are primarily associated with progression and may be positively selected. Interestingly, two chromosome arm losses (−19q and −20q) were associated with reduced Gleason grade, suggesting that there may be chromosomal alterations that block dedifferentiation. Furthermore, copy number burden was also associated with reduced Tumor-Immune Morisita, suggesting a role for genome-wide aneuploidy in immune evasion. However, chromosome arm analysis suggests that only chromosome 6p loss is specifically related to immune evasion in prostate cancer. This correlation between loss of chromosome 6p and directly observed immune evasion in prostate cancer builds on similar findings in other cancer types^34–36,48,49^. Prostate cancer has recently been shown to be one of the few cancer types with an increased frequency of immune evasion alterations in metastatic tumors^50^, indicating that immune evasion may be a key feature of tumor aggressiveness.

It should be highlighted that we used only widely available FFPE diagnostic biopsies and applied low-coverage WGS, which is relatively inexpensive and hence potentially applicable to routine clinical practice. Furthermore, our deep learning classifiers operate on H&E-stained sections, which are standard in routine clinical practice. Thus, our classifiers could be extended to other prostate cancer cohorts once the sections have been scanned and digitized. In this work, we directly compared machine assessment of Gleason grading to multiple assessments from expert pathologists. On central review, ISUP grade groups 2 and 3 had more favorable outcomes than ISUP grade groups 4 and 5, but groups 4 and 5 were not clearly distinguished. There appeared to be little relationship with outcome for the initial local pathological assessment. This may, in part, reflect a change in pathological assessment over time^1^ as well as potential benefit from specialist uropathology review. One notable area of disagreement between the machine classification and human assessment is in the assignment of grade group 5. For both sets of human assessments, group 5 was the most common grade. By contrast, group 4 was the most common in the machine assessment, with most of these individuals being assessed as group 5 by both pathologists. This is, in part, a consequence of the method by which the automated classifier computes a patient-level ISUP grade group from the participant’s individual slide grade groups (see Supplementary Note). For an individual to be classified as group 5 by the automated classifier, all individual sample grade groups must also be group 5, which is likely to differ from the determination made by pathologists. Given that the machine grade grouping produced a better stratification of recurrence, it could be inferred that, although the pathologist’s grading of these participants as group 5 may well have been correct according to current ISUP criteria, the grade group criteria itself may be insufficient to fully determine the risk of recurrence for patients in high-risk groups. Group 5 may benefit from being divided into two categories, allowing the very highest risk individuals to be more clearly identified. We also introduce a measure of the heterogeneity of Gleason patterns within a section, Gleason Morisita. From a biological perspective, frequent intermixing of Gleason patterns may indicate that the tumor is in a transitionary state between the lower and higher grade. What is seen in the biopsy is the state of the tumor at a single time point in the tumor’s evolutionary trajectory. Thus, although standard assessment of Gleason pattern is accurately describing the state of the tumor at this time point, Gleason Morisita may be capturing additional signal of the tumor’s evolutionary trajectory.

In the future, these ‘evolvability’ metrics could be used in conjunction with established clinical variables as well as commercially available transcriptomic tests^51^ to optimally predict recurrence in prostate cancer, particularly for individuals with high-risk or locally advanced disease. However, our findings will first need to be validated in larger cohorts and tested within cohorts with different risk profiles to fully understand how these predictors apply more generally. Regardless, our approach of combined genomic and histological analysis within trial datasets demonstrates an effective strategy for studying tumor evolution within routinely collected clinical samples.

## Methods

### Ethical approval

All research was performed in accordance with local and national ethical standards, and the study protocol was approved by the West of Scotland Research Ethics Service in December 2017 (HRA ID 230542). The research was performed at the Institute of Cancer Research, London.

### Clinical cohort

The IMRT trial (NCT00946543) recruited 471 participants with high-risk or locally advanced prostate cancer between 2000 and 2013. All participants received hormone deprivation and radiotherapy to the prostate and lymph nodes. The median age was 65. The sex of all participants was male (gender information was not collected at the time of study recruitment). Informed consent was obtained for all participants, and no participants were compensated for participation in the study. Further clinical characteristics of these participants were previously described^19^. For each participant, 6–12 18-mm, multiregion ultrasound-guided needle biopsies were taken from the primary site, which were then formalin fixed and paraffin embedded for histopathological analysis.

After a median follow-up of 12.5 years, the recurrence rate was 40%. Clinical data were compiled for each participant, which included TNM staging, Gleason grading, PSA levels, number and location of the core biopsies, age, treatment received and prostate cancer outcome and survival data. All individuals involved in sample preparation and data analysis were blinded to clinical data until the completion of the primary phase of data analysis.

Two hundred and fifty participants had accessible FFPE blocks, for a total of 1,923 biopsies, from which H&E sections were taken and used for image analysis. Eligibility criteria for the sequencing cohort included participants with greater than or equal to three tumor biopsies and at least 70% cancer purity, as assessed by the original pathologist. In total, 111 participants fulfilled those features, adding up to 578 biopsies.

As a comparable cohort, we included three participants from DELINEATE (ISRCTN04483921), an ongoing single prospective phase 2 trial of intermediate- or high-risk prostate adenocarcinoma opened in 2011 (ref. ^23^). This trial is assessing toxicity and feasibility of a radiotherapy boost to tumor nodules within the prostate at the time of primary radiotherapy. Like the IMRT trial, image-guided biopsies were also taken; however, up to 48 mapping template needle biopsies were obtained in a subset of this cohort, collecting a total of 65 tumor biopsies from the three selected participants for this study.

For germline data, 100 buffy coat samples were collected from the UKGPCS trial (NCT01737242) for those individuals where they were available. For seven participants with unavailable buffy coats, normal FFPE needle biopsies were used as a substitute. However, for the remaining seven participants where neither buffy coats nor normal biopsies were available, no germline sample was collected.

For collection of cfDNA samples in participants with recurrent prostate cancer, the clinical study EXCERPT (NCT04686188) was initiated. Participants who experienced a recurrence of prostate cancer and had been treated within the IMRT trial were recruited to donate blood samples if they (1) had not yet commenced treatment for recurrence, (2) had progressive disease on treatment or (3) had a PSA level of >2 ng ml^–1^ on treatment. Up to three blood samples were collected for each participant at different time points. Clinical course information, including dates and types of recurrence and treatments received, was recorded for each participant.

### Sample preparation

Original pathology reports containing Gleason score, biopsy location and tumor purity description were received together with the available blocks from 250 participants. To standardize the pathological assessment, including Gleason grading, which was originally undertaken at a number of different hospitals over many years, a new H&E staining was performed on the first 4-μm section of each block, and all slides were re-evaluated by a central specialist uropathologist (C.M.C.) at The Institute of Cancer Research/Royal Marsden Hospital. A minimum of 70% tumor purity, according to the pathological purity estimates, was used to select blocks that would be eligible for sequencing. To define biopsy location, samples were renamed accordingly by right, left, middle or apex, followed by the number of the biopsy on the original report. Between 15 and 20 10-µm sections were taken from the FFPE needle biopsies according to their width and were collected in a tube. For those with enough material, 2 × 5 µm sections were taken in the middle of the block and stored for future characterization.

Following Quick-DNA FFPE Miniprep (Zymo Research, D3067), DNA was extracted and quantified by Qubit 3.0 fluorometer (Invitrogen, Q33216). Extracted DNA was then incubated at 20 °C for 15 min with NEBNext FFPE DNA Repair Mix (New England Biolabs, M6630) to correct all possible changes due to the formalin fixation process. Subsequently, a clean-up was performed using 2.5× SPRI beads (Beckman Coulter, B23318), and, after two washes with 80% ethanol, repaired DNA was eluted and requantified.

Whole-genome libraries were generated from at least 30 ng of DNA using a low-input NEBNext Ultra II DNA library Prep kit for Illumina (New England Biolabs, E7645) and NEBNext Multiplex Oligos for Illumina (Unique Dual Index UMI Adaptors DNA set 1, New England Biolabs, E7395L), which contains 96 unique dual index adaptors and a UMI sequence to enable the identification and removal of PCR errors or duplicates from amplified libraries. A brief enzymatic fragmentation step of 3 min was performed and, based on the initial yield, between six and nine PCR cycles were used for library enrichment. Elution was done in 38 μl of TE buffer (Invitrogen, 12090015), and quality control was checked by High Sensitivity D1000 ScreenTape (Agilent, 5067-5584) on a 4200 TapeStation System (Agilent, G2991BA) and Qubit 3.0 fluorometer (Invitrogen, Q33216).

After whole-genome library preparation, around 190 ng was used for panel capture following the manufacturer’s instructions. The custom panel was designed to include the most mutated genes, specifically, those that were previously identified in >2% of primary prostate tumors. The panel included the coding regions of the 27 most commonly mutated genes and the promoter noncoding regions of *FOXA1* and *NEAT1*, where mutations were also assessed (Supplementary Table 3). Panel development was done by Twist Bioscience for a final total target region of 375,569 base pairs (bp), which was directly covered by 3,396 probes. Eight indexed whole-genome libraries were pooled in a plex and dried out for hybridization capture for 16 h. Hybridized targets were then bound to streptavidin beads, and postcapture amplification was done for 15 cycles. As for whole-genome library preparation, enriched plexes were checked by High Sensitivity D1000 ScreenTape (Agilent, 5067-5584) on a 4200 TapeStation System (Agilent, G2991BA) and Qubit 3.0 fluorometer (Invitrogen, Q33216).

To filter out germline variants, participant-matched buffy coat DNAs collected from the UKGPCS trial were used. Buffy coat DNA (100 ng) was directly used for whole-genome library preparation using an NEBNext Ultra II FS DNA Library Prep kit for Illumina (New England Biolabs, E6177). Initially, enzymatic digestion was incubated for 20 min, and, after adaptor ligation, samples were identified using NEBNext Multiplex Oligos for Illumina (96 Unique Dual Index Primer Pairs Set 1, New England Biolabs, E6440L). Four PCR cycles were used for library enrichment.

For collection of cfDNA samples, 20 ml of whole peripheral blood was collected from each participant at each time point and stored in Cell-Free DNA Blood Collection Tubes (Streck, 218997). Plasma was separated from cells by centrifugation (1,600*g* for 10 min at room temperature), followed by a second centrifugation of the supernatant to remove all cell debris. Plasma was stored at −80 °C pending DNA extraction. cfDNA was extracted from plasma using a QIAamp circulating nucleic acid kit (Qiagen, 55114) according to the manufacturer’s protocol.

Whole-genome libraries were generated from 35 ng of cfDNA using a low-input NEBNext Ultra II DNA Library Prep kit for Illumina (New England Biolabs, E7645) and NEBNext Multiplex Oligos for Illumina (Unique Dual Index UMI Adaptors DNA set 1, New England Biolabs, E7395L), as for the FFPE samples described above. No fragmentation step was performed, and eight cycles of PCR were used for library enrichment. Elution was done in 38 µl of TE buffer (Invitrogen, 12090015), and quality control was checked, as described for the FFPE samples. Whole-genome libraries (190 ng) were used for whole-exome capture following Twist Exome 2.0 human panel’s protocol (Twist Biosciences).

### Sequencing

Sequencing was performed at three different levels: low-pass WGS, target sequencing or WGS according to the samples. Independent of the purpose, after pool quantification by Qubit and correct fragment size distribution by TapeStation, 2.5 nM product was sent for sequencing to the NovaSeq 6000 System (Illumina). Read length and depth was variable, as required by library composition. Sequencing was performed by the Institute of Cancer Research Tumor Profiling Unit.

First, 1 ng of up to 96 indexed whole-genome libraries was pooled for low-pass WGS. To reach the estimated coverage of at least 0.1× for copy number profiling, 50 paired-end reads were performed in an S2 flow cell.

Second, 12 enriched plexes (96 postcapture enriched libraries) were pooled together in equimolar amounts and sequenced at a median coverage after UMI compression of at least 100×, following 100 paired-end reads in an S2 flow cell.

With respect to the buffy coat libraries, WGS was performed for 150 paired-end reads in an S2 flow cell in pools of ten samples, for a minimum coverage of 30×. For those participants where buffy coats could not be taken, normal prostate tissue FFPE needle biopsy enriched libraries were sequenced following the same protocol as described above.

For the cfDNA samples, low-pass WGS and deep whole-exome sequencing were performed. For whole-exome sequencing, 100 paired-end reads were performed in an S4 flow cell in pools of a maximum of eight samples with a target coverage of a minimum of 200×.

### Multiplex immunohistochemistry

Multiplexed immunofluorescence images were acquired using an AKOYA Phenocycler-Fusion scanner (formerly known as CODEX) at a resolution of 0.5 µm per pixel. The multiplexed immunofluorescence panel consisted of 15 antibodies (Supplementary Table 9). Of those, CD4, CD8, CD20, CD3e, CD68, CD31, Ki67, PCK and TP63 were validated antibodies purchased directly from AKOYA. The remaining antibodies (FSP1, αSMA, vimentin, CD163, CK18 and PSA) were purified commercial antibodies that were manually conjugated. Following acquisition of the multiplexed immunofluorescence image, the same section was subsequently stained with H&E to enable direct comparison between tissue morphology and immunofluorescence markers. Images of the H&E-stained slides were acquired with a Phenocycler-Fusion scanner at a resolution of 0.5 µm per pixel. Staining intensity, observed within positively stained cells, was variable across our panel of markers. To account for those differences, intensity ranges were manually selected for each marker during visualization within AKOYA PhenoChart. Instances of autofluorescence were identified by visual inspection of the signal pattern and were excluded from the quantification of the marker abundance.

### Bioinformatics analysis

#### Buffy coat WGS analysis

FASTQ files were trimmed for adaptor content using Skewer^52^ with a minimum length allowed after trimming of 35 bp, keeping only reads with a minimum mean quality of 10 and removing highly degenerative reads (-l 35 -Q 10 -n). Trimmed reads were aligned to hg38 (GRCh38) using bwa mem^53^. SAM files were sorted and compressed to BAM files, and duplicates were marked using Picard tools (https://broadinstitute.github.io/picard/). When multiple FASTQ files were available for a sample, FASTQ files were initially processed separately but merged before marking duplicates using samtools (https://www.htslib.org/). BAM files were then indexed also using samtools.

#### Low-pass WGS analysis

FASTQ files were processed identically to the buffy coat WGS FASTQ files to the point of generating merged BAM files aligned to the human genome. BAM files were then processed using QDNAseq^54^ to convert read counts in 500-kilobase bins across the chromosomes of hg38 into log_2_ ratio data (log_2_ ratio of normalized coverage observed over expected, that is, raw copy number signal). The 500-kb bins for hg38 were generated according to QDNAseq instructions and normal BAM files from the 1000 Genomes Project (https://ftp.1000genomes.ebi.ac.uk; phase 3). Data normalization was performed in accordance with the QDNAseq workflow, including sex chromosomes. Bins were required to have a minimum mappability of 65 and 95% non-N bases. The smoothOutlierBins function step was removed as it artificially depressed highly amplified bins. The sqrt option was used for the segmentBins function. Log_2_ ratios in bins and segments were normalized by subtracting the median log_2_ ratio value of all bins.

To call absolute copy number, we used an adapted version of the ASCAT^55^ approach that leveraged using multiple sampling to search for ploidy solutions. For details, see Computational Analysis Supplementary Note. PGA was measured by calculating the fraction of bins not at the rounded baseline ploidy (this was expected to be half at sex chromosomes).

#### CNA phylogenetics

MEDICC2 (ref. ^56^) was used to generate phylogenetic trees based on CNA status. Bins were converted to genomic regions with equal copy number status across all samples using the run length encoder function in R (rle), and an artificial diploid root was generated. MEDICC2 was run using the –total-copy-numbers option to account for the lack of allele-specific copy number data. Only samples with a PGA of ≥0.01 were included in the trees. As MEDICC2 requires a minimum of two samples, trees were only created for 111/114 participants (both IMRT and DELINEATE).

#### Phylogenetic signal sidedness analysis

To investigate the distribution of left and right samples across the phylogenetic trees produced by MEDICC2, we used the phylogenetic signal function phylosig in the phytools R package^57^. If a sample was derived from the right side, it was assigned a trait value of 1, and left samples were assigned a value of 0; remaining samples were assigned 0.5. The diploid root was dropped as a sample in the tree. Phylosig was then run with the lambda method and the option of performing a hypothesis test. The tool was considered successfully run if the hypothesis test produced a *P* value (68/111 trees).

#### Focal amplification detection

We used multisample piecewise constant fitting segmentation to increase our sensitivity for detecting focal events; this was performed using multipcf in the copynumber package^58^. For individuals with a single sample, pcf was used. A penalty (gamma) of 15 was used for both functions. Segments with a *z* score greater than 3, occupying more than 3 but less than 20 bins (~10 Mb), were considered focally amplified. Genes present in the segments were calculated using bioMart (https://www.ensembl.org/) and cross-referenced with a set of prostate cancer-related oncogenes.

#### Genomic metric calculations from low-pass WGS

mPGA was calculated as the average PGA of all samples in a participant, not including samples with a PGA of <0.01. Maximum PGA was calculated as the maximum PGA observed in a participant. The Spearman metric was calculated as the mean pairwise Spearman’s *ρ* of the log_2_ ratio values (raw copy number signal) in the bins of all samples excluding those with a PGA of <0.01. The value was then subtracted from 1 to convert it from a measurement of homogeneity to heterogeneity to support interpretation. Lossness was calculated as the fraction of segments less than the rounded ploidy of the sample that did not overlap with the most distant telomeric or centromeric bin of each chromosome arm. Total events were calculated as the total number of CNA events present in the MEDICC2 phylogenetic tree produced for each participant. The number of subclonal events was the number of CNA events present in each tree after the most recent common ancestor (that is, excluding clonal events). Subclonality was calculated as the fraction of subclonal events as a proportion of total events.

#### UMI processing

FASTQ files from the same library were merged by concatenating the files. UMIs were processed using the fgbio pipeline (http://fulcrumgenomics.github.io/fgbio/). For details, see the Computational Analysis Supplementary Note.

#### Strand-split artifact read (SSAR) filtering

FFPE samples are affected by SSARs caused by single-stranded overhangs in fragments^59^. We filtered BAM files for reads demonstrating these characteristics by realigning the UMI consensus reads using bwa mem with a minimum seed length of 10 (-k), not outputting alignments with a score lower than 10 (-T). Reads with secondary alignments on the complementary strands within a window of 500 bp were flagged as SSAR reads and removed from the consensus UMI BAM file using Picard tools. Duplicates were marked again with Picard tools, and the BAM file was indexed with samtools.

#### Quality control

Targeted panel sequencing samples with a mean target coverage of less than 10× as calculated by the CollectHsMetrics option in Picard tools were considered failed. The read error rate was assessed before and after compression using ErrorRateByReadPosition in the fgbio library. Failed low-pass WGS samples were determined by manual inspection of the log_2_ ratio profiles. For all data, mismatching samples were identified using the CheckFingerprint option in the Genome Analysis Toolkit (GATK)^60^ using references generated by HaplotypeCaller and dbSNP 146. FFPE damage was assessed using mapDamage^61^, and FASTQ and BAM qualities were assessed using FASTQC (https://www.bioinformatics.babraham.ac.uk/projects/fastqc/) and Qualimap2 (ref. ^62^).

#### Somatic mutation calling

We initially called somatic mutations per sample using mutect2 (ref. ^63^) in GATK with the matched buffy coat WGS from the participant or a normal tissue targeted panel sequencing sample as a normal reference. Mutation calling was limited to the coordinates of the genes on the panel. The output was filtered using FilterMutectCalls, and mutations were kept only if the coverage in both the tumor and normal tissue was greater than ten reads and the variant was present in three or more reads in the tumor. The variant must have the genotype ‘0/0’ in the normal tissue but must not in the tumor. Mutations with the flag ‘artifact_in_normal’ were kept, but variants called in each tumor sample were removed if their VAF was less than ten times greater than in the normal sample.

Resulting VCF files were then merged using vcf-merge (https://vcftools.github.io/) and used as input for platypus^64^ run in genotyping mode (–getVariantsFromBAMs = 0). The following criteria were used for an initial round of filtering for high-quality mutations: (1) mutations with the poor mapping quality (MQ) and strand bias (strandBias) flags were removed, (2) mutations were required to have a genotype quality of at least 60 in one sample, (3) a minimum of ten reads at the site was required in all samples, (4) the germline sample was required to have a genotype of ‘0/0’ and at least one tumor sample could not have a genotype of ‘0/0’, (5) a minimum of three reads covering the variant in at least one of the tumor samples per participant was required, and (6) the highest VAF in the tumor samples had to be ten times greater than the VAF in the normal tissue. Variants were annotated using VEP (https://www.ensembl.org/).

Additionally, to flag high-quality SNVs, we separately called mutations using deepSNV^65^, as performed previously^66^. Details of implementation and further filtering are provided in the Computational Analysis Supplementary Note. Mutations were considered subclonal if the VAF was not greater than 0.05 in all samples. Subclonality assessment of mutations in participants with fewer than three tumor samples with targeted panel data was only presented in the heat map in Fig. 2a.

#### dN/dS analysis

dN/dS analysis was performed using dNdScv^67^. Sample B11 in FD-002 was excluded from the analysis as it contained an abundance of synonymous mutations. All participants with available data were included in the ‘All’ category, whereas only participants with a minimum of three tumor samples with targeted panel data were included when assessing ‘Clonal’ and ‘Subclonal’ mutations. dN/dS was considered significantly greater than 1 (neutral) when the lower bound of the 95% CI was greater than 1 and vice versa.

#### Calculating the number of mutated copies and loss of heterozygosity

The number of mutated copies is estimated using a rearranged cancer cell fraction equation that considers sample purity, the total copy number of the mutation site and the VAF and assumes that the cancer cell fraction is equal to 1 (clonal). The mutation is homozygous if the estimated number of mutated copies is greater than the total copy number minus 0.5.

#### cfDNA low-pass analysis

Low-pass samples derived from cfDNA were processed from raw data to alignment as described previously for the primary tissue samples. However, before processing BAM files using QDNAseq, BAM files were filtered for reads for an insert size between 90 and 150 bp to enrich for tumor fragments. Samples were segmented using multipcf from the package copynumber, if multiple time points were available (*γ* = 10), to enable more sensitive detection of CNAs in impure samples. If only a single time point was available, the pcf function was used (*γ* = 10).

Copy number fits were calculated using the ASCAT equation excluding B-allele frequency, as for the primary samples; however, the minimum purity was set to 0.01, and a ploidy range between 1.5 and 4.7 was searched. This was narrowed between 4 and 4.7 for FI-072. The fit for FI-057 cfDNA TP1 was manually set (purity = 0.07, ploidy = 4.41). MEDICC2 was rerun for participants with cfDNA samples as previously described.

#### cfDNA whole-exome sequencing analysis

Whole-exome sequencing data from cfDNA were analyzed using the fgbio pipeline as for the primary tissue samples; however, we used a NextFlow implementation (https://github.com/chelauk/nf-core-umialign). For details, see the Computational Analysis Supplementary Note.

### Computational histopathology

#### Whole-slide image acquisition

Digital whole-slide images of diagnostic H&E slides were acquired using a Zeiss AxioScan.Z1 slide scanner. Slides were scanned at a resolution of 0.11 µm per pixel. For compatibility with the deep learning models, images were subsequently rescaled to 0.22 µm per pixel or an equivalent of a 40× magnification.

#### Automated Gleason segmentation and grading

We trained a deep learning classifier to segment the glandular regions of a tissue section according to their Gleason pattern. The U-Net style classifier^68^ (Extended Data Fig. 5c) was trained on image patches generated from hand-drawn gland regions, each labeled as normal, PIN, Gleason 3, Gleason 4 or Gleason 5. From 42 whole-slide images within the IMRT trial cohort, a total of 3,168 gland regions were annotated, representing an equivalent of 65.47 mm^2^ of tissue. Thirty-four whole-slide images were used to train the model, and eight were withheld for validation. To generate suitable input for the classifier, annotated regions were converted into image patches with associated segmentation masks (Extended Data Fig. 5b).

The classifier uses a multiresolution representation of the tissue to segment the glands. As such, each input image patch was composed of a pair of 500 × 500 pixel images, representing a region of the tissue at a resolution of 0.44 µm per pixel and 0.88 µm per pixel or an equivalent 20× and 10× magnification, respectively (Extended Data Fig. 5e). These images were subsequently resized to 224 × 224 pixels to match the desired input size of the model. The classifier’s output was a set of probability maps, representing the segmentation of the 0.44 µm per pixel image. There were six output maps in total, corresponding to the five gland types and a sixth for no gland detected (Extended Data Fig. 5e). Due to the softmax final layer, these maps sum to 1 for every pixel. The final segmentation is produced by assigning to each pixel the label with the largest probability. For the final analysis, the normal and PIN labels were merged under a single ‘benign’ label.

To aid comparison with pathologists’ assessments, we also developed an algorithm to convert the resultant Gleason segmentation map into a standard primary and secondary Gleason score (see the Supplementary Computational Histopathology Analysis Note). Each section’s Gleason score was subsequently converted into an ISUP grade group using the 2014 criteria. Patient-level grade group was computed for each participant by taking a weighted mean of their individual slide grade groups and rounding down. When computing the mean, each slide was weighted by the area that was segmented as tumor (Gleason pattern 3, 4 or 5).

#### Automated cell classification

We trained an SCCNN-style DenseNet classifier^69,70^ to detect all cell nuclei within the tissue section and label them with their associated type. In the classifier’s raw output, cells were partitioned into five categories: epithelial, stromal, acute immune, chronic immune and unknown. However, for the final analysis, chronic and acute immune cells were merged under a single ‘immune’ label. The classifier was trained on image patches generated from 40,634 hand-annotated cells from 56 whole-slide images. Forty-nine whole-slide images were used directly for training, and seven were withheld for validation. The majority of the training dataset was taken from PROMIS, an external cohort of prostate cancer specimens. However, an additional set of 9,682 annotations from the IMRT trial cohort were added to the dataset to improve classification accuracy. These were intended to address cohort-level visual differences due to differences in section preparation, tissue staining and model of slide scanner used to acquire the images.

#### Gleason Morisita index

In conjunction with the output of the Gleason classifier, epithelial cells were further classified into normal, PIN, Gleason 3, Gleason 4 and Gleason 5 epithelial cells (Extended Data Fig. 5g). From these reclassified cells, the Gleason Morisita index for a slide was computed. Specifically, the Gleason Morisita index is defined as the Morisita index^26^ between epithelial cells belonging to the primary and secondary Gleason patterns of the section, as assessed by the automated classifier. Polygons for the Morisita index were generated using Voronoi tessellation. Sections where the primary and secondary patterns were assessed to be the same (for instance, 4 + 4), the Gleason Morisita index was considered to be 0. At the patient level, the Gleason Morisita index was computed as the median value across all slides from the participant that were determined to be cancer by the automated classifier.

To evaluate the robustness of Gleason Morisita to different implementations of the method, we also propose two alternatives: (1) compute the Morisita index directly on the Gleason segmentation maps rather than on the subclassified epithelial cells and (2) use a 50 × 50 grid of rectangular regions rather than a set of Voronoi regions. Both alternative metrics are seen to be well correlated with the version of the metric proposed in this work and also produced similar predictions for time to recurrence (Extended Data Fig. 7). For more details, please refer to the Computational Analysis Supplementary Note.

### Comparison of bioinformatics and computational histopathology

Continuous Gleason of a section was calculated as the mean of the automated Gleason segmentation weighted by the raw number of segmented pixels of each pattern (Gleason 3, 4 or 5). Chromosome arms were considered gained or lost if their median copy number was greater than or less than the baseline copy number, respectively. Mixed effects linear models were produced for each chromosome arm, for gains and losses separately, with neutral (baseline) copy number as the reference. This was performed using both continuous Gleason and Tumor-Immune Morisita as dependent variables in separate analyses with participants as a group effect term. Models were only produced if there were more than ten observations of the loss or gain. The *P* values were recorded for the gradient (m) and were adjusted using the Benjamini–Hochberg method for each dependent variable separately. The TSG-OG scores for each chromosome arm were derived from Davoli et al.^31^. The Joint Diversity metric was calculated as the square root of the Spearman metric multiplied by the patient-level Gleason Morisita.

### Outcome analysis

Outcome analysis was only performed on participants from the IMRT trial to ensure clinical homogeneity. For genomic analysis, only participants with three or more low-coverage WGS samples with a PGA of ≥0.01 were used to ensure that all metrics would be available to test. When considering mutation data, participants with fewer than three tumor samples with targeted panel data were also excluded. For computational pathology analysis, all samples assessed as benign by the automated classifier were excluded. The R package survival was used to perform the outcome analysis, and the package survminer was used to generate forest plots.

### Univariate analysis

To determine the metrics to be used in the multivariate CPH model, candidate metrics were first tested in a univariate CPH model. DNA damage mutations were tested by their clonality status using wild type as a reference. For mPGA, maximum PGA, lossness, total events and number of subclonal events, the natural log of the metric was used. For subclonality, the exponent of the metric was used. For all other continuous metrics, the raw value of the metric was used. All continuous metrics were also tested as binary variables in a univariate model by splitting the cohort at a chosen threshold value. Spearman was split at the upper tertile, and all other metrics were split at the median. Metrics with a *P* value of <0.1 were included in the multivariate analysis per outcome. In the event that both the continuous and binary version of the metric qualified, only the continuous variable was included in the multivariate model.

### Multivariate analysis

Qualifying metrics were then included in a multivariate Cox model alongside clinical covariates (PSA > 20 ng ml^–1^, ISUP grade group (reviewing pathologist), T3+ and N1+) and number of samples per participant. In the sequencing cohort, this was defined as the number of samples with a PGA of ≥0.01. In the imaging cohort, this was defined as the number of samples graded as cancer by the automated classifier. All continuous variables are linearly rescaled such that the 5th and 95th percentiles have values of 0 and 1, respectively. ISUP grade groups, both according to the reviewing pathologist and the automated classifier, used grade group 5 as the reference. To avoid potential issues relating to variable dependence, ISUP grade group (automated classifier) was tested in a separate multivariate model, with Gleason Morisita and ISUP grade group (reviewing pathologist) excluded.

### Statistical analysis

All statistical analyses related to the genomics data were performed in R. The lmerTest package was used to perform mixed effects linear modeling. All box plots show the center line as the median and box limits as upper and lower quartiles. Whiskers extend no further than 1.5× interquartile range past the box limits, and points represent outliers. Forest plots show 95% CI of HRs, and the covariate *P* values are derived from a Wald test. All statistical tests were two sided unless otherwise stated.

### Reporting summary

Further information on research design is available in the Nature Portfolio Reporting Summary linked to this article.

## Supplementary information

Supplementary Information Supplementary Note Fig. 1 and Computational Analysis Supplementary Note.

Reporting Summary

Supplementary Table S1. IMRT trial imaging and sequencing cohorts. S2. Somatic CNAs from low-pass WGS. S3. Targeted gene panel. S4. Mutations and deletions from targeted sequencing with inferred loss-of-heterozygosity status. S5. Statistics associated with the CPH model presented in Fig. 3d. Lower and upper represent 95% CIs. Lower, upper and *P* values are derived from a Wald test; *q* values represent false discovery rate calculated using the Benjamin–Hochberg method. S6. Statistics associated with the CPH model presented in Fig. 4e. Lower and upper represent 95% CIs. Lower, upper and *P* values are derived from a Wald test; *q* values represent false discovery rate calculated using the Benjamin–Hochberg method. S7. Percentage of immune cells per sample in the imaging cohort, as assessed by automated cell classifier. S8. Statistics associated with the CPH model presented in Fig. 5h. Lower and upper represent 95% CIs. Lower, upper and *P* values are derived from a Wald test. S9. Optimized panel of antibodies used for multiplex immunohistochemistry. S10. Immune profile as assessed by multiplex immunohistochemistry.

## Source data

Source Data Fig. 2 Statistical source data.

Source Data Fig. 3 Statistical source data.

Source Data Fig. 4 Statistical source data.

Source Data Fig. 5 Statistical source data.

Source Data Extended Data Fig. 1 Statistical source data.

Source Data Extended Data Fig. 2 Statistical source data.

Source Data Extended Data Fig. 3 Statistical source data.

Source Data Extended Data Fig. 4 Statistical source data.

Source Data Extended Data Fig. 6 Statistical source data.

Source Data Extended Data Fig. 7 Statistical source data.

**Extended Data Fig. 8:**
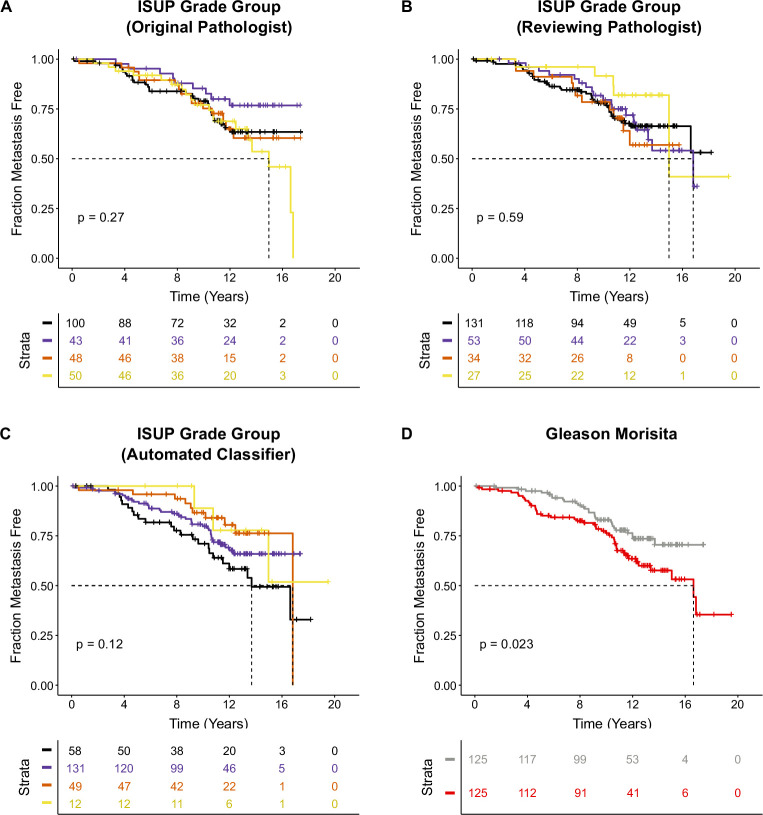
Univariate KM curves of time to metastasis for Gleason grading and Gleason Morisita. (**A**) Gleason grading from original reporting pathologists (Imaging Cohort, n = 250 participants, two-sided log-rank test, χ^2^ = 7.05, d.f. = 3, *P* = 0.27). (**B**) Gleason grading from reviewing pathologist (Imaging Cohort, n = 250 participants, two-sided log-rank test, χ^2^ = 2.28, d.f. = 3, *P* = 0.59). (**C**) Gleason grading from automated classifier (Imaging Cohort, n = 250 participants, two-sided log-rank test, χ^2^ = 5.83, d.f. = 3, *P* = 0.12). (**D**) Gleason Morisita index, split by median. Only Gleason Morisita index shows a significant difference in time to metastasis (Imaging Cohort, n = 250 participants, two-sided log-rank test, χ^2^ = 5.17, d.f. = 1, *P* = 0.023). Source data

Source Data Extended Data Fig. 8 Statistical source data.

**Extended Data Fig. 9:**
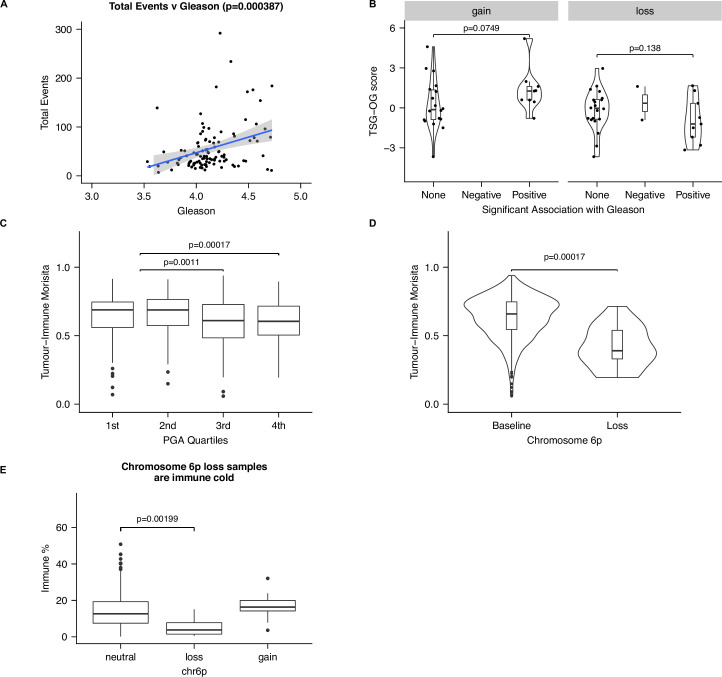
Associations of genomic markers with Digital Pathology analysis. (**A**) Comparison of continuous Gleason values (n = 106 participants) and the total number of phylogenetic CNA events. Trees with more events had a greater continuous Gleason value (linear model, 2-sided t-test on gradient, estimate = 63.7, standard error = 17.36, t = 3.67, d.f. = 104). Shaded area represents 95% confidence interval. (**B**) Association of TSG-OG score and the correlation of chromosome arm changes with continuous Gleason. Each data point represents the TSG-OG score (higher indicates more oncogene rich, lower indicates more tumour suppressor rich, Davoli et al.^31^) for a chromosome arm. Arms are then categorised according to if the test compared a gain or loss of the arm to the baseline level. Values are further categorised by whether the arm gain or loss is associated with no change in continuous Gleason (None, gain n = 18 arms, loss n = 20 arms), a reduction in continuous Gleason (Negative, loss n = 2 arms) or an increase in continuous Gleason (Positive, gain n = 9 arms, loss n = 9 arms). P-values are derived from one sided t-tests (gain, standard error = 0.71, d.f. = 18.6, t = −1.5; loss, standard error = 0.67, d.f. = 14, t = 1.1). Boxplots show centre line as median, box limits as upper and lower quartiles, whiskers extend no further than 1.5x interquartile range past the box limits and points represent outliers. (**C**) Samples present in the upper two quartiles (n = 138 samples for each quartile) of PGA values are associated with reduced Tumour-Immune Morisita when separately compared to the first two quartiles combined (2-sided Mann-Whitney U tests, W = 15312, 3rd Quartile; W = 14728, 4th Quartile). Boxplots show centre line as median, box limits as upper and lower quartiles, whiskers extend no further than 1.5x interquartile range past the box limits and points represent outliers. (**D**) Chromosome 6p loss (n = 20 samples) is associated with a lower Tumour-Immune Morisita compared to those with baseline CN (n = 516 samples). Linear mixed effects model, 2-sided t-test on gradient, estimate = −0.16, standard error = 0.04, d.f. = 458, t = −3.8. P-value is not adjusted for multiple hypothesis testing. Boxplots show centre line as median, box limits as upper and lower quartiles, whiskers extend no further than 1.5x interquartile range past the box limits and points represent outliers. (**E**) Chromosome 6p loss samples (n = 20 samples) have a significantly lower percentage of immune cells than those with a baseline chromosome 6p copy number (n = 516 samples, linear mixed effects model, 2-sided t-test on gradient, standard error = 2.01, d.f. = 525, t = −3.1). 16 samples had a gain in chromosome 6p. Boxplots show centre line as median, box limits as upper and lower quartiles, whiskers extend no further than 1.5x interquartile range past the box limits and points represent outliers. Source data

**Extended Data Fig. 10:**
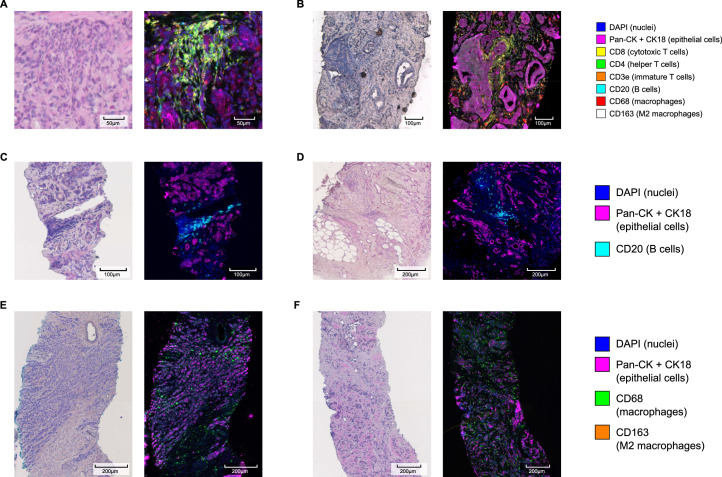
Supplementary examples of immune profiles in the multiplex immunohistochemistry cohort. Each image pair shows the expression of immunofluorescence markers (right), acquired by Phenocycler Fusion, with the matched region from H&E staining (left). (**A**, **B**) Examples of immune hot regions. (**C**, **D**) Examples of regions abundant for CD20 (cyan). (**E**, **F**) Examples of regions abundant for CD68 (green). CD163 (orange) is rarely seen, suggesting that these are likely to be solely M1 macrophages. Immunohistochemistry experiments were run once following optimisation and validation.

Source Data Extended Data Fig. 9 Statistical source data.

## Data Availability

Additional analyzed data are available on Mendeley (https://data.mendeley.com/datasets/cd9cf2fb76). Sequence data have been deposited at the European Genome–Phenome Archive (EGA), which is hosted by the EBI and the CRG, under accession numbers EGAS00001006096 (tumor data) and EGAS00001006098 (normal data). Further information about EGA can be found on https://ega-archive.org. Access to the anonymized clinical data and digitized H&E slide images from the study can be granted through a request to the corresponding author and completion of a Data Access Form. Proposals will be reviewed by the corresponding author and Trial Translational Group on the basis of scientific merit, ethical review, available resources and regulatory requirements. Once approved, requested data will be made available for the proposed work. A steering committee will have the right to review and comment on any draft papers based on the data before publication. Source data are provided with this paper. Source data for Supplementary Note Fig. 1 are available at the Mendeley link above.

## Code availability

Code is available at GitHub at https://github.com/stars/ntrahearn/lists/forecast.
