# Supplementary material for: Tumor evolution metrics predict recurrence beyond 10 years in locally advanced prostate cancer
Source: Nat Cancer. 2024 Jul 12;5(9):1334–51. doi: 10.1038/s43018-024-00787-0 (PMC11424488; doi:10.1038/s43018-024-00787-0)
Supplement: Supplementary file 1 — Supplementary Note Fig. 1 and Computational Analysis Supplementary Note. [file 43018_2024_787_MOESM1_ESM.pdf]

nature cancer

Supplementary information

https://doi.org/10.1038/s43018-024-00787-0

Tumor evolution metrics predict recurrence beyond 10 years in locally advanced prostate cancer

In the format provided by the authors and unedited

Table of Contents

1. **Supplementary Note Figure** **1**
2. **Supplementary Note Methods** **29**

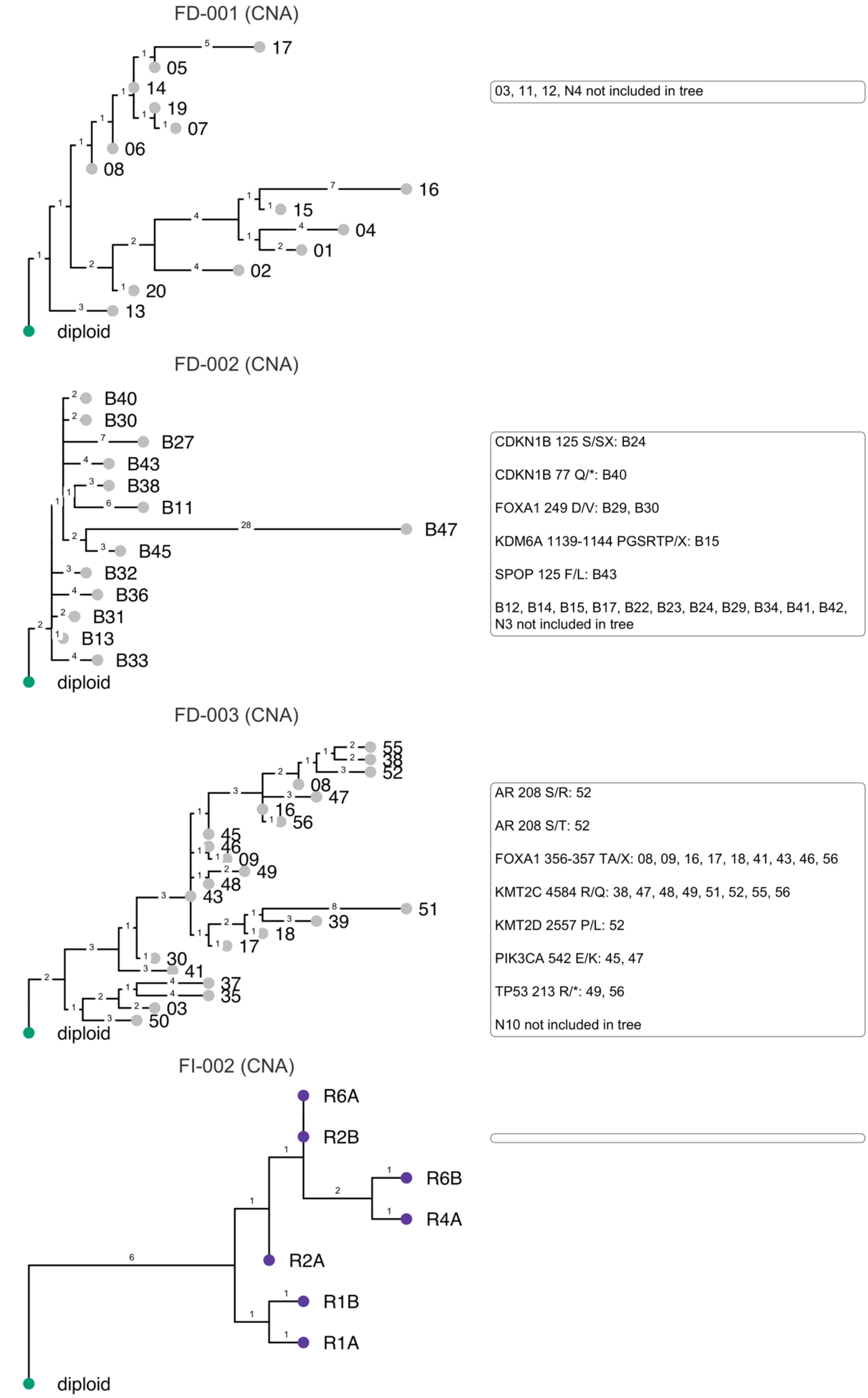

Supplementary Note Figure 1 continued on next page.

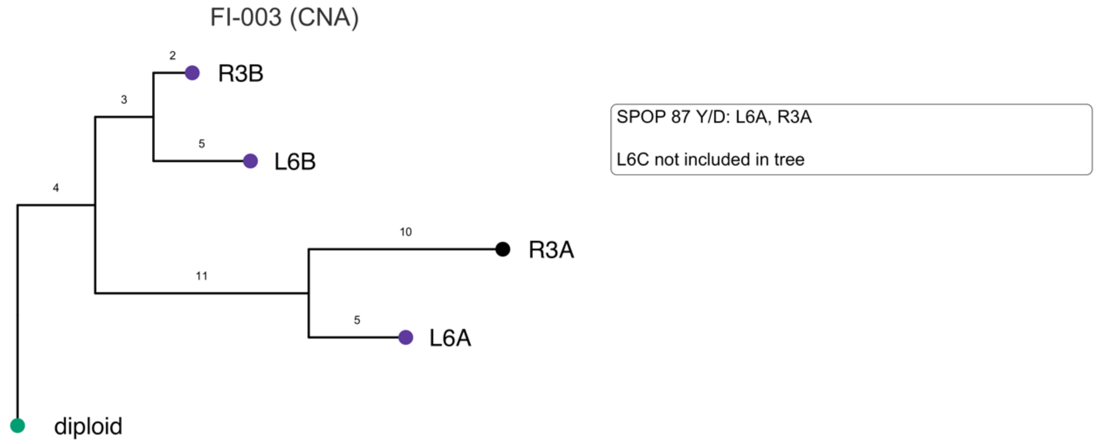

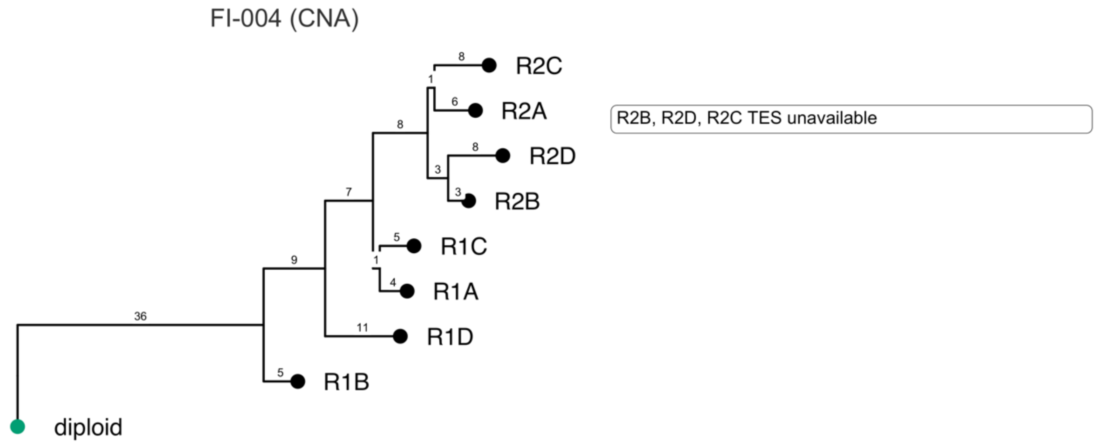

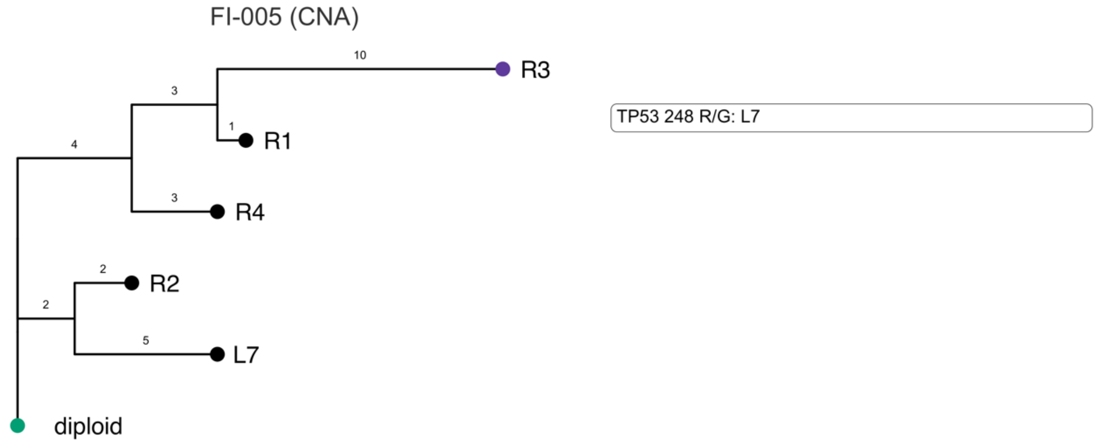

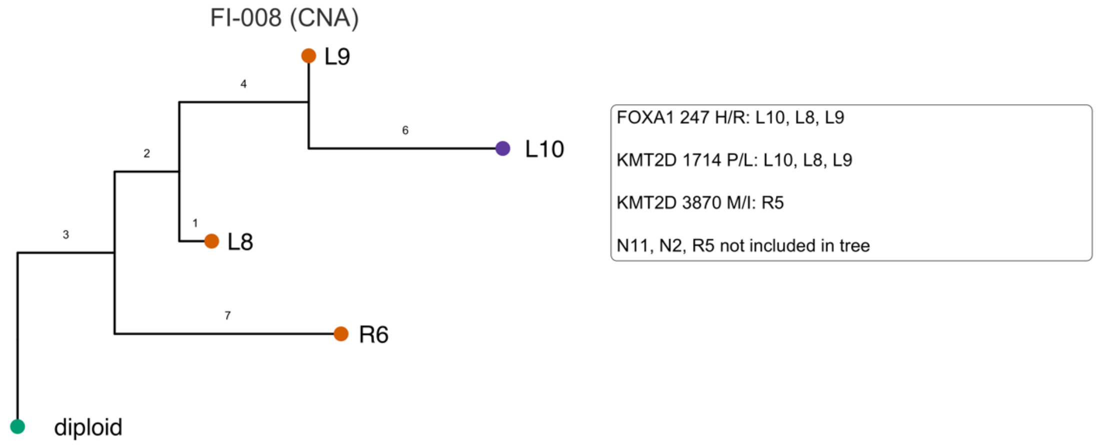

Supplementary Note Figure 1 continued on next page.

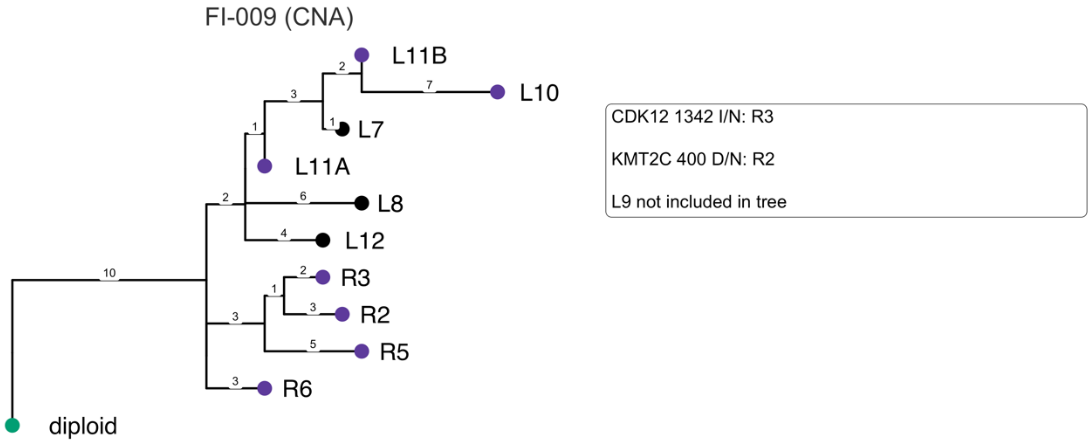

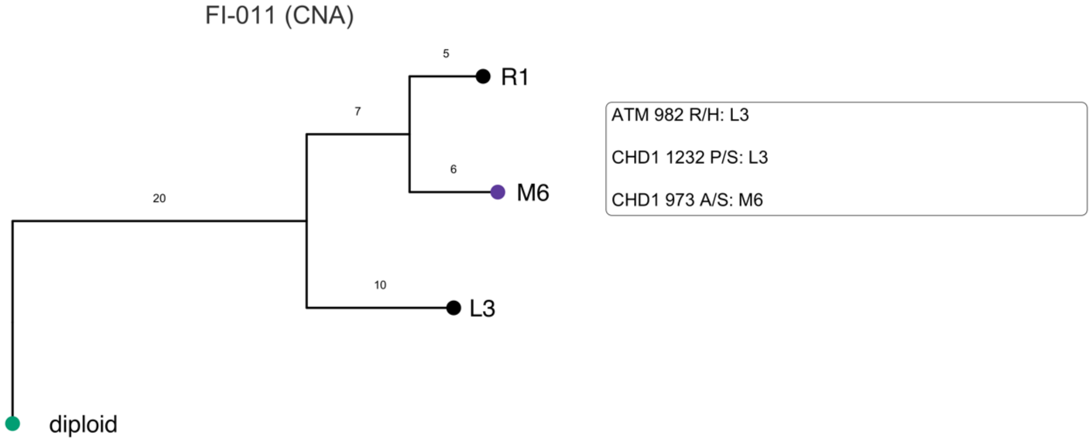

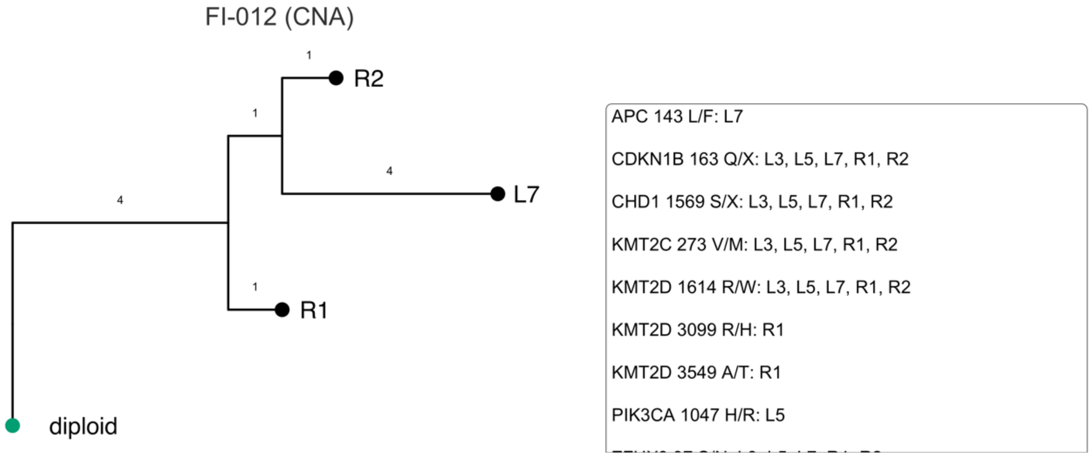

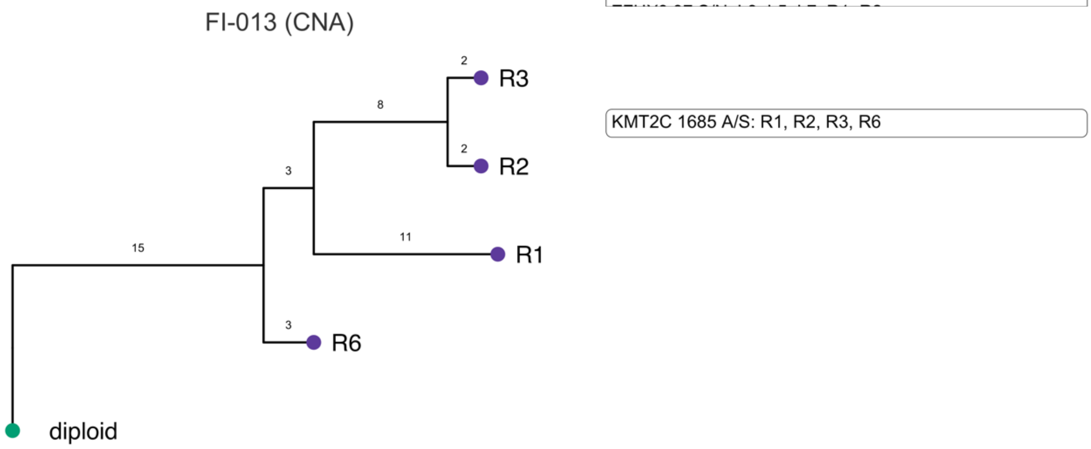

Supplementary Note Figure 1 continued on next page.

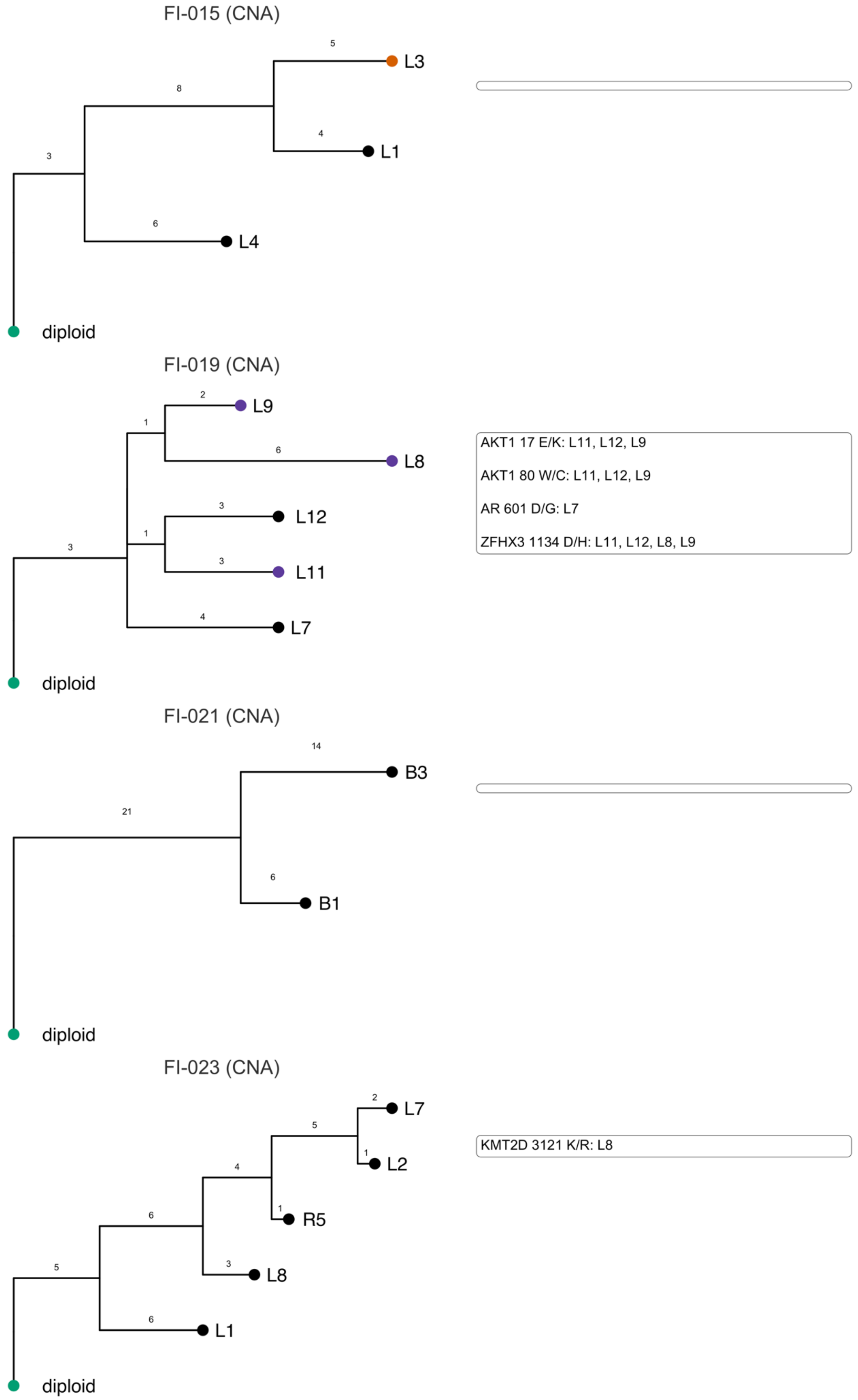

Supplementary Note Figure 1 continued on next page.

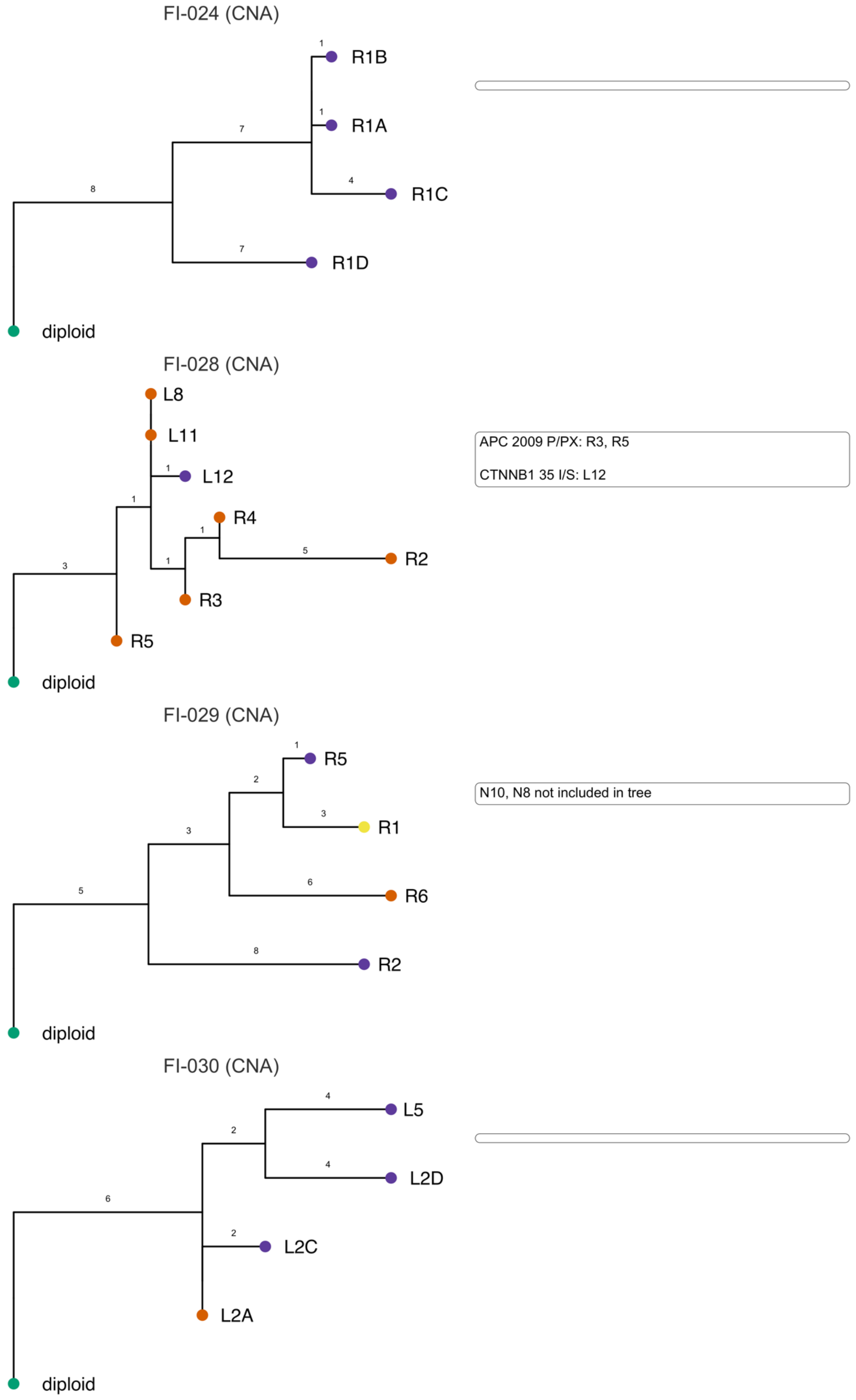

Supplementary Note Figure 1 continued on next page.

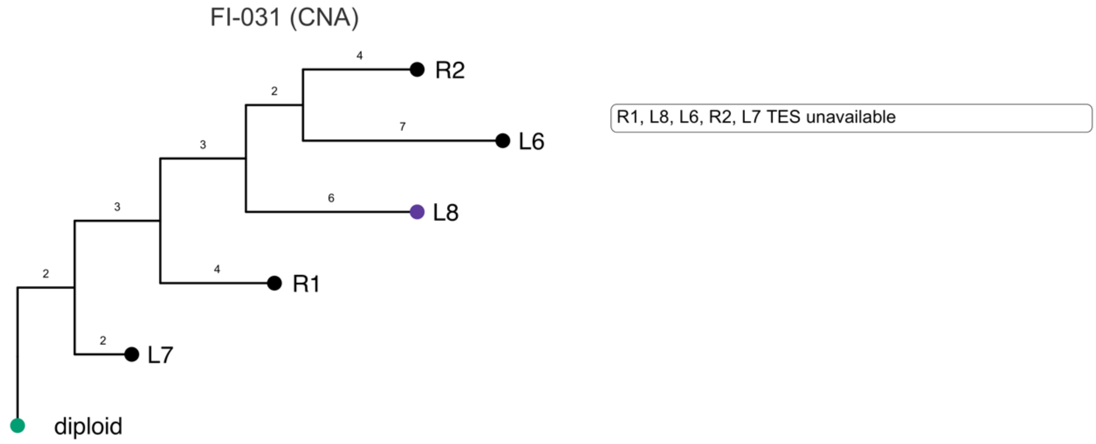

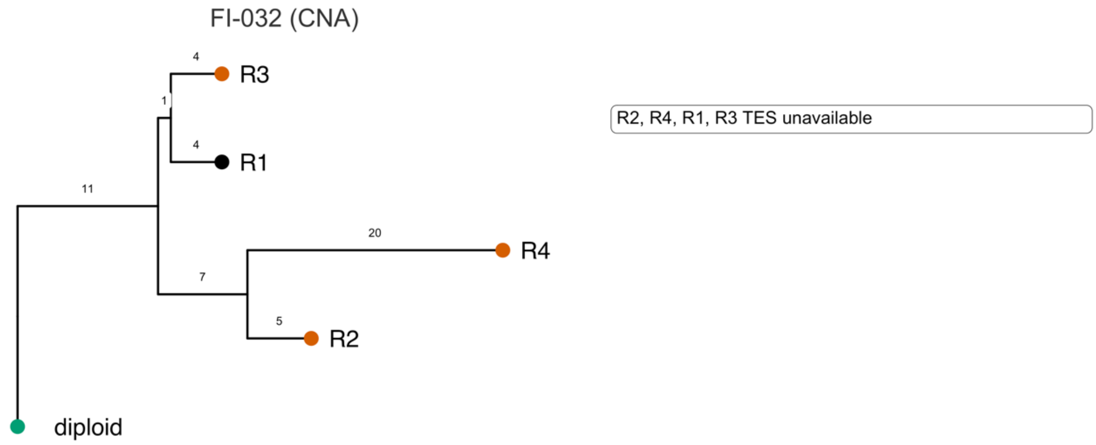

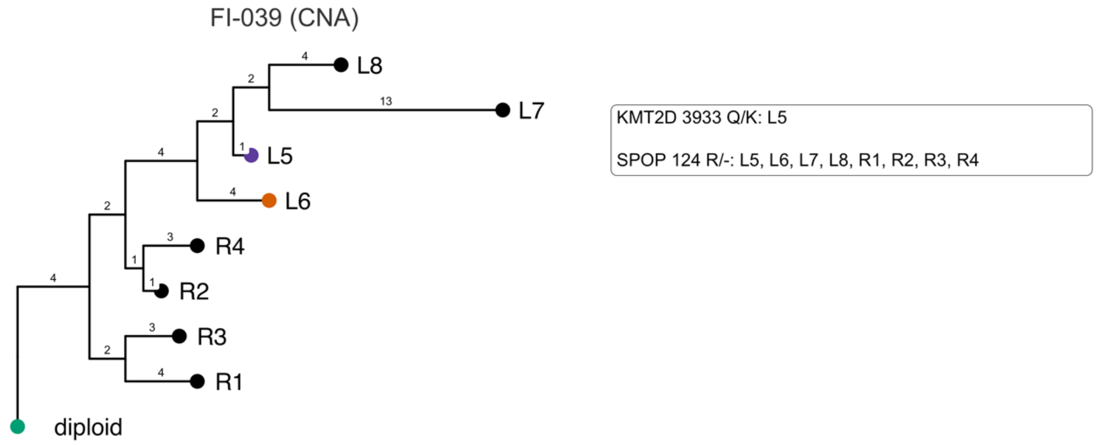

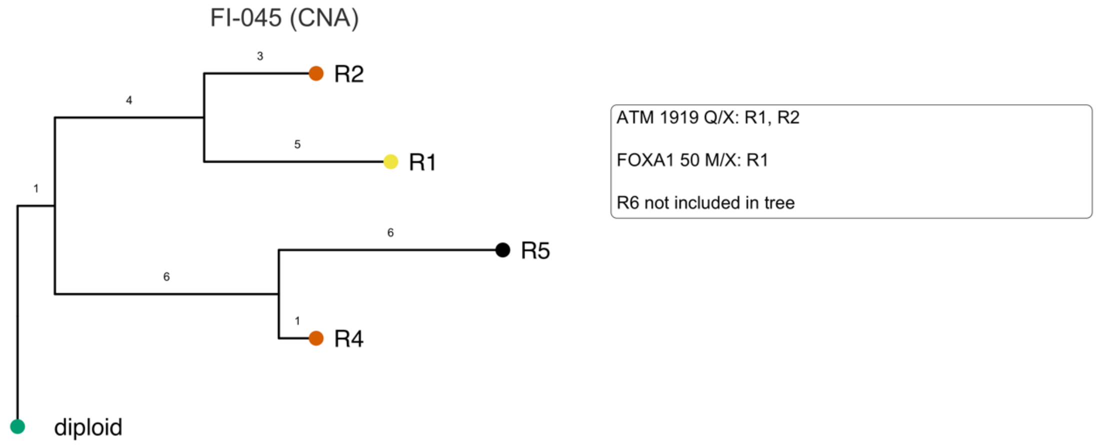

Supplementary Note Figure 1 continued on next page.

FI-048 (CNA)

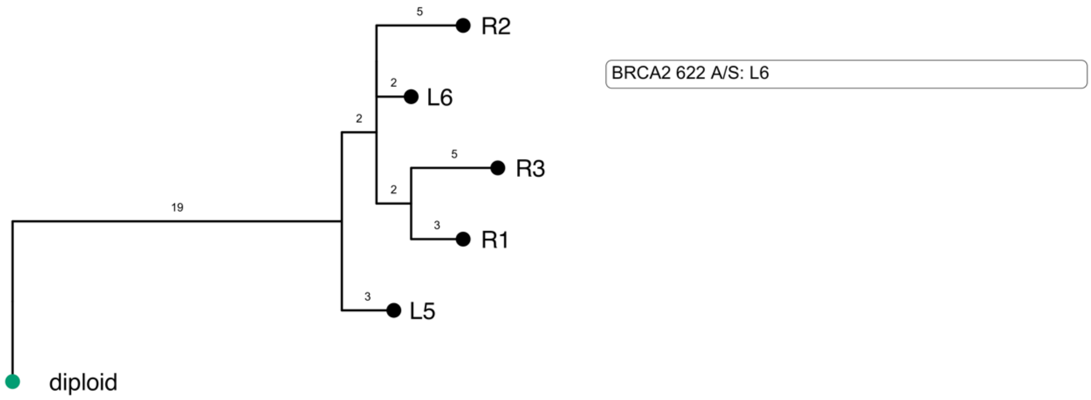

FI-049 (CNA)

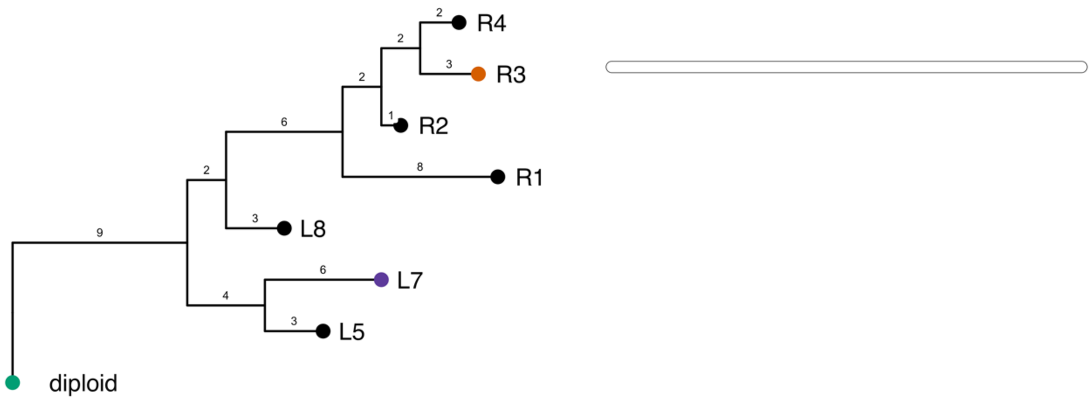

FI-051 (CNA)

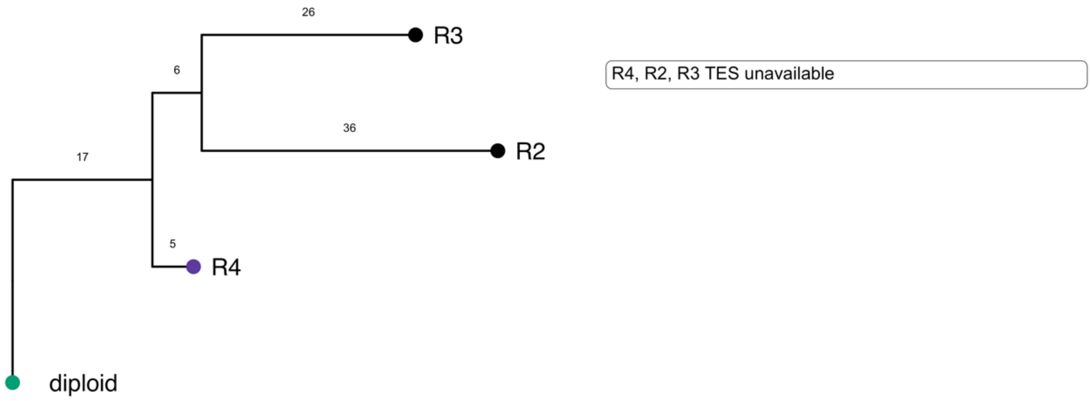

FI-052 (CNA)

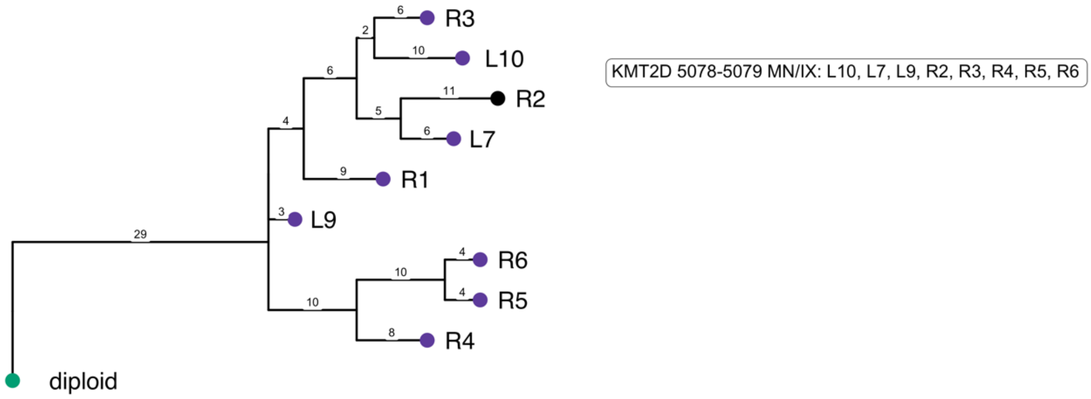

Supplementary Note Figure 1 continued on next page.

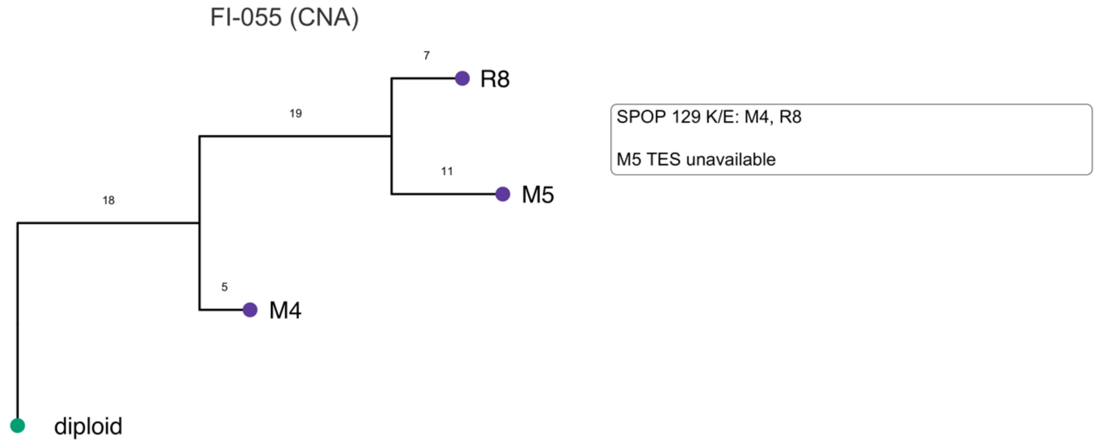

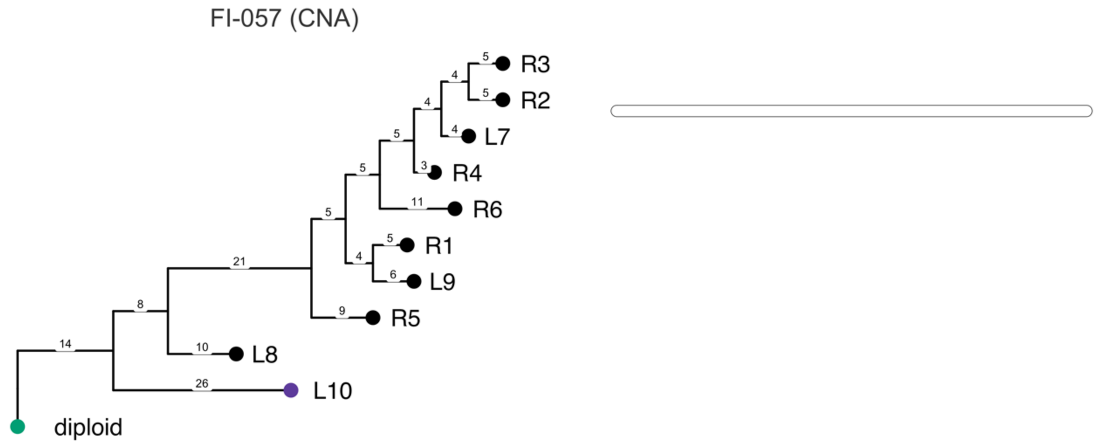

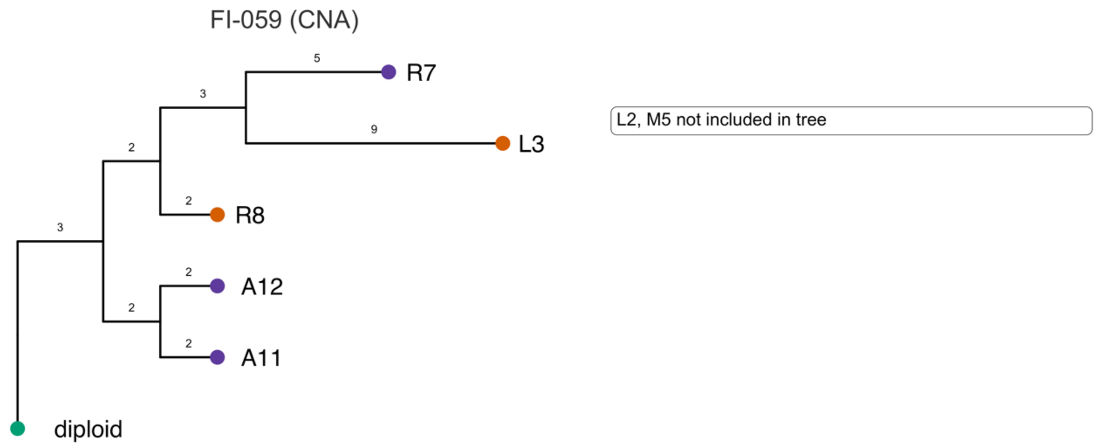

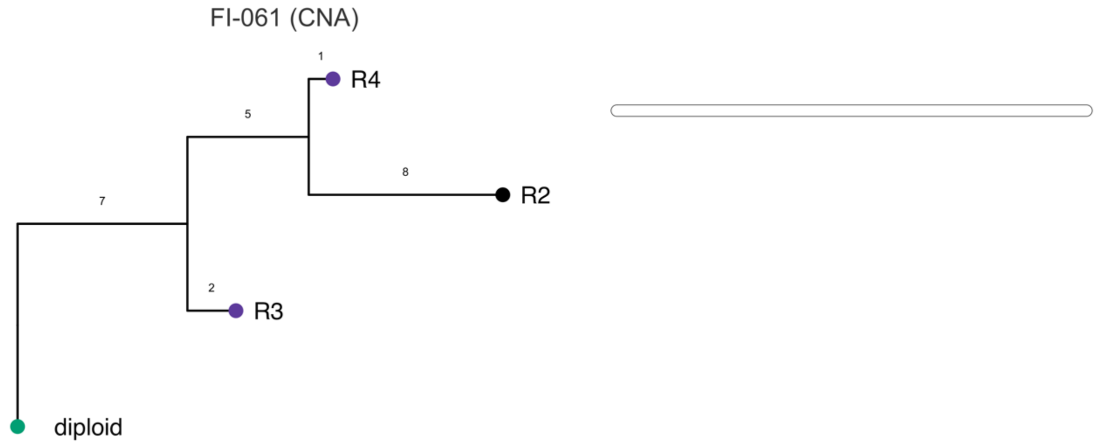

Supplementary Note Figure 1 continued on next page.

FI-065 (CNA)

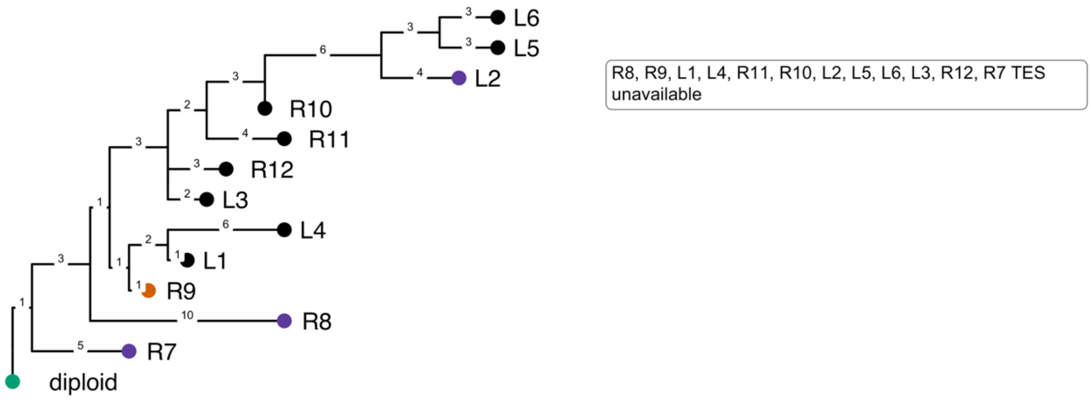

FI-070 (CNA)

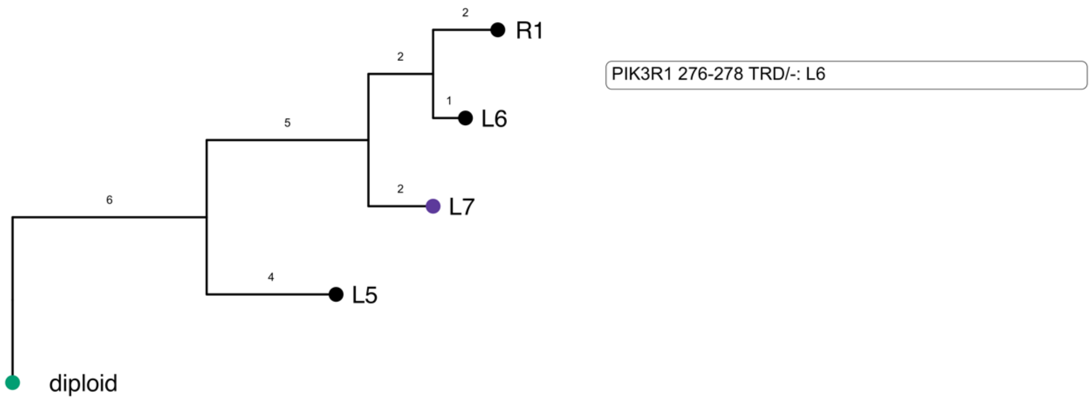

FI-072 (CNA)

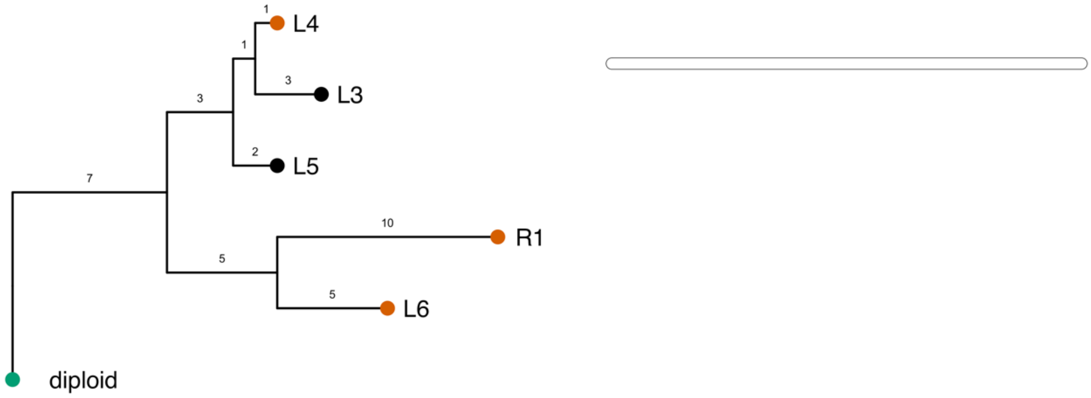

FI-075 (CNA)

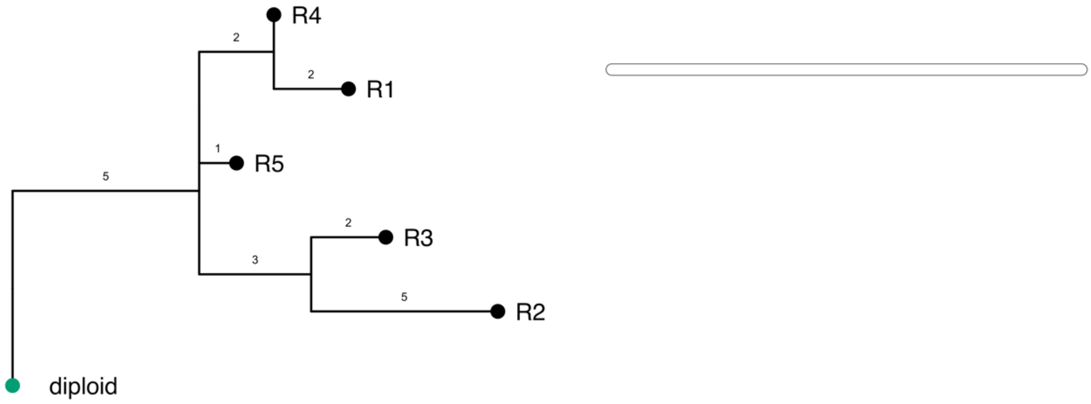

Supplementary Note Figure 1 continued on next page.

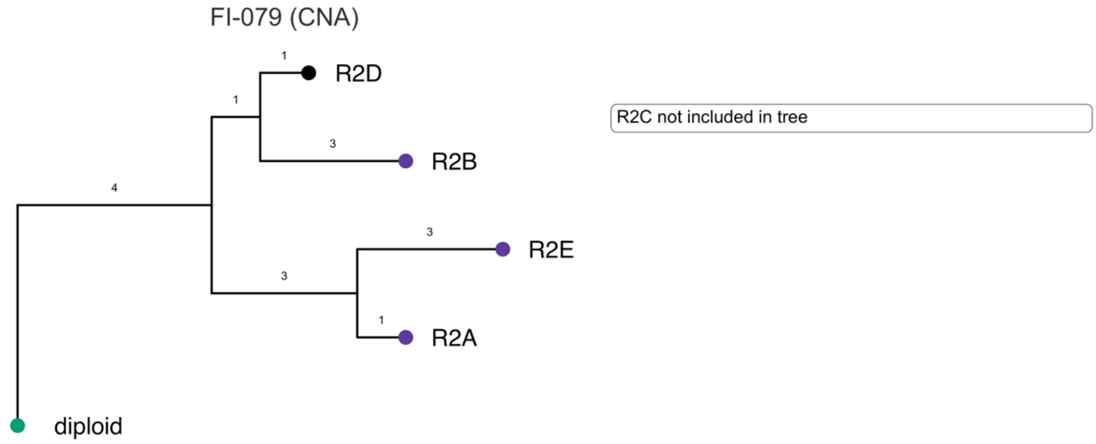

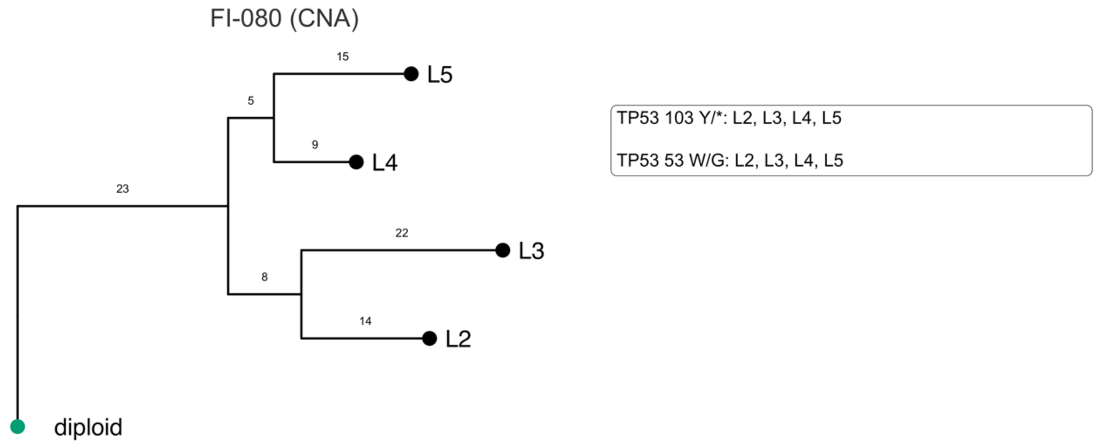

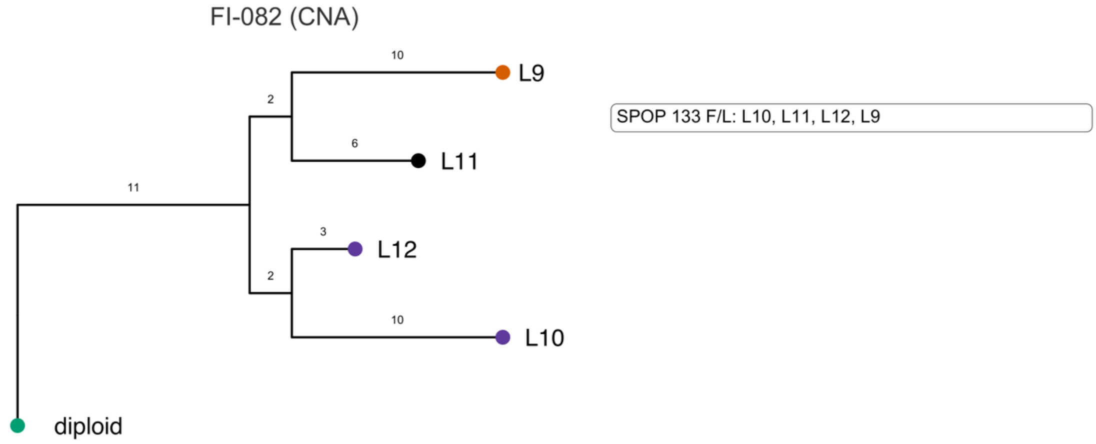

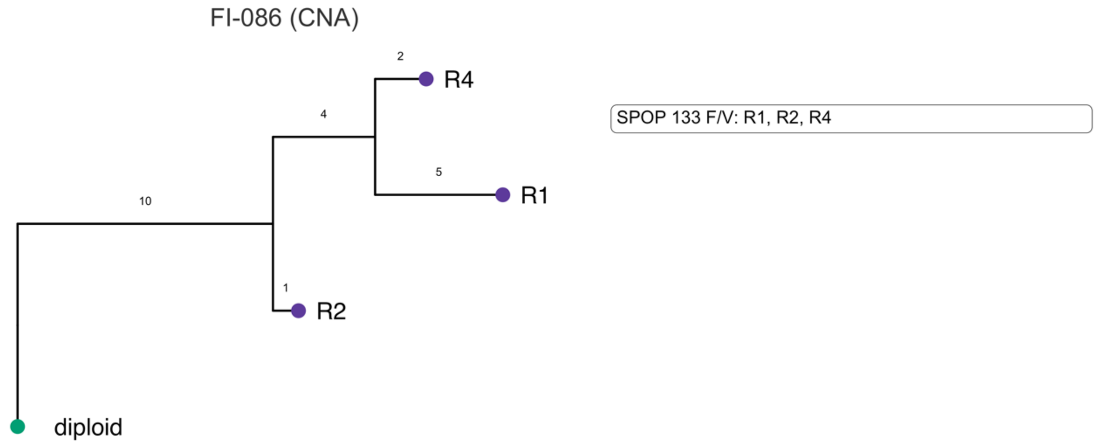

Supplementary Note Figure 1 continued on next page.

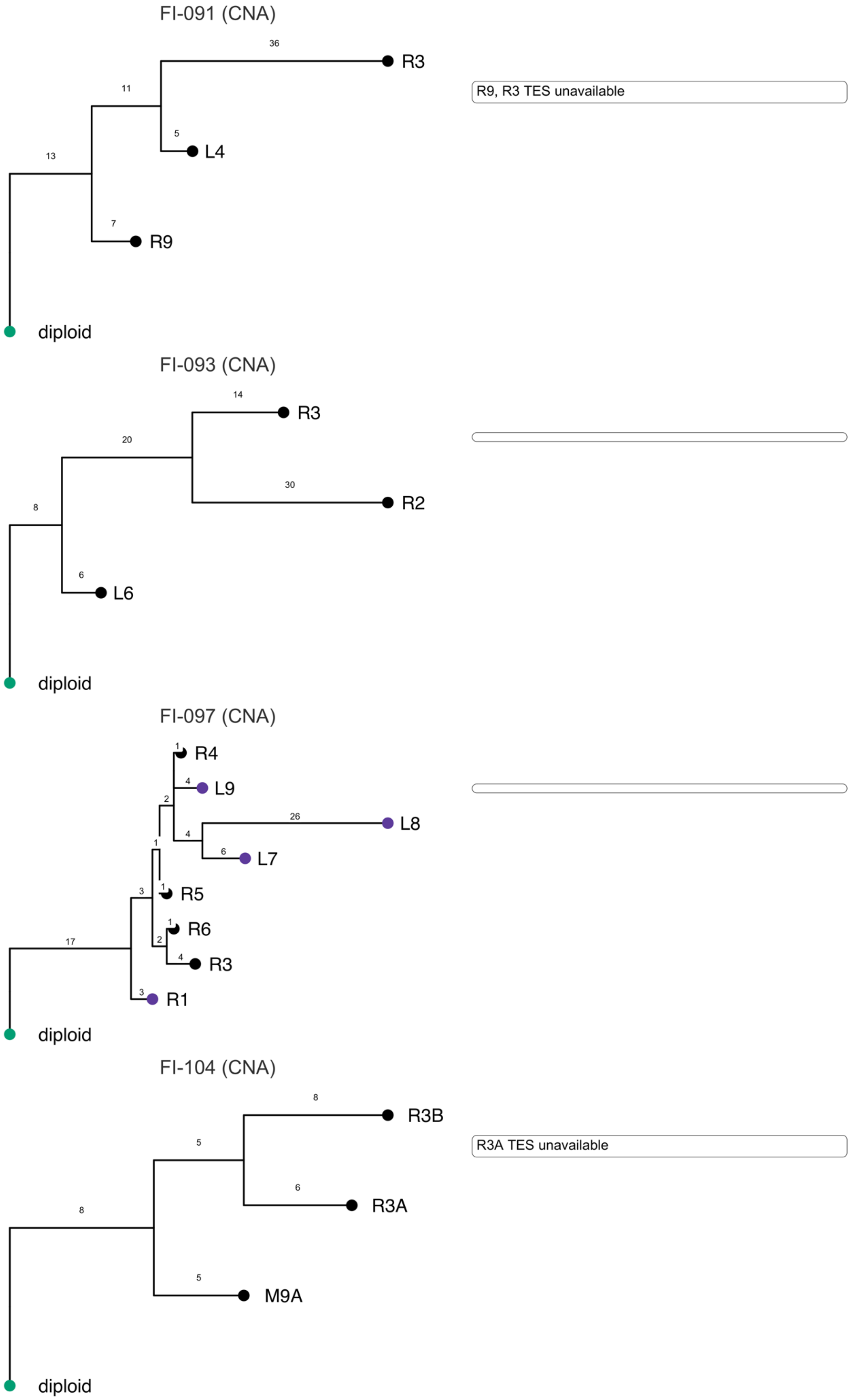

Supplementary Note Figure 1 continued on next page.

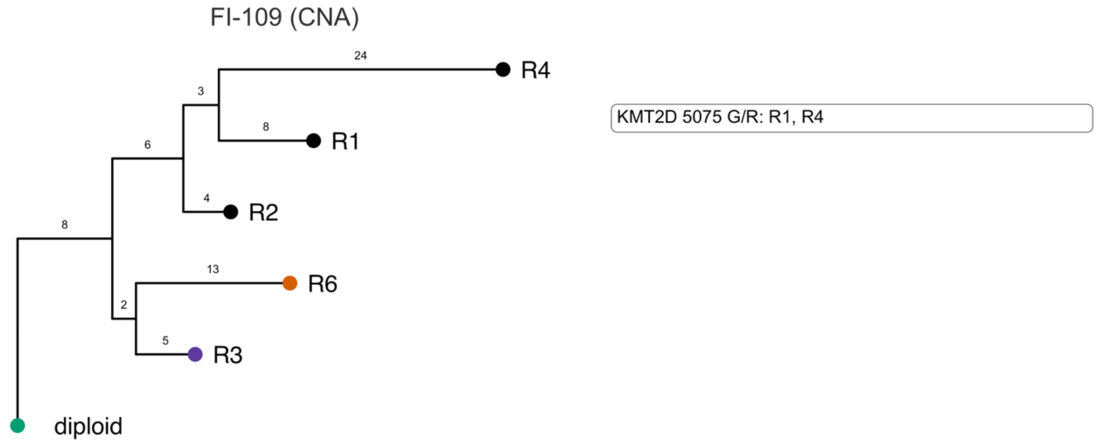

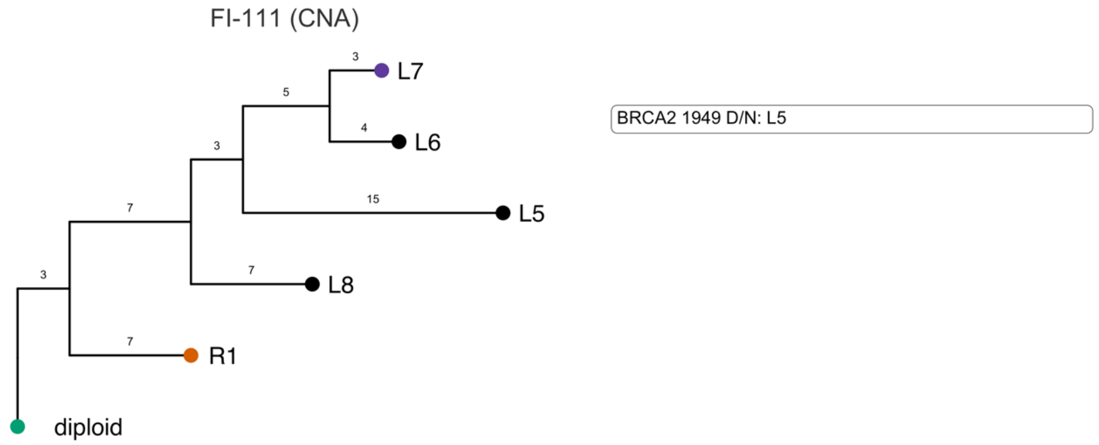

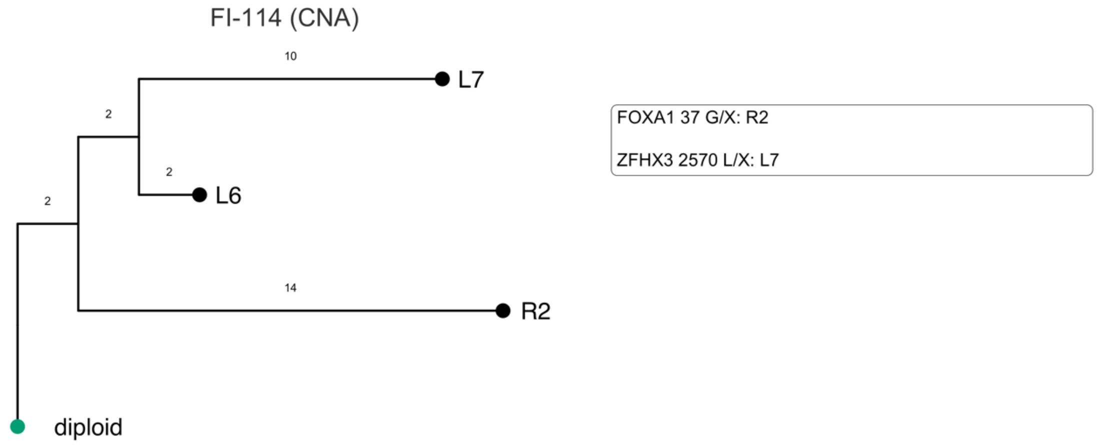

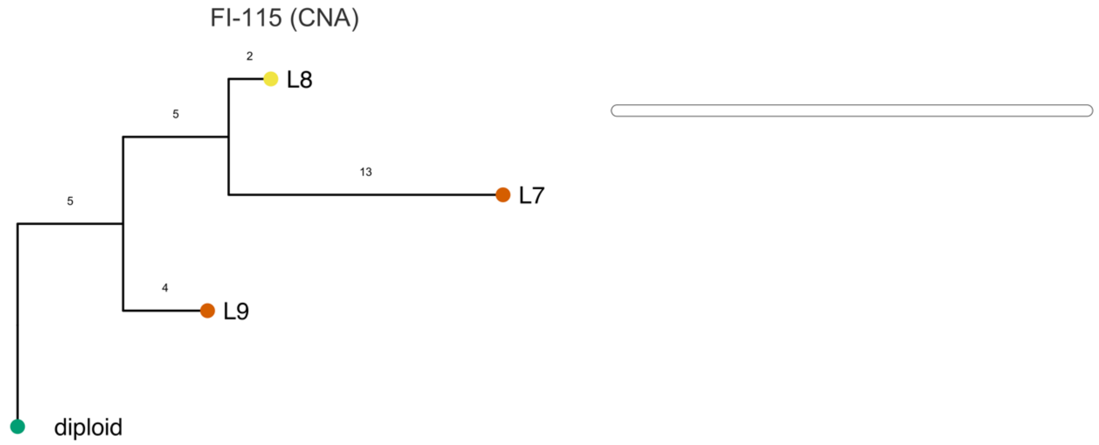

Supplementary Note Figure 1 continued on next page.

FI-118 (CNA)

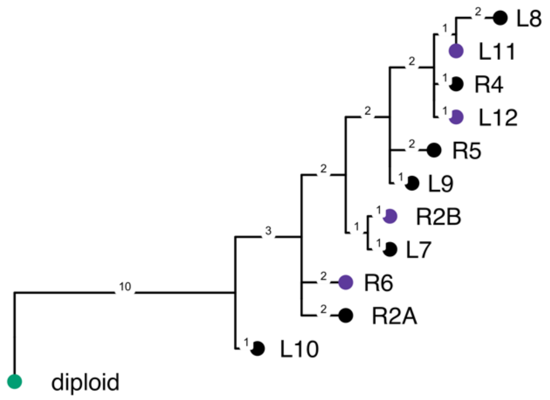

L12, R4, L11, L8, L9, R5, L7, R2B, R2A, R6, L10 TES unavailable

FI-119 (CNA)

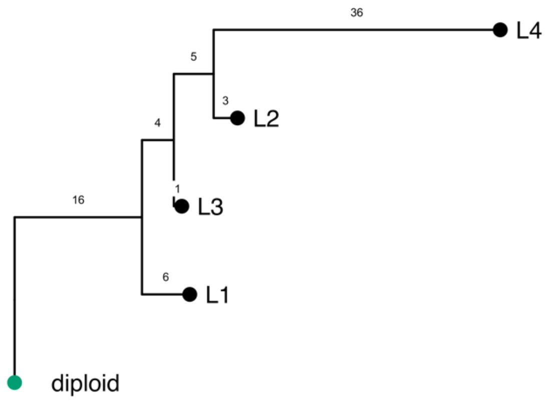

TP53 326 E/*: L1, L2, L3, L4

FI-121 (CNA)

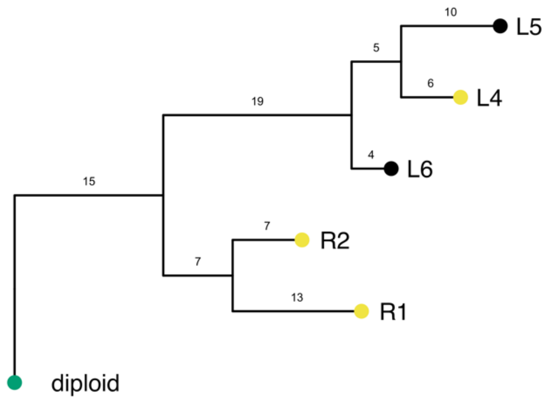

KMT2C 3398 R/Q: L6

TP53 163 Y/H: L4, L5, L6, R1, R2

TP53 73 V/M: L6, R1, R2

FI-122 (CNA)

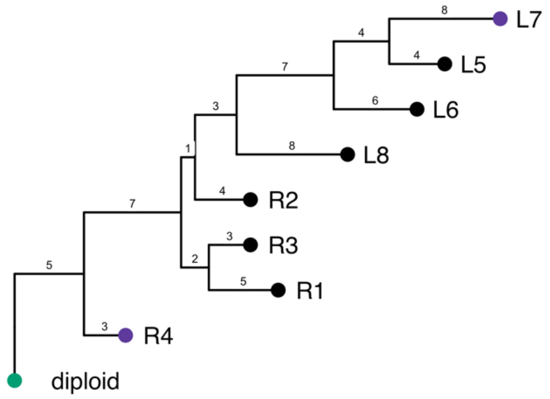

IDH1 129 I/M: L5, L6, L8, R2

ZFHX3 2733 R/C: R3

Supplementary Note Figure 1 continued on next page.

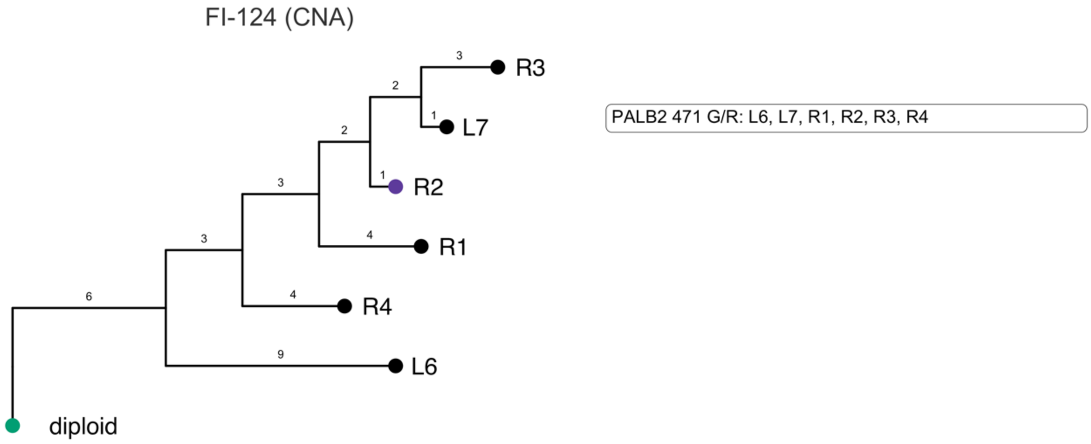

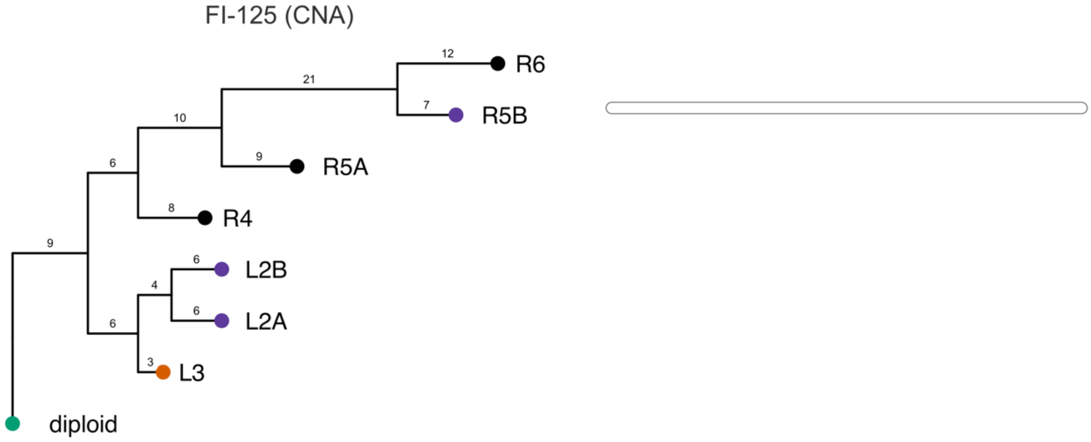

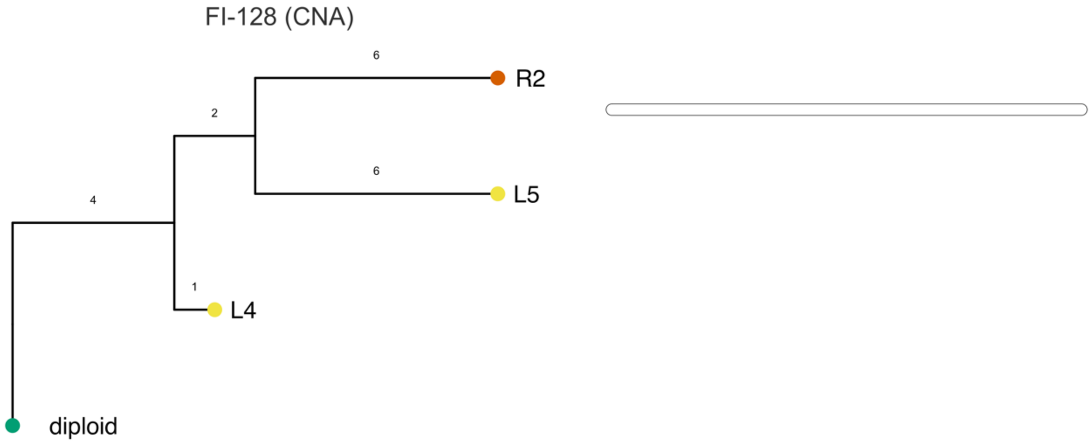

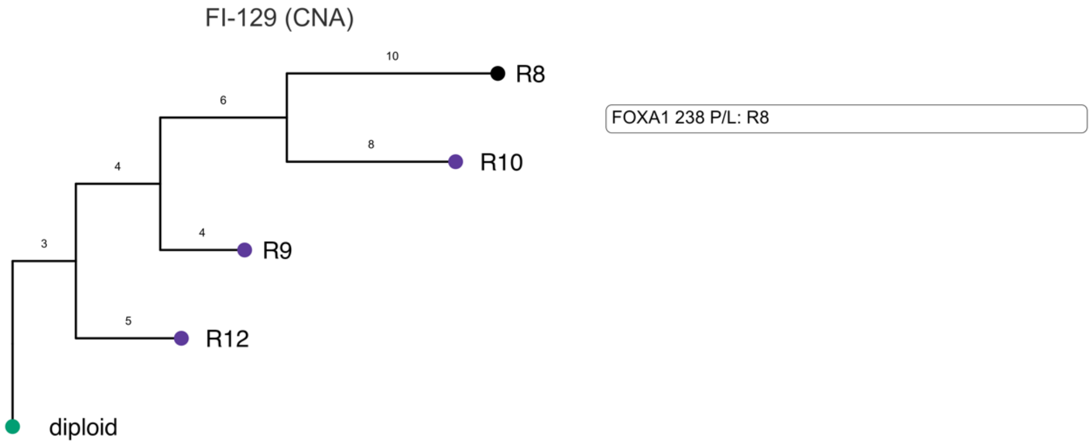

Supplementary Note Figure 1 continued on next page.

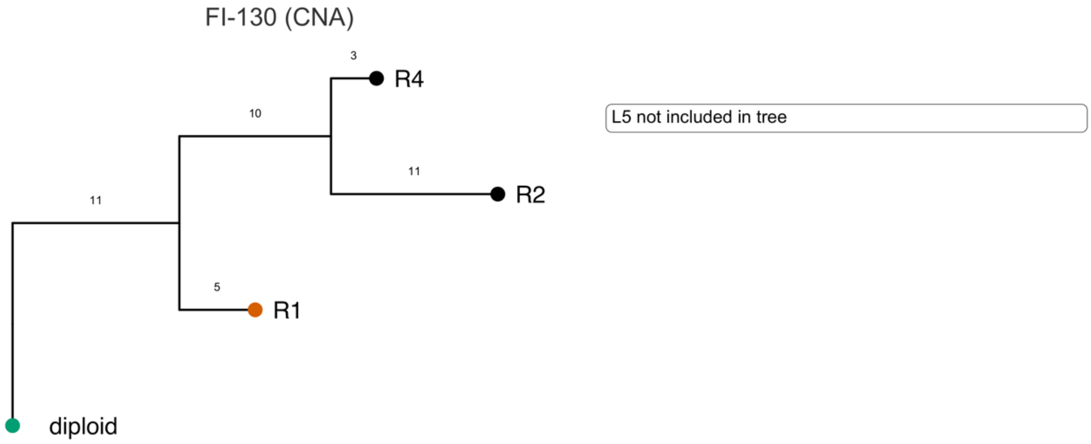

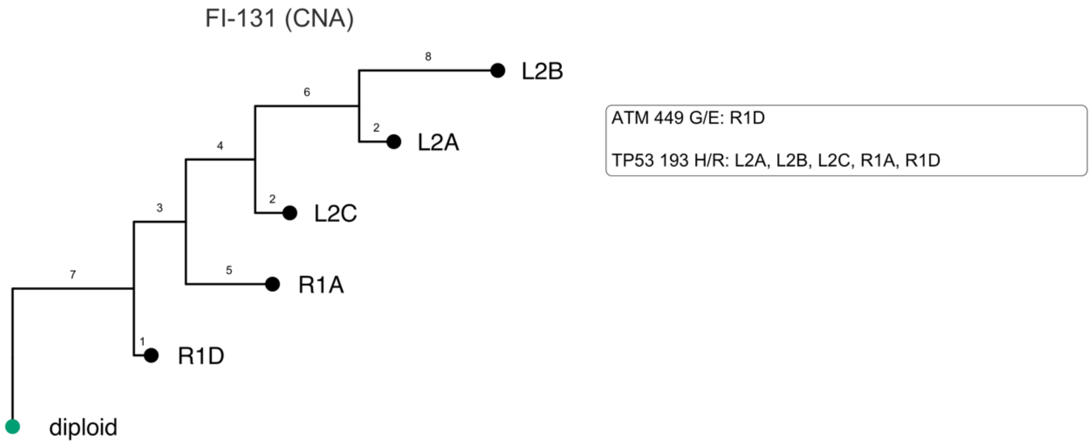

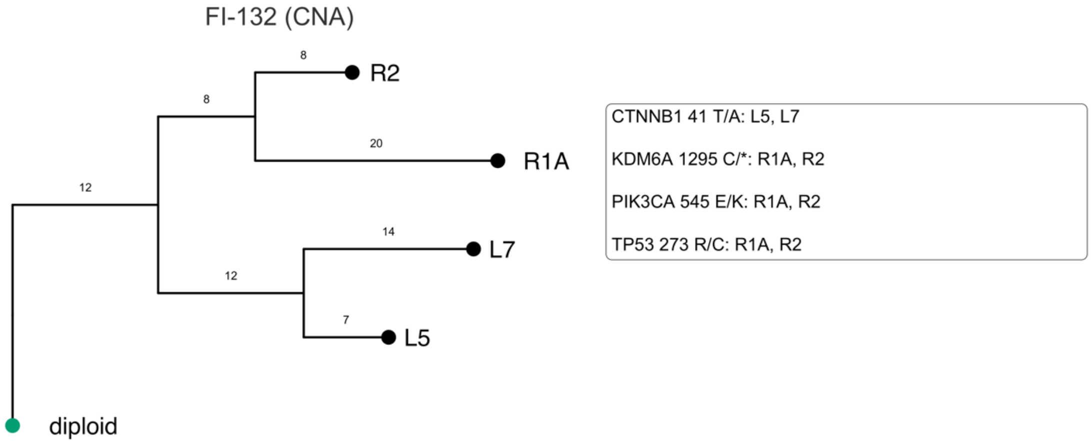

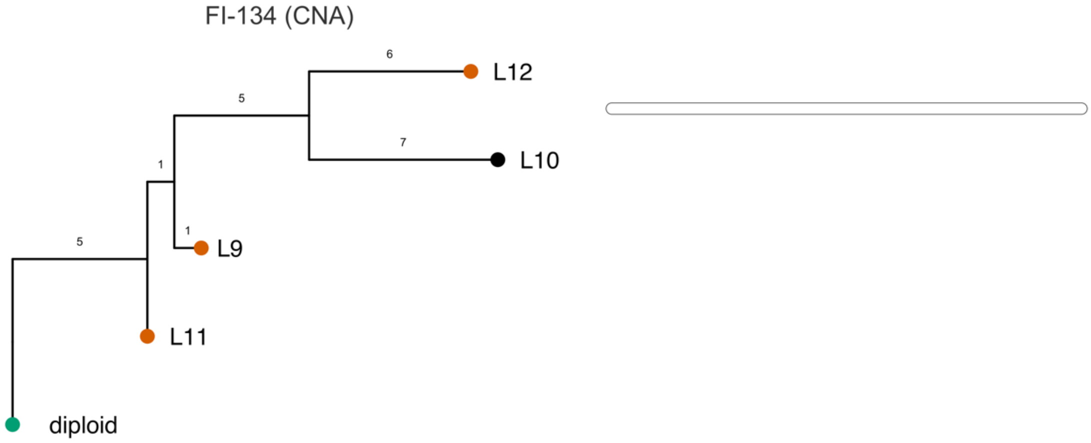

Supplementary Note Figure 1 continued on next page.

FI-137 (CNA)

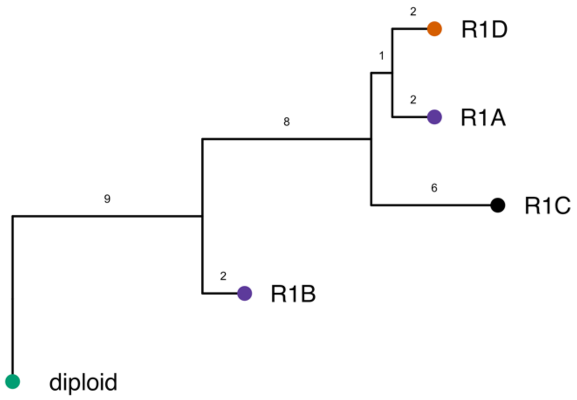

FI-140 (CNA)

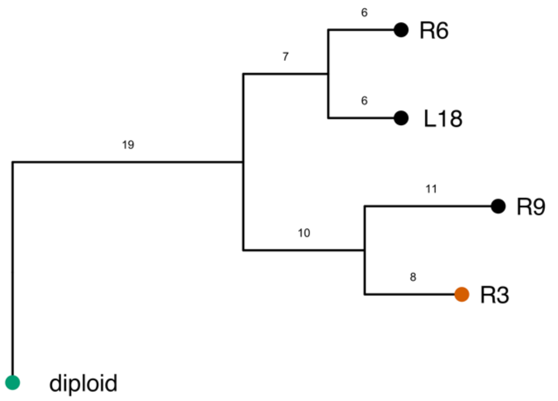

FI-141 (CNA)

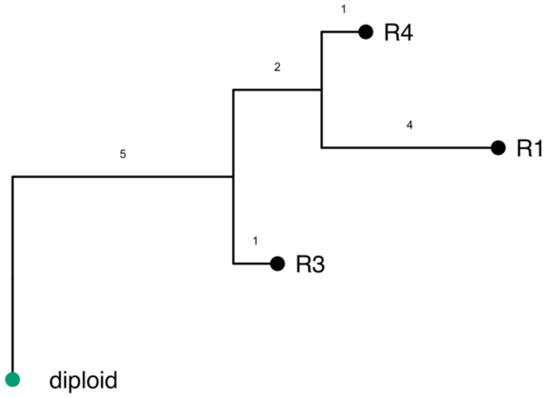

CTNNB1 41 T/A: R1, R3, R4

IDH1 132 R/C: R1, R3, R4

FI-143 (CNA)

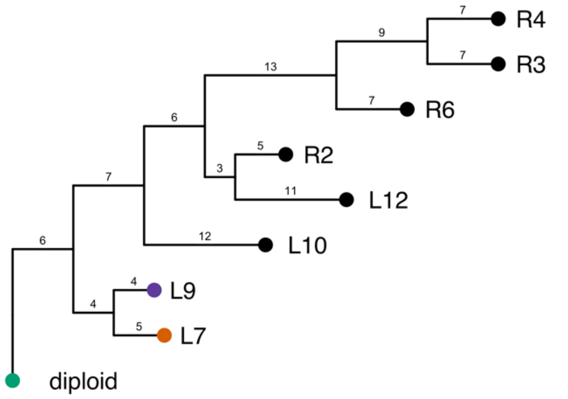

AKT1 79 Q/K: L12, R2, R3, R4, R6

ARID1B 409 P/A: L12, R3, R6

FOXA1 328 P/X: L10, L12, R2, R3, R4, R6

KMT2D 3480 Q/H: R3, R4, R6

TP53 248 R/W: R3, R4, R6

Supplementary Note Figure 1 continued on next page.

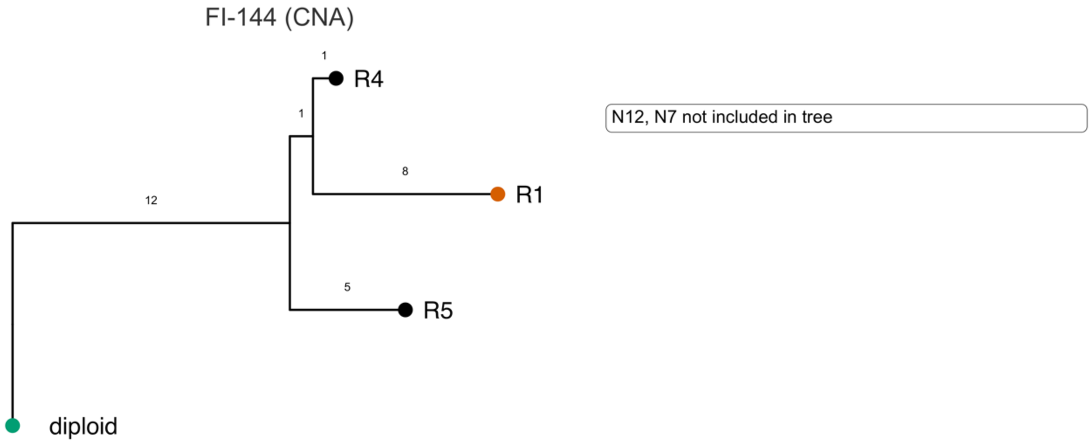

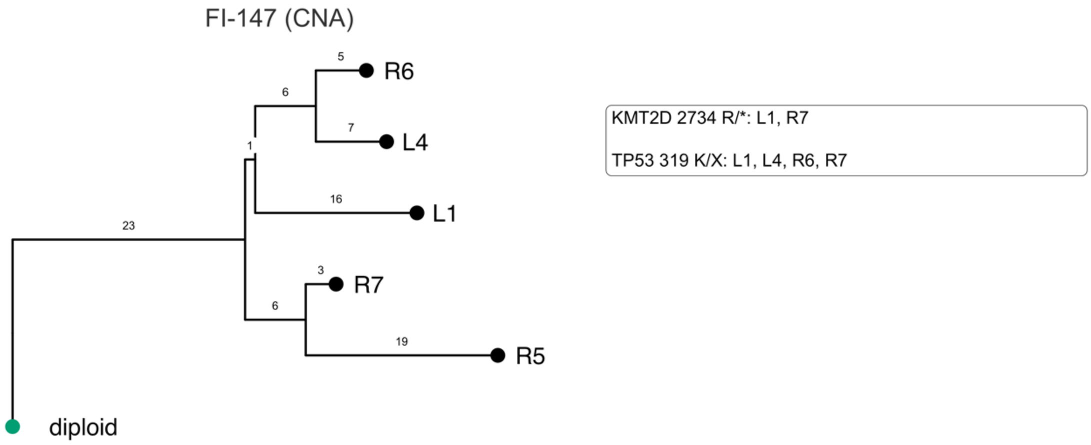

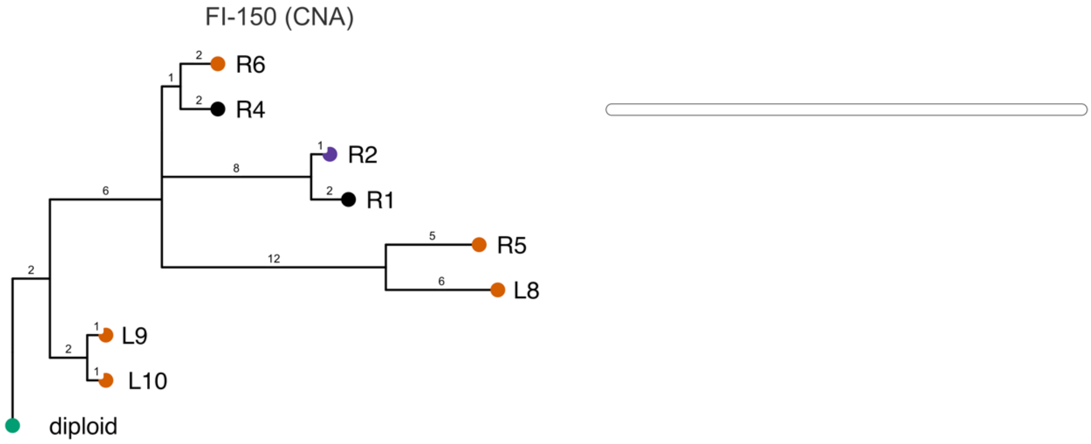

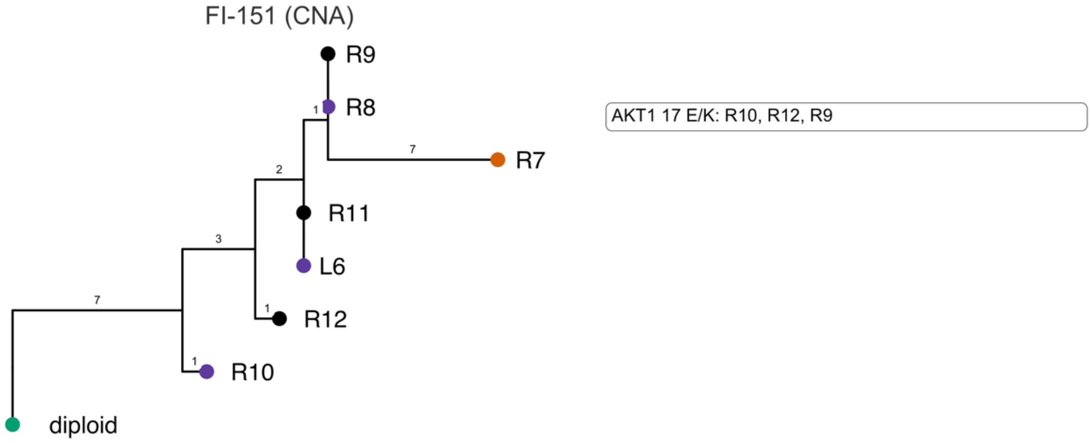

Supplementary Note Figure 1 continued on next page.

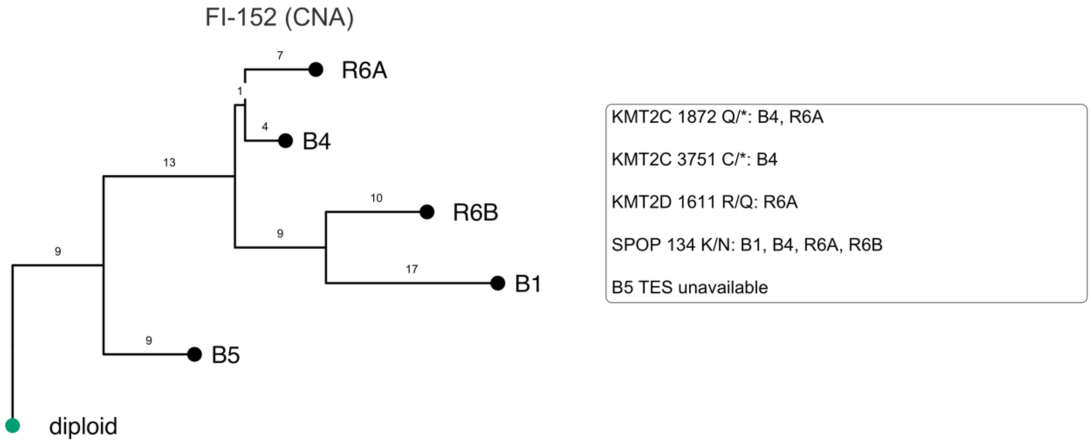

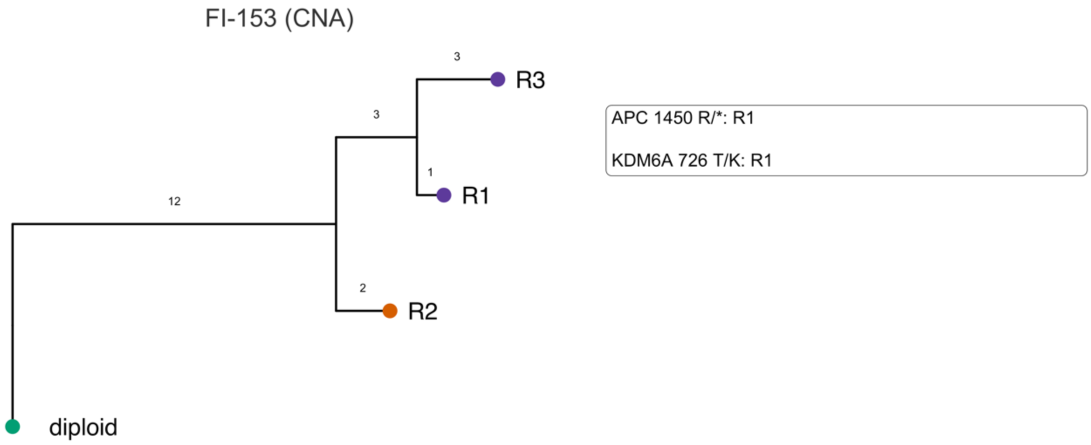

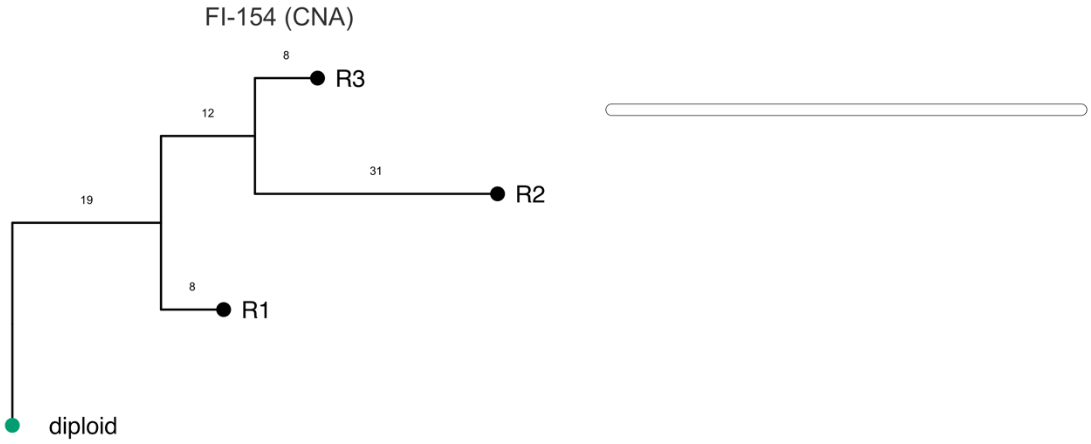

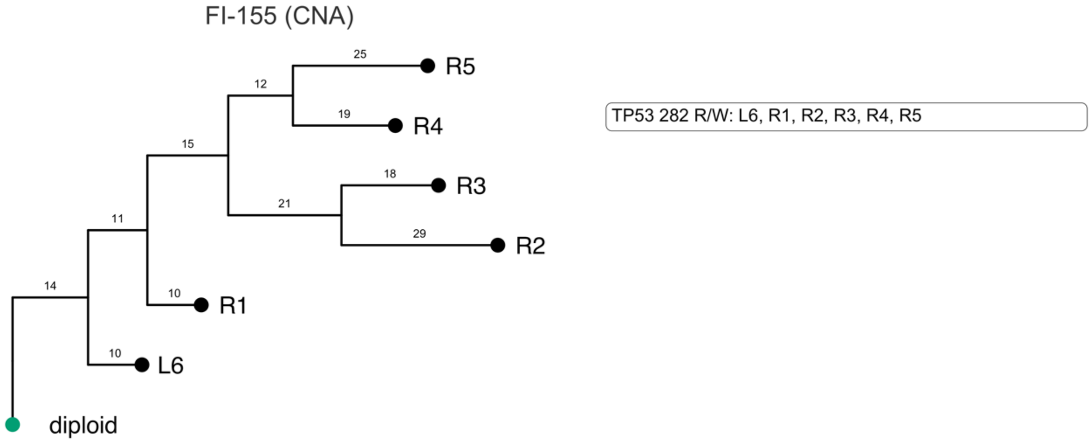

Supplementary Note Figure 1 continued on next page.

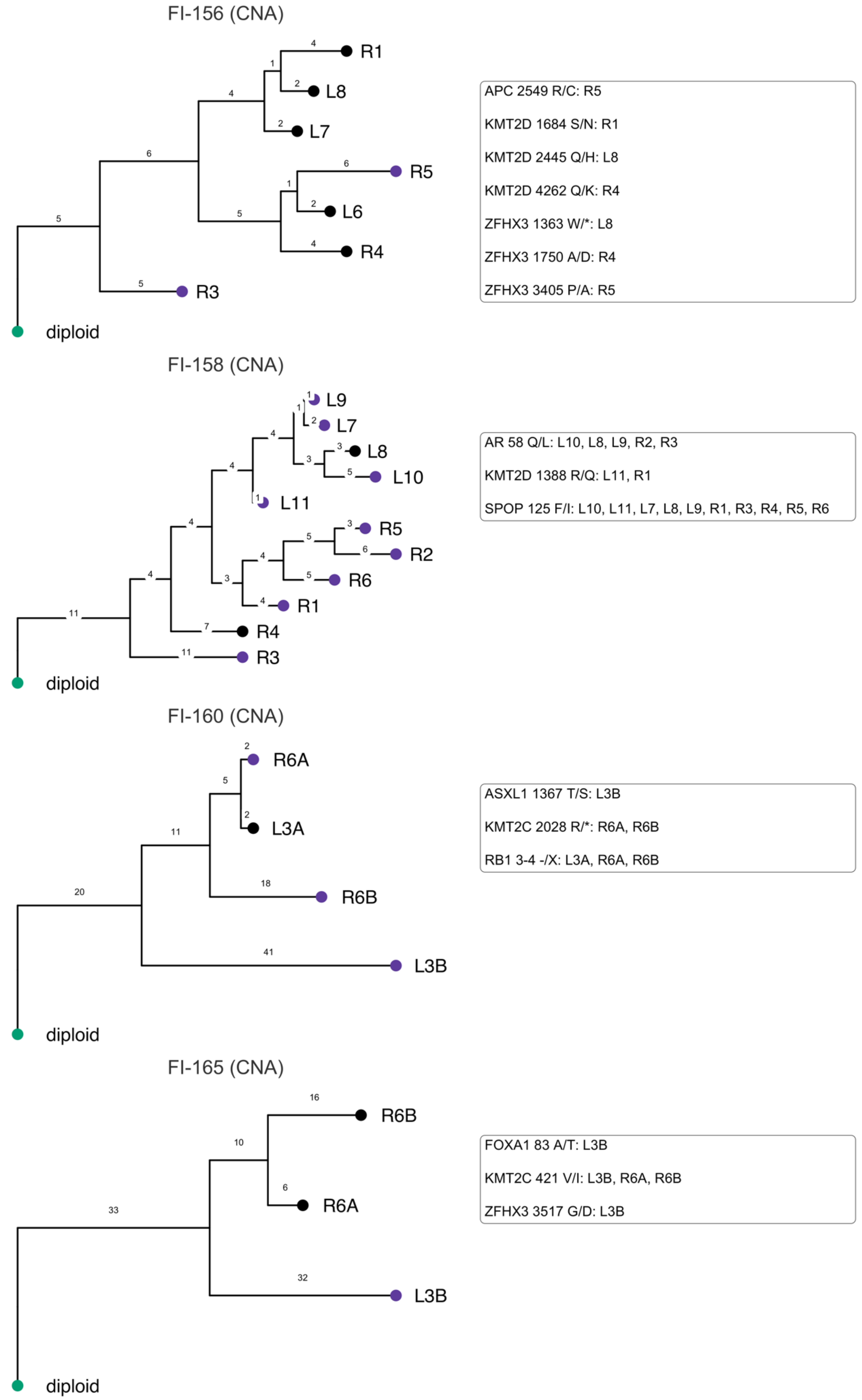

Supplementary Note Figure 1 continued on next page.

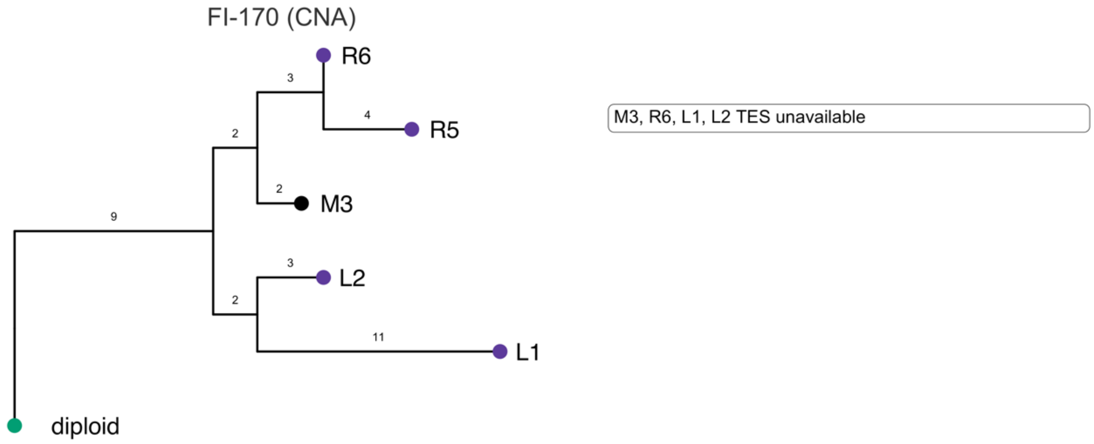

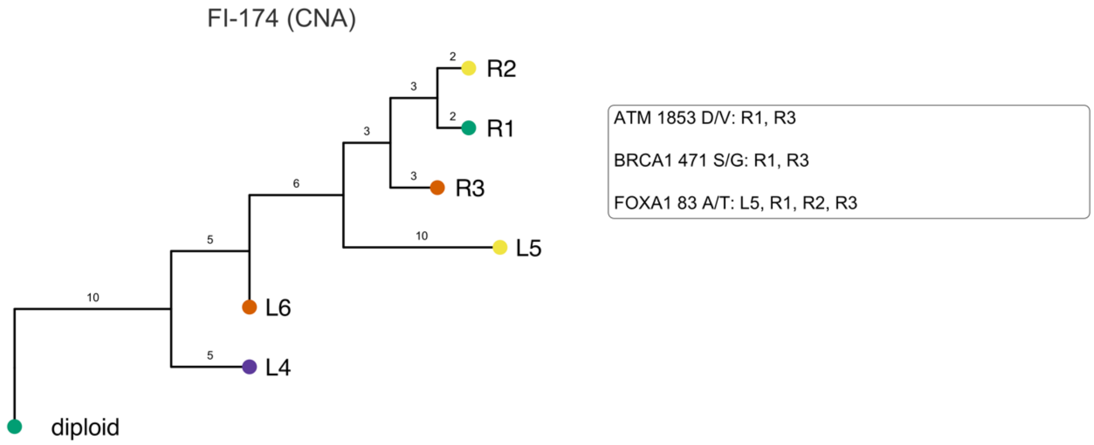

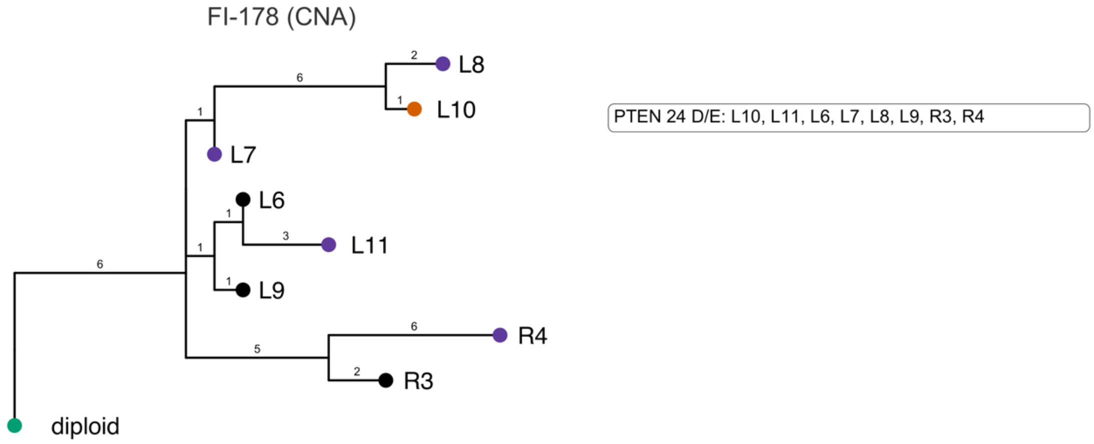

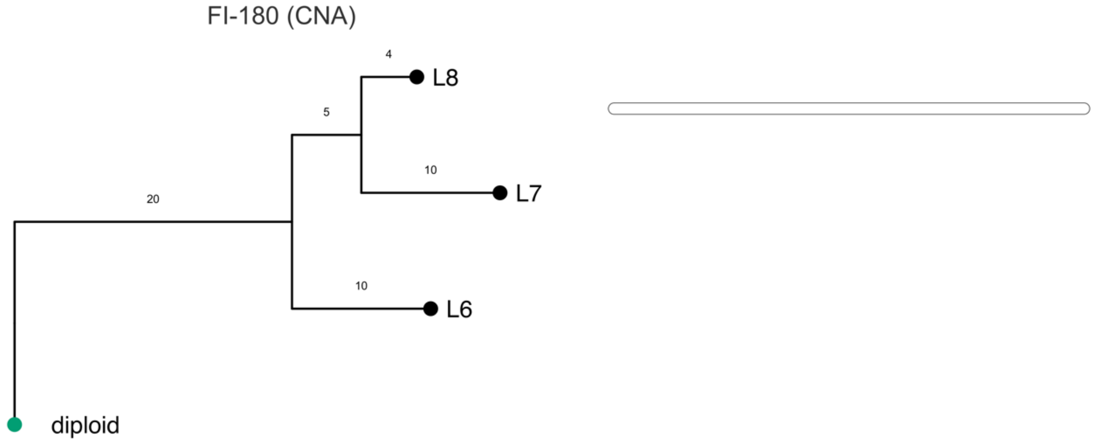

Supplementary Note Figure 1 continued on next page.

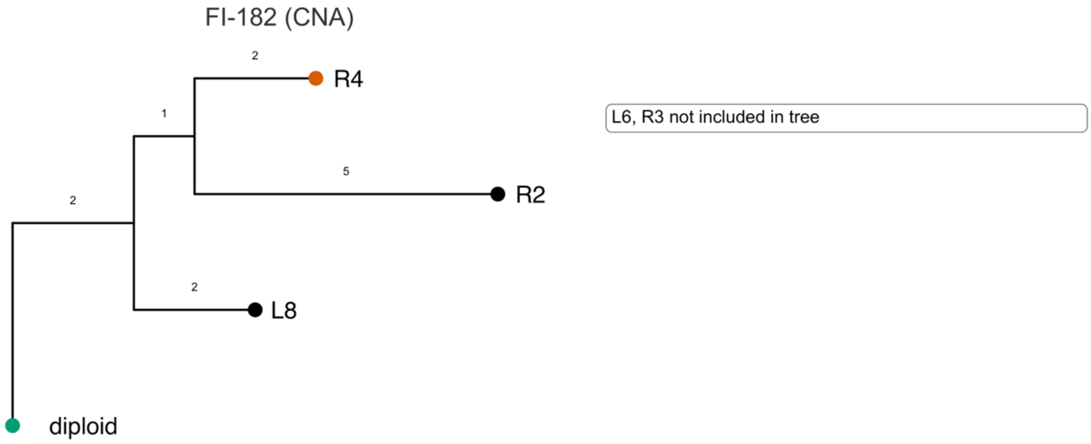

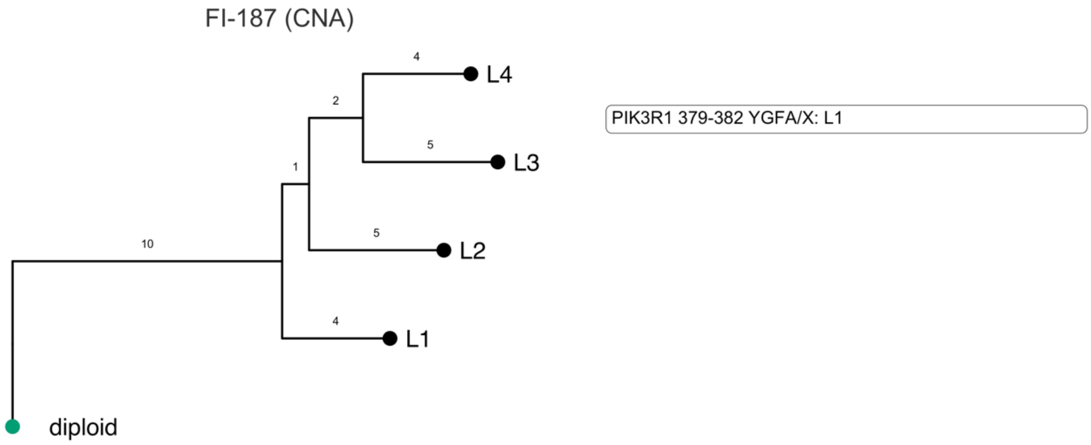

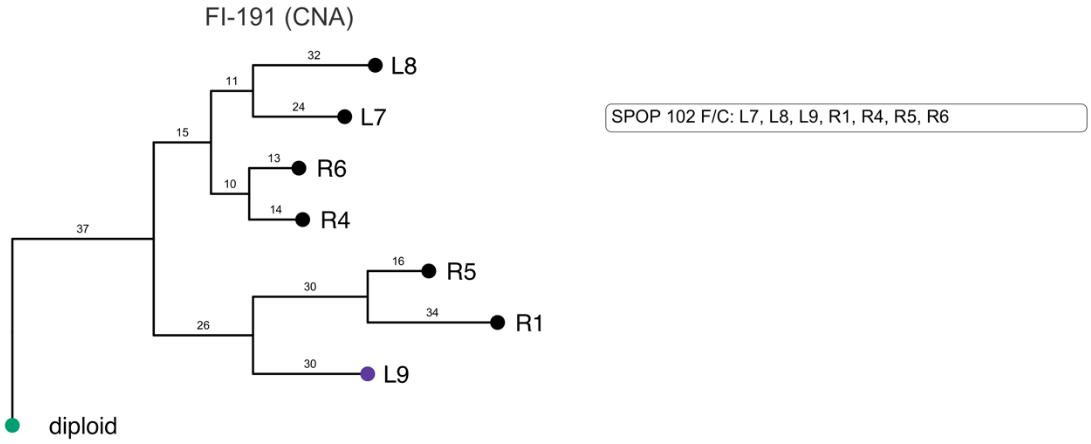

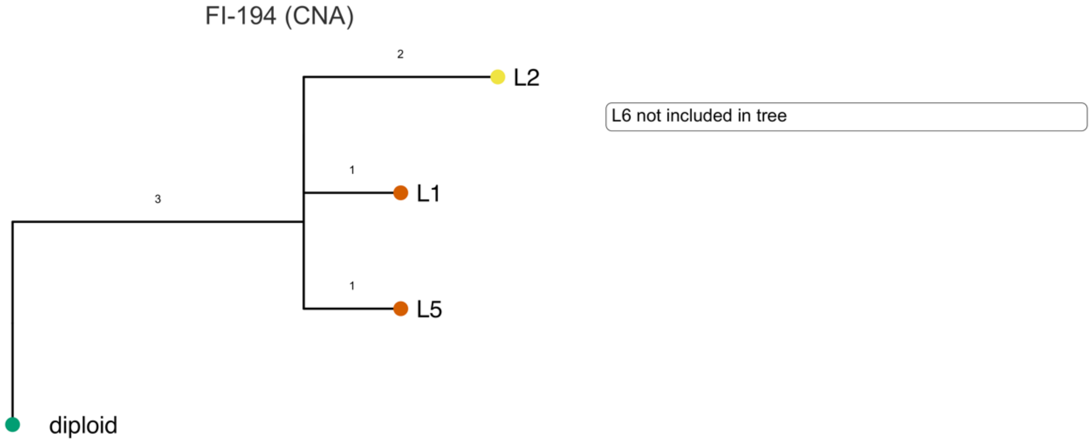

Supplementary Note Figure 1 continued on next page.

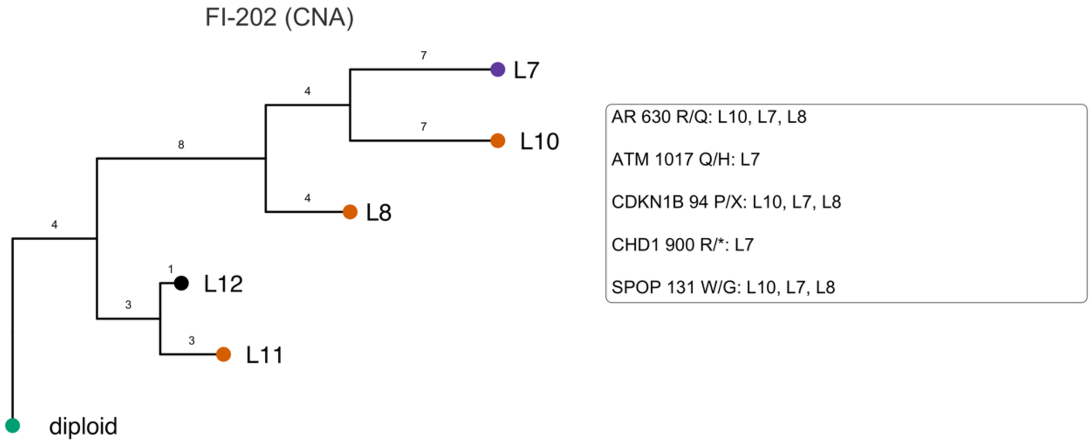

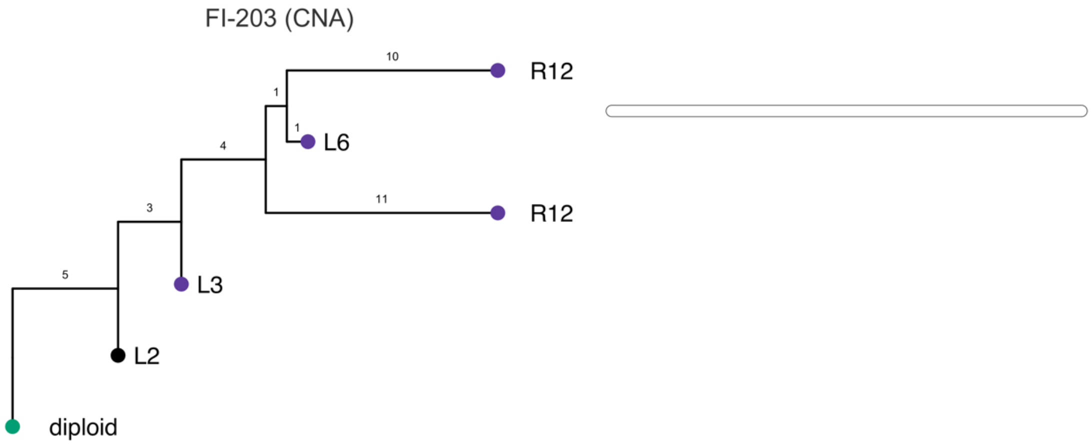

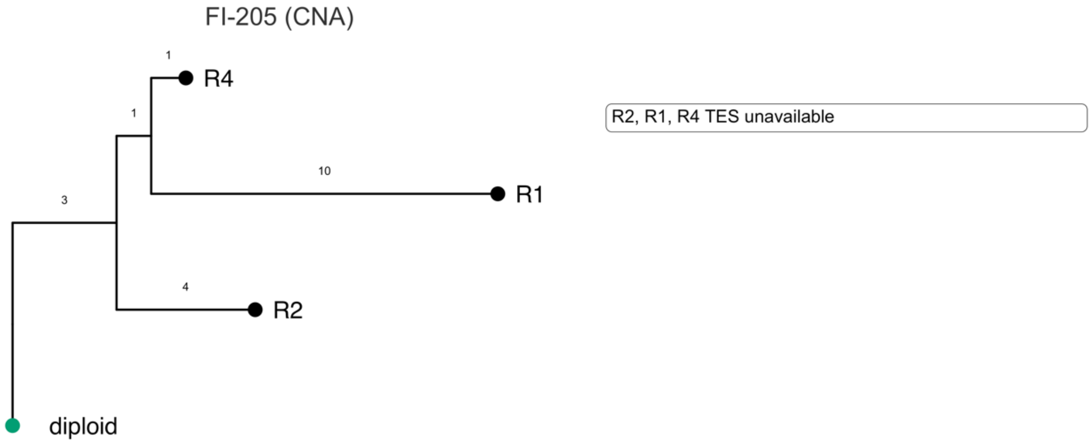

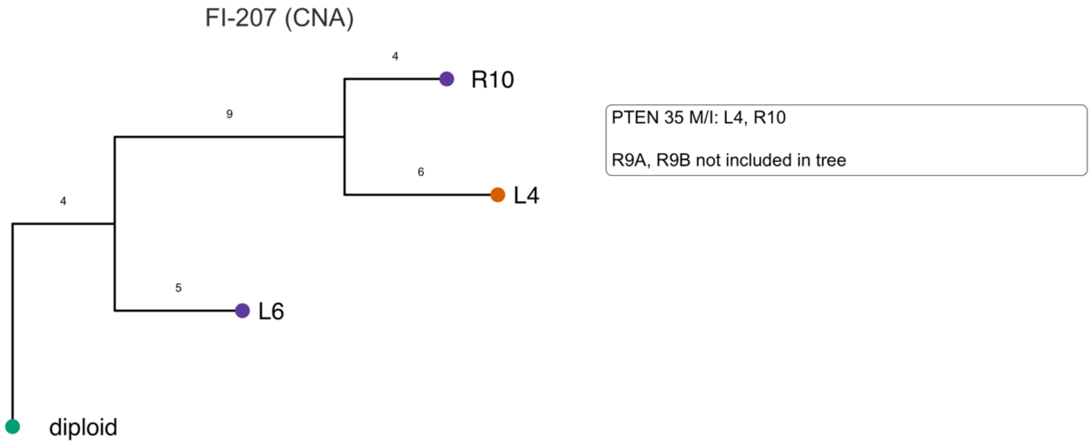

Supplementary Note Figure 1 continued on next page.

FI-208 (CNA)

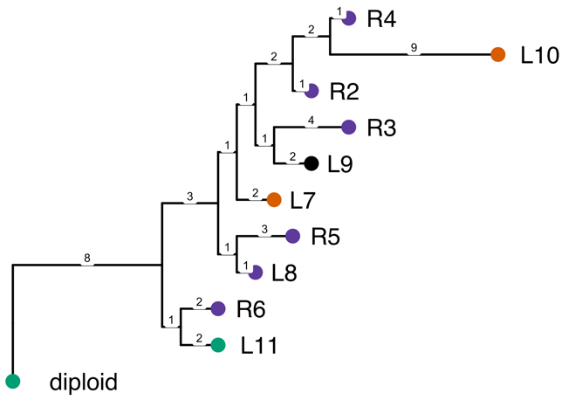

FI-210 (CNA)

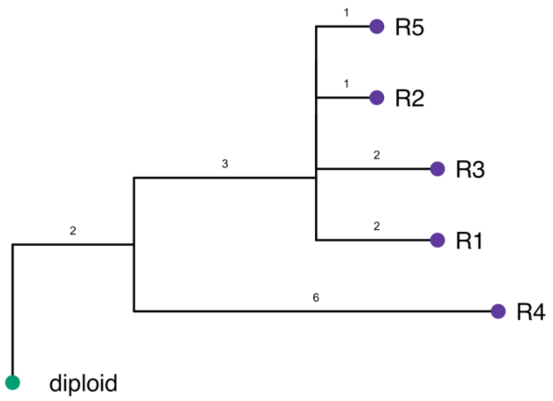

ARID1A 1671 R/W: R5

CDK12 1242 R/*: R1, R2, R3, R4, R5

KMT2D 3547 R/C: R1, R2

TP53 248 R/Q: R1, R2, R3, R4, R5

FI-212 (CNA)

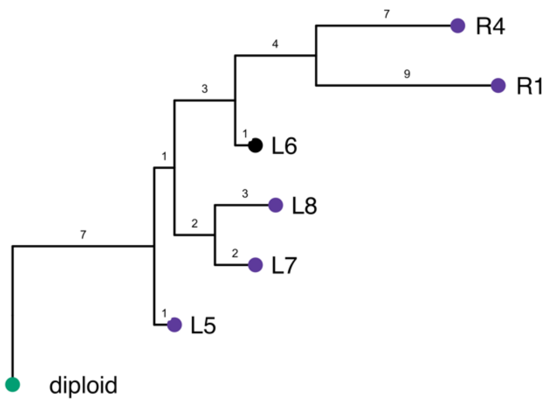

APC 1447 Q/QX: L5, L7

APC 635 G/*: L8

FI-213 (CNA)

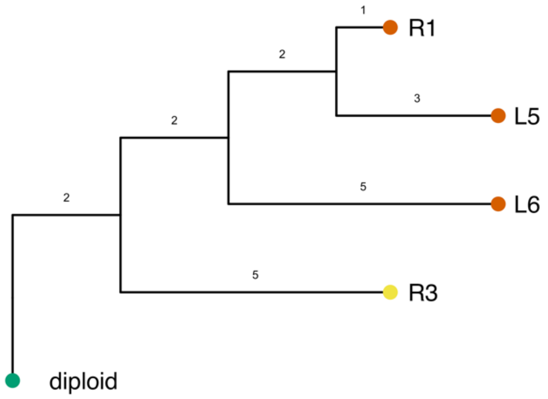

L8 not included in tree

Supplementary Note Figure 1 continued on next page.

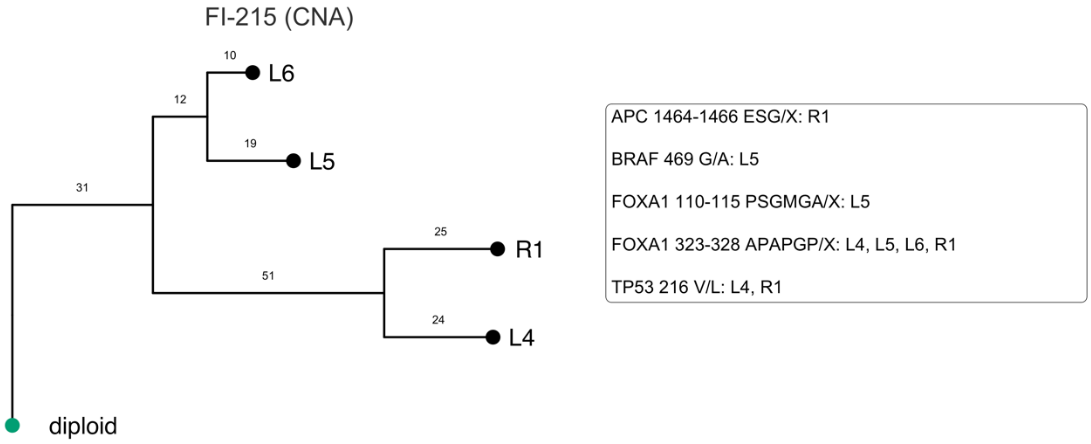

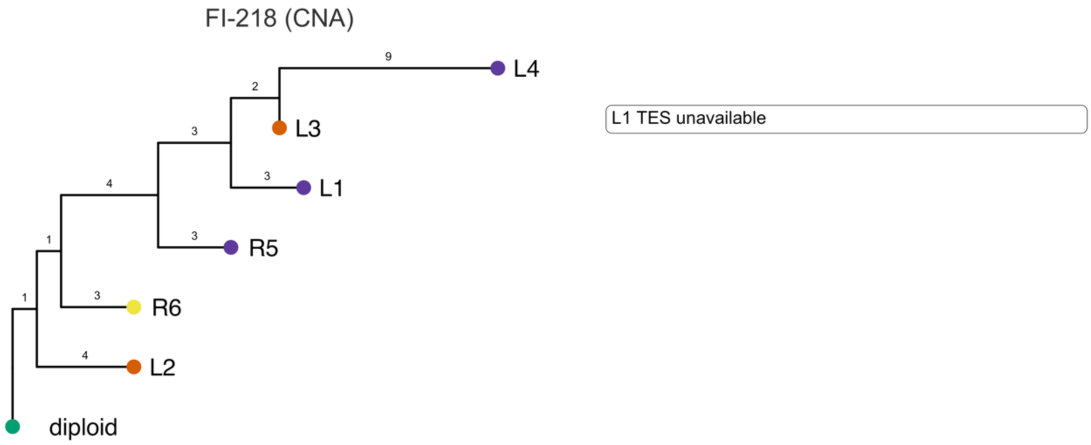

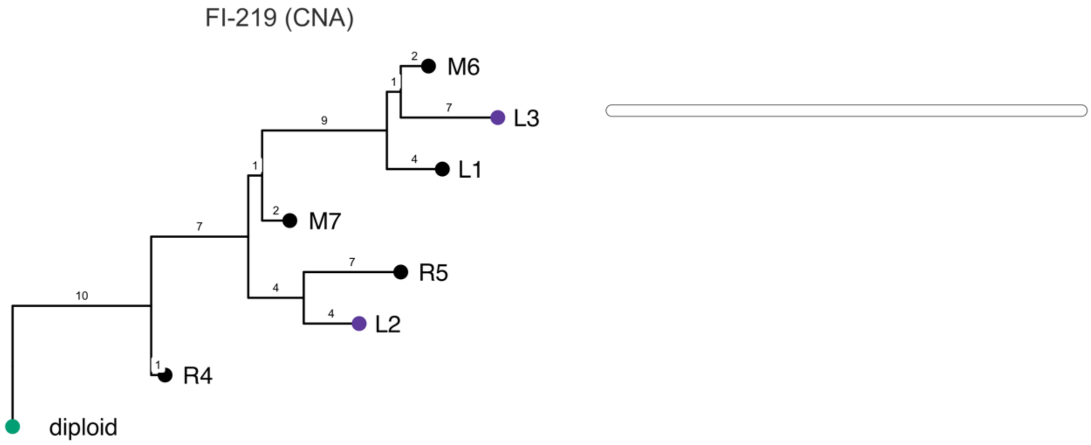

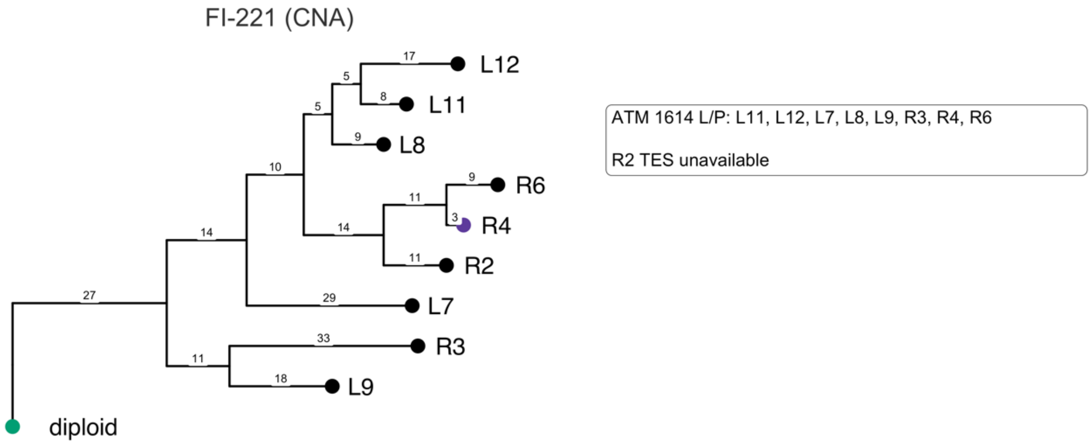

Supplementary Note Figure 1 continued on next page.

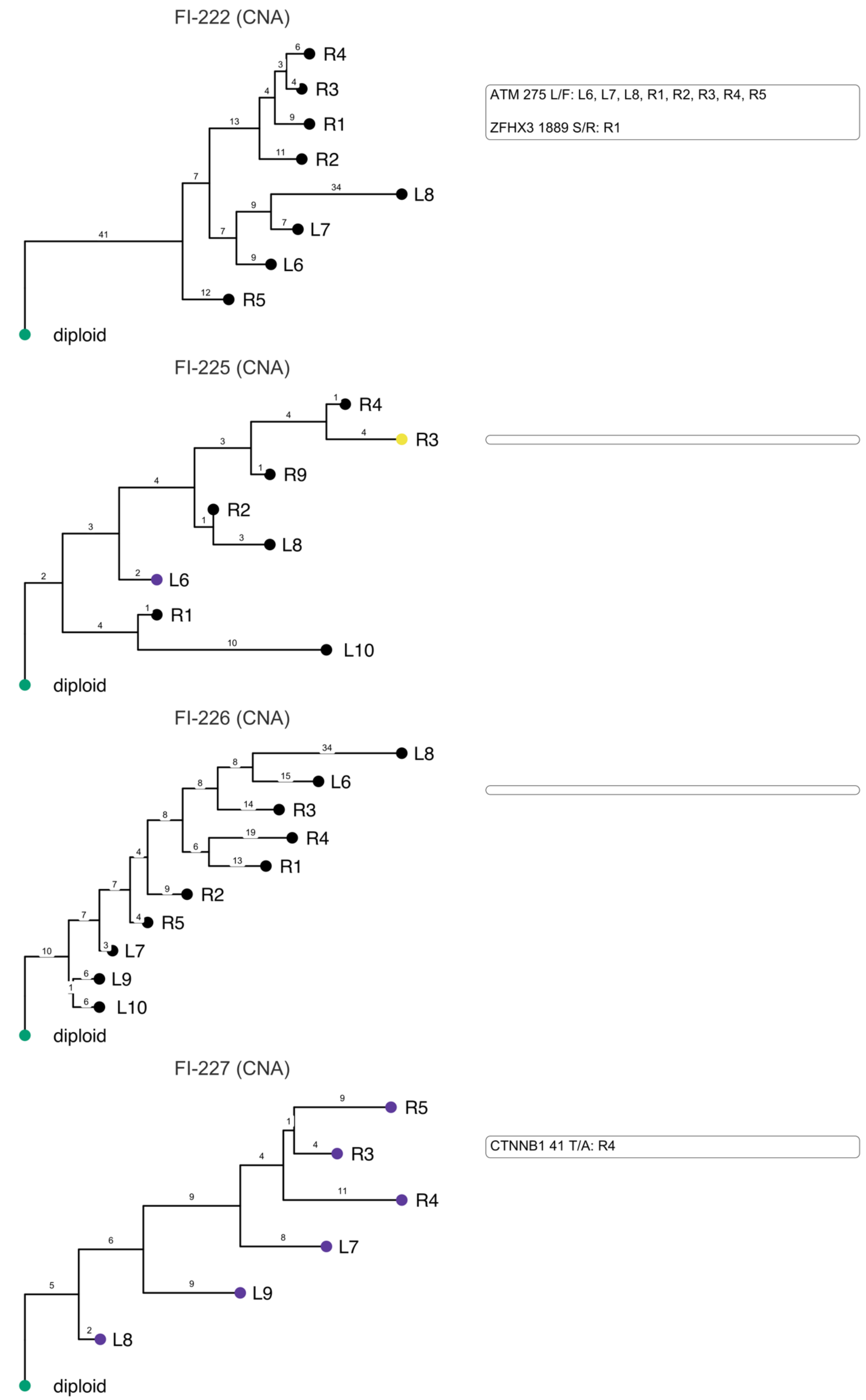

Supplementary Note Figure 1 continued on next page.

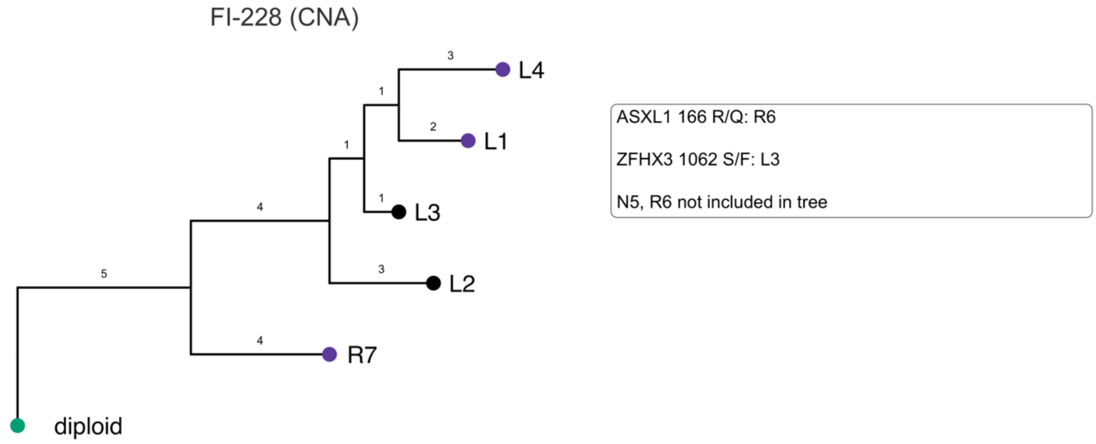

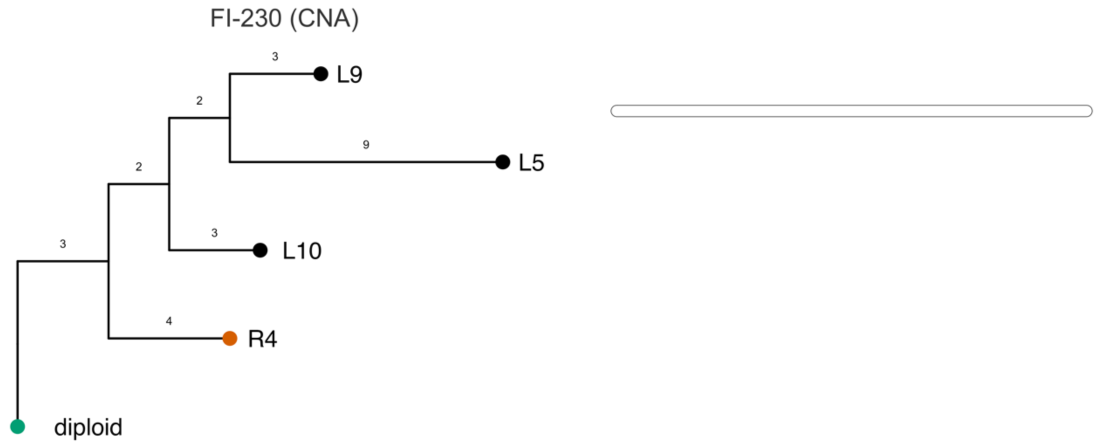

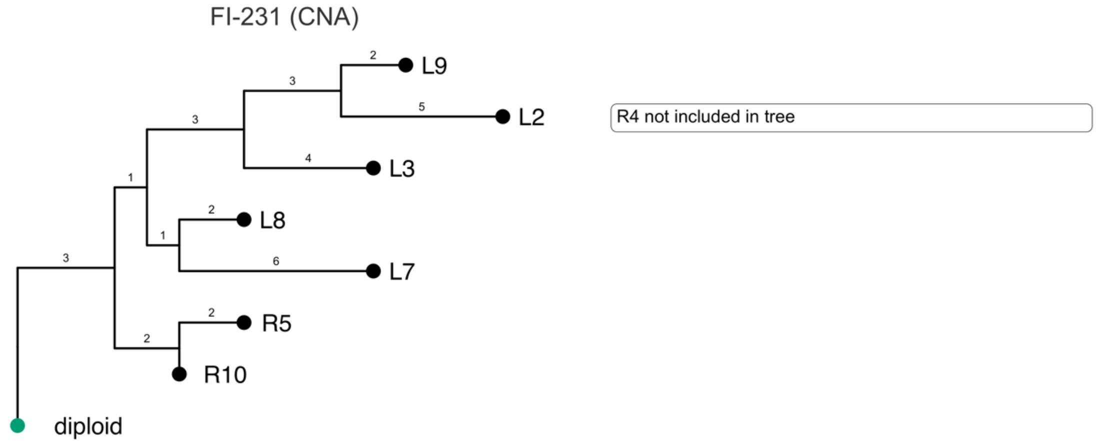

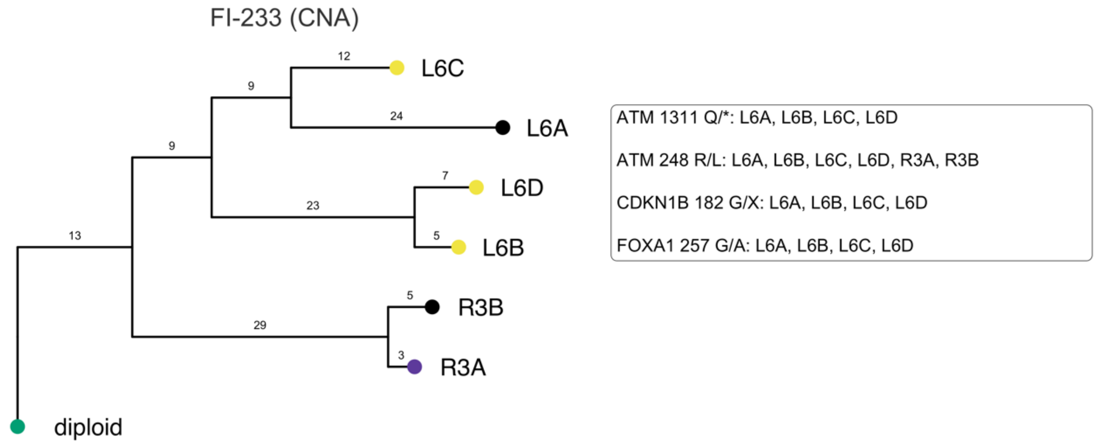

Supplementary Note Figure 1 continued on next page.

FI-234 (CNA)

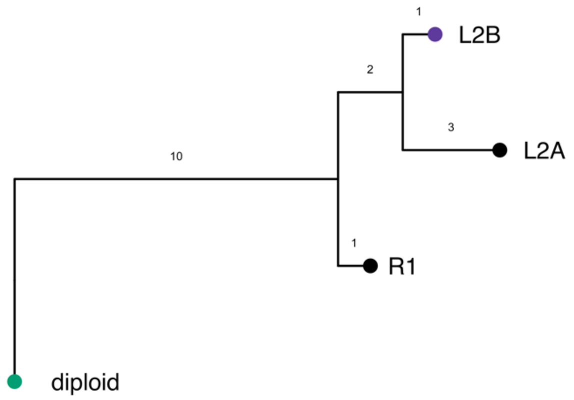

R1, L2A, L2B TES unavailable

FI-242 (CNA)

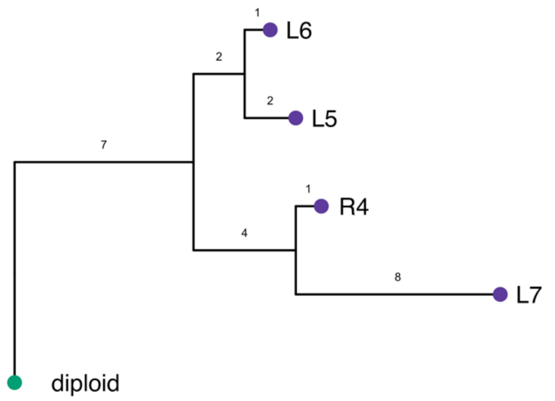

FI-243 (CNA)

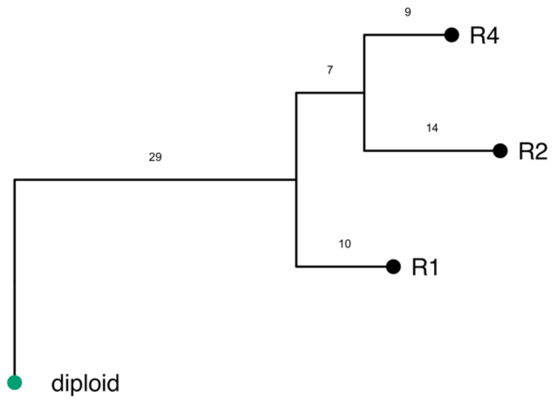

FI-244 (CNA)

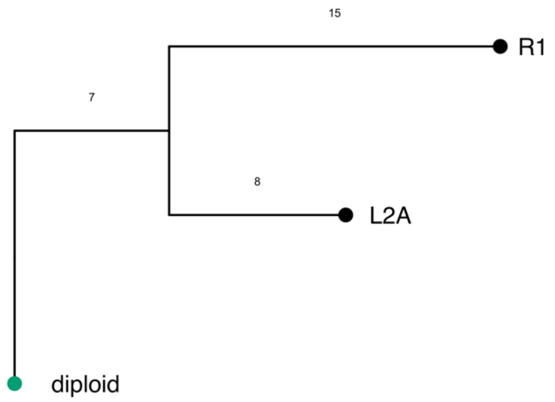

TP53 198 E/*: L2A, R1

B2 not included in tree

Supplementary Note Figure 1 continued on next page.

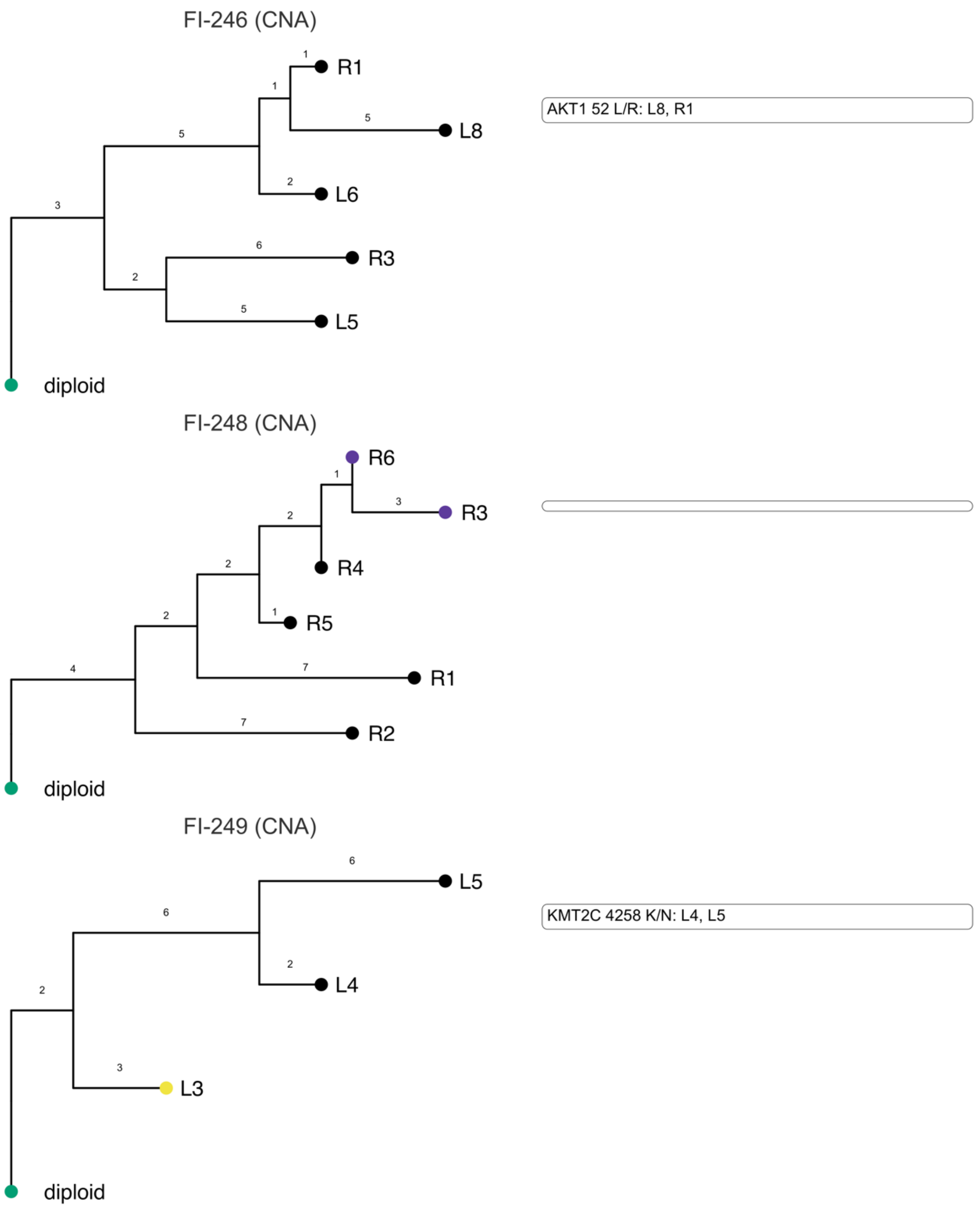

Supplementary Note Figure 1. Copy number phylogenetic trees. Per patient copy number phylogenetic trees computed with MEDICC2, with annotated mutational profiles per sample. Nodes are coloured by Gleason grade as determined by the automatic classifier. Nodes are annotated as following; diploid/normal (green), Grade group 2 (yellow), Grade group 3 (orange), Grade group 4 (purple), Grade group 5 (black), no data (grey). Samples with no targeted exome sequencing (TES) data available are labelled as “TES unavailable”. Samples not included in the phylogenetic analysis (e.g. PGA < 0.01) are also noted as “not included in the tree”. If box is empty, no mutations were called but data was available for all samples and all samples were included in the tree.

Computational Analysis Supplementary Note

N.B. We refer readers to the main manuscript for tool references.

Bioinformatics

Low-pass WGS copy number calling

To call absolute copy number we used the ASCAT approach however, as we only had log2 ratio values available due to the low coverage of the data, we sought to leverage multiple sampling to search for ploidy solutions. We firstly calculated median value of the Euclidean distances from zero for all bins in each sample. If the maximum observed value was greater than 0.003 for the set of samples across a patient, we searched a ploidy range of 3.5 to 4.5, otherwise we searched a range of 1.5 to 2.5. We used a minimum purity of 0.1 for all samples. We recorded the per sample fit for each ploidy value using autosomes only. We then recorded the pairwise Euclidean distances of the integer copy number values of each sample divided by the ploidy, filtering for bins with a log2 ratio greater than 1 to avoid distances being exaggerated by amplifications. To determine the ploidy of the patient we compared the mean pairwise Euclidean distances of ploidy normalised copy number values of each sample pair to the mean per sample fit for each ploidy value after linearly scaling each measure to the maximum and minimum values, (i.e., minimum equals 0, maximum equal 1). Ploidy solutions were then ranked according to their Euclidean distance from both values being zero. This was performed to ensure the chosen ploidy value produces a good fit both within samples and across samples. Using the chosen purity and ploidy per sample we then calculated the X and Y copy number considering the normal copy number of 1 for both chromosomes in male genomes. For samples in which no fit was produced for the patient ploidy, a default purity of 40% was chosen. A selection of bins was blacklisted from the ploidy search as they were seen to be recurrently aberrant. Proportion of the genome altered (PGA) was measured by calculating the fraction of bins not at the rounded baseline ploidy (this was expected to be half at sex chromosomes).

Unique molecular identifier processing parameters (FFPE targeted panel)

Unique molecular indexes (UMIs) were processed using the fgbio pipeline (http://fulcrumgenomics.github.io/fgbio/). Briefly, FASTQ files are converted to unaligned BAM files and Illumina adaptors are marked and the BAM file is then converted back to a single FASTQ using Picard tools. An initial alignment is performed using bwa mem as previously but with a minimum seed length of 50 (-k) and a maximum of one maximal exact matches (-c), marking shorter split hits as secondary (-M). The SAM file output of the alignment is then merged with the unaligned BAM file using Picard tools. Reads are then grouped by their UMI using fgbio and the adjacency method (-s), minimum mapping quality of 30 (-m) and a minimum UMI length of 9. The output is then sorted and consensus reads are called using *callMolecularConsensusReads* with the option of a minimum base quality of 30 (--min-input-base-quality) in fgbio. Consensus reads are then filtered (*FilterConsensusReads*) with a minimum number of reads required to support a base of 2 (--min-reads), a maximum read error rate of 0.05 (--max-read-error-rate), a minimum base quality to generate a non-N read of 30 (--min-base-quality), a maximum allowed fraction of no calls of 0.1 (--max-no-call-fraction) and the option of reverse per base tags on reverse strand reads (--reverse-per-base-tags), then sorted using Picard tools. Consensus reads are subsequently converted to FASTQ files and aligned using bwa mem as previous and sorted using Picard tools. The pre- and post-alignment BAM files are then merged, and duplicates are marked using Picard tools. The file is indexed using samtools.

Additional somatic mutation calling filtering post-deepSNV

To flag high quality single nucleotide variants we separately called mutations using deepSNV. Normal tissue targeted sequencing BAM files post-UMI processing and SSARs filtering were used as the normal reference panel (n=11). A minimum depth of 30 was used as well as a minimum base quality of 25 and no minimum mapping quality for loading BAM files to generate counts.

Only single nucleotide variants that were also detected by deepSNV, had a minimum quality score (QUAL) according to platypus of 10, at least one mutation with a variant allele frequency greater than 0.05 and a mappability greater than 0.6 (as calculated by the GEM library to generate QDNAseq bins and bigWigAverageOverBed, https://hgdownload.cse.ucsc.edu/admin/exe/) were used for analysis. Additionally, due to the presence of unexpected recurrent mutations previously unreported in prostate cancer produced by SSARs, base positions were required to be observed to be mutated in no more than 3 patients in the cohort, in line with known hotspots in the *SPOP* gene. Small insertions and deletions (InDels) were not required to be detected by deepSNV. However other filters were made stricter, the position must be uniquely mappable, we only allowed the detection of an InDel in a single position in the cohort as no recurrence was expected and the minimum quality score (QUAL) was raised to 100. Mutations with multiple alternatives were also removed.

Unique molecular identifier processing parameters (cfDNA whole exome sequencing)

Whole exome sequencing data from cfDNA was analysed using the fgbio pipeline as for the primary tissue samples, however we utilised a NextFlow implementation (https://github.com/chelauk/nf-core-umialign) with minor differences. BWA alignment was performed using standard settings. Default options were used for minimum mapping quality and minimum UMI length in *GroupReadsByUmi*. UMI families were required to contain a minimum of 2 reads (--min-reads = 2, *CallMolecularConsensusReads*). Maximum read error rate was set to default in *FilterConsensusReads* (--max-read-error-rate). Duplicates were marked using *UmiAwareMarkDuplicatesWithMateCigar*. SSARs were not required to be filtered.

Mutations were called similarly to the primary sample targeted sequencing, using mutect2 and platypus, but was implemented using NextFlow (https://github.com/chelauk/nf-core-mutectplatypus). The buffy coat WGS was used as a reference and the exome panel target region for mutation calling. All timepoints per patient were used as input for mutect2. The MateOnSameContigOrNoMappedMateReadFilter was disabled in mutect2. Instead of using a custom filter, mutect2 calls were only filtered using GATK4 standard practice. The following additional modifications were made to mutation filtering. The minimum mappability was lowered to 0.88 for InDels, normal sample VAF was required to be zero, exact mutation locations were required to be unique in the cfDNA cohort and all mutations with multiple alternatives were removed. VEP was used to annotate the mutations as previously described. Additional calling was not performed using deepSNV. An additional cfDNA sample timepoint was included in joint analysis for both low pass and mutation calling analysis in FI-057 (TP0) but excluded from further analysis due to lack of evidence of ctDNA.

Computational Histopathology

Data acquisition and pre-processing

Digital Whole Slide Images (WSIs) of diagnostic H&E slides from IMRT were acquired using the Zeiss AxioScan.Z1 slide scanner. Slides from this cohort were scanned at a resolution of 0.11 µm/pixel, or an equivalent of a 40x magnification. WSIs from PROMIS, used for initial training of the cell classifier, were scanned using a Hamamatsu Nanozoomer scanner. Slides from this cohort were scanned at a resolution of 0.22 µm/pixel, or an equivalent of a 40x magnification.

For interoperability with images from other scanners, a number of pre-processing steps are applied to the IMRT slide images. Each slide image is converted to a set of 2000x2000 pixel JPEG tiles, at a 0.44 µm/pixel resolution, spanning the entire image with no overlap. The change in resolution is so that the input images match the resolution of the datasets used to train each deep learning model. All images and sizes referenced from this point onwards will be with respect to this resolution, unless otherwise stated. Tiles are then digitally sharpened with unsharp masking, with radius = 5 and amount = 2.

Stain normalisation

There is significant variation in staining across the IMRT cohort. This perhaps due to differences in the tissue preparation and staining protocols for slides originating from many centres. This variation may reduce the accuracy of the deep learning model if the stain appearance differs noticeably from that of the training dataset. Therefore, as a final pre-processing step, Reinhard colour transfer is applied to the JPEG tiles in order to adjust the colours of the H&E stains to match that of a reference whole slide image. Because Gleason Classification and Cell Detection/Classification were trained with distinct datasets, this step is applied separately for each model. Thus, for each stain normalisation a distinct reference whole slide image is selected, corresponding to a slide from the original training dataset. La*b* colour channel statistics for Reinhard colour transfer are computed using only pixels deemed as tissue by tissue segmentation. In addition, only pixels deemed as tissue will be adjusted during colour transfer.

Tissue segmentation

To improve processing speed, a segmentation algorithm is applied to each tile to separate the tissue section from the background glass. An entropy filter is applied to a grayscale version of the tile, which is thresholded with a fixed value of 3.5. Pixels greater than this value are grouped into connected components. Connected components with less than 500 pixels or a median grayscale intensity greater than 225 (based on a full intensity range of 0-255) are removed. Pixels in the remaining connected components form the final segmentation for the tile.

The rationale behind this method is that, unlike tissue, empty glass is predominantly textureless and thus is expected to have a very low value on an entropy filter. Additional checks for size and median intensity are included to remove small artefacts outside of the tissue. However larger artefacts, such as pen markings, may still be partially segmented as tissue using this method.

Tiles that are found to contain no tissue are not processed by the cell detection or Gleason gland segmentation models. In addition, the tissue segmentation is used to filter out results, such as detected cells or segmented glandular regions, that occur in regions with no segmented tissue.

Gleason segmentation

The automated Gleason gland segmentation model takes, as input, two tissue images of size 500x500 pixels: an image at 20x magnification (0.44 microns/pixel) and another at a 10x magnification (0.88 microns/pixel). The two images are centred on the same position on the slide, and thus the 10x image can be considered a zoomed-out view of the region. Due to the use of pre-trained ResNet layers in the model, these images are first resized to 224x224 pixels before being processed by the model.

The output of the network is a segmentation map of the 20x magnification input image, showing the glandular grading of the tissue at each pixel. The model is capable of labelling the tissue with one of

6 labels: No Gland, Normal, Prostatic Intraepithelial Neoplasia (PIN), Gleason 3, Gleason 4, or Gleason 5. However, for this work, Normal and PIN labels were subsequently merged into a single "Benign" class. The raw output of the network is therefore a 224x224x6 array, corresponding to the 6 probability maps for the input 224x224 pixel image patch. To enable direct comparison with the input image patch, probability maps are first resized to the original 500x500 pixel size of the patch.

To scale up segmentation to a whole slide level, analysis is performed in a sliding window fashion. At 20x magnification, the image is divided into 500x500 pixel windows, with 250 pixels of overlap between them. The overlap is included to reduce the presence of border artefacts and improve overall segmentation accuracy. From the output probability maps of these patches, a set of 6 whole slide probability maps are synthesised by joining them together and taking the mean probability for overlapping regions. To avoid potential issues with border artefacts, a border of 10 pixels is discarded from each output probability map before integration into the whole slide probability map.

Upon completion of the whole slide analysis, a segmentation map is generated from the whole slide probability maps. At each pixel, the label with the highest probability is selected. Pixels outside of the tissue segmentation are automatically labelled "No Gland". The resultant segmentation map is used in this work to determine the primary and secondary patterns of the slide for the traditional Gleason score, and for the computation of Gleason Morisita Index.

Gleason model description

The model is based upon a UNet architecture, with modifications (Extended Data Figure 5C). Convolutional layers on the downsampling half of the network are replaced by ResNet blocks, pre-trained on ImageNet data. Convolutional layers remain in place for the upsampling half of the network. All layers were subsequently trained for the specific problem of Gleason segmentation, regardless of whether they were pre-trained.

In addition, modifications have been made to accommodate multi-resolution input. For a given input patch, the N resolution level images are processed in parallel by N sets of downsampling layers. Each set of layers is architecturally identical, but will have its own distinct trainable parameters. The parallel outputs of these downsampling levels are concatenated and then passed through a 1x1 convolutional kernel, before being passed to their corresponding upsampling layer. Unlike the downsampling half of the network, the upsampling half is only composed of a single set of layers.

For our analysis $N=2$ was chosen for the network. This was based upon the observation that an improvement in overall performance could be attained by the addition of a 10x view of the tissue region. This view is perhaps able to provide context of the surrounding area, at the expense of resolution. The inclusion of additional lower resolution levels did not provide any measurable improvement in the accuracy of the segmentation and thus were not used in the final model.

Cell detection

The cell detector uses an SCCNN architecture. The input to the network is a 31x31 pixel image patch of the tissue. The output of the network is an 11x11 pixel probability map with peaks at locations believed to be cell nuclei. A whole slide probability map is generated by applying the network to patches of the slide in a sliding window fashion. The whole slide probability map is converted to a set of detected cell coordinates by identifying the locations of peaks in the map with a maximum clique algorithm. These detected cell positions will generally correspond to the centre of the cell's nucleus. Cells detected in regions outside of the tissue segmentation are filtered out, and the remaining cells are provided as input to the cell classification.

Cell classification

The cell classifier uses a DenseNet-201 architecture. Convolutional layers in the network were pre-trained on ImageNet data, however all layers were subsequently trained for cell classification. The input to the network is a 51x51 pixel image patch at a 20x magnification (0.44 microns/pixel), centred at the coordinate of the detection. Due to the use of pre-trained DenseNet layers, the image patches are resized to 224x224 pixels to fit the required input size. The output to the network is a label for the image patch, identifying the cell type within the image. The model is capable of labelling cells with one of 5 labels: Epithelial cell, Stromal cell, Chronic Inflammatory cell, Acute Inflammatory cell, and Unknown. For this work, Chronic Inflammatory cell and Acute Inflammatory cell labels are merged into single "Immune" cell label. The raw output to the network is a set of 5 probabilities, corresponding to the 5 possible labels. The label with the maximum probability is chosen as the final label.

Gleason Morisita index

Gleason Morsita Index is a quantification of the degree of heterogeneity of the Gleason patterns present within a section. To achieve this, cells identified as epithelial cells by the cell classifier are sub-classified by the Gleason grade of their associated gland. This was achieved by projecting the positions of the cells onto the automated Gleason segmentation and relabelling every cell according to the assessed Gleason pattern at its position (Extended Data Figure 5G).

Morisita Index is a pairwise metric, and thus Gleason Morisita Index is computed between the epithelial cells belonging to the primary and secondary patterns of the slide, as assessed by the automated classifier. Polygons for the Morisita index are generated using Voronoi tessellation, as described in [26]. If the primary and secondary patterns are the same (for example: 4+4) then the Gleason pattern is consider to be completely homogeneous for the section and thus the Gleason Morisita is set to 0. If the Morisita algorithm is unable to compute a Morisita Index for the slide, NaN is returned. At the patient level, the Gleason Morisita index is computed as the median value across all slides from the patient that were determined to be cancer by the automated classifier, with NaNs excluded.

To evaluate the robustness of Gleason Morisita to different methods of computing it, we also propose two alternatives. First, computing the Morisita index directly on the Gleason segmentation maps, rather than on the sub-classified epithelial cells. Secondly, using a 50x50 grid of rectangular regions, rather than a set of Voronoi regions. Both alternative metrics are seen to be well correlated with the chosen version of the metric, and also produced similar predictions for time to recurrence (Extended Data Figure 7).

Gleason scoring from a segmentation map

For comparison with pathologists' assessment, the segmentation map produced by image analysis must be converted into a traditional primary + secondary pattern score. In a broad sense, the computed primary and secondary patterns for a slide correspond to the most frequent and second most frequent pattern in the corresponding segmentation map. However, small amounts of misclassification may interfere with the scoring if this approach is applied naively. In particular, single pattern scores, such as 4+4, would be very unlikely to be selected, as a single pixel of another pattern would be enough to add a second pattern to the map.

Thus, the following criteria is applied to the segmentation map to select the final primary and secondary patterns:

1. Partition segmentation map into **segmented regions**: connected components corresponding to the same Gleason pattern.
2. Discard all segmented regions of size <15 000 pixels (~2 904 sq microns).
3. Sum the remaining area for each grade pattern.
4. Discard grades with combined area <200 000 pixels (~38 720 sq microns) or <10% of total tumour area.

Following this criteria, the remaining grades with the largest and second largest area are selected as the slide's primary and secondary pattern. If only one grade remains, it is selected as both the primary and secondary pattern. If no grade remains, the section is considered benign and is graded as 0+0.

From the Gleason score, a grade group for the slide is determined by applying the ISUP 2014 criteria. A patient-level grade group is computed by taking a weighted mean of the individual slide grade groups and rounding down. Slides are weighted by the size of the tumour area when computing the mean.
