# Supplementary material for: Tumor evolution metrics predict recurrence beyond 10 years in locally advanced prostate cancer
Source: Nat Cancer. 2024 Jul 12;5(9):1334–51. doi: 10.1038/s43018-024-00787-0 (PMC11424488; doi:10.1038/s43018-024-00787-0)
Supplement: Supplementary file 2 — Reporting Summary [file 43018_2024_787_MOESM2_ESM.pdf]

Corresponding author(s): David Dearnaley, Andrea Sottoriva

Last updated by author(s): Mar 21, 2023

Reporting Summary

Nature Portfolio wishes to improve the reproducibility of the work that we publish. This form provides structure for consistency and transparency in reporting. For further information on Nature Portfolio policies, see our Editorial Policies and the Editorial Policy Checklist.

Statistics

For all statistical analyses, confirm that the following items are present in the figure legend, table legend, main text, or Methods section.

n/a	Confirmed	
☐	☒	The exact sample size (*n*) for each experimental group/condition, given as a discrete number and unit of measurement
☐	☒	A statement on whether measurements were taken from distinct samples or whether the same sample was measured repeatedly
☐	☒	The statistical test(s) used AND whether they are one- or two-sided<br>*Only common tests should be described solely by name; describe more complex techniques in the Methods section.*
☐	☒	A description of all covariates tested
☐	☒	A description of any assumptions or corrections, such as tests of normality and adjustment for multiple comparisons
☐	☒	A full description of the statistical parameters including central tendency (e.g. means) or other basic estimates (e.g. regression coefficient) AND variation (e.g. standard deviation) or associated estimates of uncertainty (e.g. confidence intervals)
☐	☒	For null hypothesis testing, the test statistic (e.g. *F*, *t*, *r*) with confidence intervals, effect sizes, degrees of freedom and *P* value noted<br>*Give P values as exact values whenever suitable.*
☒	☐	For Bayesian analysis, information on the choice of priors and Markov chain Monte Carlo settings
☒	☐	For hierarchical and complex designs, identification of the appropriate level for tests and full reporting of outcomes
☐	☒	Estimates of effect sizes (e.g. Cohen's *d*, Pearson's *r*), indicating how they were calculated

Our web collection on statistics for biologists contains articles on many of the points above.

Software and code

Policy information about availability of computer code

Data collection	As per Material and Methods. Sample collection was aided by FreezerPro v7.2. H&E slides for computational pathology analysis were imaged with the Zeiss AxioScan.Z1 slide scanner. Highly multiplexed immunofluorescence images and matched H&E images were acquired using the AKOYA Phenocycler-Fusion scanner.
Data analysis	All statistical analysis related to the genomics was performed in R. The R package survival was used to perform the outcome analysis and the package survminer was used to generate forest plots. Code for image processing and statistical analysis are available as a GitHub repository list: https://github.com/stars/ntrahearn/lists/forecast.<br><br>Software/package versions: R 3.6.1; skewer 0.1.126; bwa 0.7.15; Picard 2.8.1; samtools 1.6.0; QDNAseq 1.22.0; GEM library pre-release 3; MEDICC2 0.4.2; phytools 0.6-99; copynumber 1.24.0; bioMart 2.40.5; fgbio 1.1.0; GATK 4.0.3.0; mapDamage 2.0.8; FASTQC 0.11.9; Qualimap 2.2.2a; vcftools 0.1.15; Platypus 0.8.1.1; VEP 90.1; deepSNV 1.30.0; bigWigAverageOverBed 377; dNdScv 0.0.1.0; survival 2.44-1.1; survminer 0.4.9; lmerTest 3.1-0; ComplexHeatmaps 2.0.0.

For manuscripts utilizing custom algorithms or software that are central to the research but not yet described in published literature, software must be made available to editors and reviewers. We strongly encourage code deposition in a community repository (e.g. GitHub). See the Nature Portfolio guidelines for submitting code & software for further information.

Data

Policy information about availability of data

All manuscripts must include a data availability statement. This statement should provide the following information, where applicable:

- Accession codes, unique identifiers, or web links for publicly available datasets
- A description of any restrictions on data availability
- For clinical datasets or third party data, please ensure that the statement adheres to our policy

Additional analysed data are available on Mendeley: https://data.mendeley.com/datasets/cd9cf2fb76. Sequence data have been deposited at the European Genome-phenome Archive (EGA), which is hosted by the EBI and the CRG, under accession numbers EGAS00001006096 (tumour data) and EGAS00001006098 (normal data).

Human Reference Genome: GRCh38 (full analysis set plus decoy HLA)
1000 Genomes Project: ftp.1000genomes.ebi.ac.uk
dbSNP version 146: dbsnp_146.hg38.vcf.gz

Human research participants

Policy information about studies involving human research participants and Sex and Gender in Research.

Reporting on sex and gender	Patients were recruited as part of the IMRT trial (NCT00946543) or DELINEATE trial (ISRCTN04483921). Sex of all patients was male (gender information was not collected at the time of recruitment to the trials). As all patients were of male sex no sex-based analysis was performed.
Population characteristics	All patients had been diagnosed with a high or very high risk prostate cancer according to NCCN (National Comprehensive Cancer Network) guidelines. The median age of patients was 65 (IQR 60-70).
Recruitment	IMRT trial patients were recruited as outlined as per Ferreira et al., 2017 (https://pubmed.ncbi.nlm.nih.gov/28939224/). Patients were recruited sequentially into the different radiotherapy dose cohorts. DELINEATE trial patients were recruited as outlined as per Murray et al., 2020 (https://pubmed.ncbi.nlm.nih.gov/31812718/). Informed consent was obtained for all patients and no patients were compensated for participation in the study.
Ethics oversight	The (FORECAST) study protocol was approved by the West of Scotland Research Ethics Service in December 2017 (HRA ID 230542)

Note that full information on the approval of the study protocol must also be provided in the manuscript.

Field-specific reporting

Please select the one below that is the best fit for your research. If you are not sure, read the appropriate sections before making your selection.

☒ Life sciences ☐ Behavioural & social sciences ☐ Ecological, evolutionary & environmental sciences

For a reference copy of the document with all sections, see nature.com/documents/nr-reporting-summary-flat.pdf

Life sciences study design

All studies must disclose on these points even when the disclosure is negative.

Sample size	Sample size of this study was determined by the sample size of the IMRT trial (NCT00946543), from which the majority of the data in this work originates. The maximal available subset of samples from the original trial, meeting the necessary criteria for pathology and sequencing, were used in this study. The criteria used to determine this subset are explained in the submitted manuscript.
Data exclusions	Specific exclusions, such as samples without copy number alterations, are indicated in text for certain analyses. General quality control exclusion criteria are also listed in the methods section for genomic data, i.e. low coverage in targeted sequencing data. One cfDNA sample was excluded due lack of detectable tumour DNA and is referred to in text and in the Methods section. One low pass whole genome sequencing sample was excluded from analysis due to having no copy number alterations and being classified as benign. These samples are still included in the data repository.
Replication	Not Applicable
Randomization	IMRT: as per Ferreira et al. 2017; DELINEATE: as per Murray et al. 2020.
Blinding	Those involved in sample preparation and data analysis were blinded until the completion of the primary phase of data analysis on diagnostic biopsies. As a result, the selection of samples for sequencing and imaging, and selection of genomic and histological features for analysis were finalised prior to unblinding. Review pathology, including Gleason grade, was also undertaken blinded to the original pathology and clinical

data. To further explore associations that were identified in the primary phase, a secondary phase of data analysis was conducted after unblinding, which was focused exclusively on newly generated cfDNA and multiplex immunohistochemistry data.

Reporting for specific materials, systems and methods

We require information from authors about some types of materials, experimental systems and methods used in many studies. Here, indicate whether each material, system or method listed is relevant to your study. If you are not sure if a list item applies to your research, read the appropriate section before selecting a response.

Materials & experimental systems

n/a	Involved in the study	
☐	☒	Antibodies
☒	☐	Eukaryotic cell lines
☒	☐	Palaeontology and archaeology
☒	☐	Animals and other organisms
☒	☐	Clinical data
☒	☐	Dual use research of concern

Methods

n/a	Involved in the study	
☒	☐	ChIP-seq
☒	☐	Flow cytometry
☒	☐	MRI-based neuroimaging

Antibodies

Antibodies used

Marker: CD3e, Clone: EP449E, Catalogue Number: 240006, Lot Number: B358626, Supplier: AKOYA Biosciences, Dilution: 1:200
Marker: CD8, Clone: C8/144B, Catalogue Number: 232151, Lot Number: B355600, Supplier: AKOYA Biosciences, Dilution: 1:200
Marker: CD20, Clone: L26, Catalogue Number: 232175, Lot Number:B356690, Supplier: AKOYA Biosciences, Dilution: 1:200
Marker: CD4, Clone: EPR6855, Catalogue Number: 232170, Lot Number:B360688, Supplier: AKOYA Biosciences, Dilution: 2:200
Marker: CD31, Clone: EP3095, Catalogue Number: 232172, Lot Number: B360631, Supplier: AKOYA Biosciences, Dilution: 1:200
Marker: Ki67, Clone: B56, Catalogue Number: 232179, Lot Number:B350899, Supplier: AKOYA Biosciences, Dilution: 1:200
Marker: PCK, Clone: AE-1/AE-3, Catalogue Number: 232180, Lot Number: B367234, Supplier: AKOYA Biosciences, Dilution: 2:200
Marker: TP63, Clone: AKYP0111, Catalogue Number: 240179, Lot Number:B363038, Supplier: AKOYA Biosciences, Dilution: 1:200
Marker: CK18, Clone: EPR1626, Catalogue Number: ab240054, Lot Number: GR3365669-2, Supplier: Abcam, Dilution: 1:200
Marker: FSP1, Clone: EPR2761(2), Catalogue Number: ab216003, Lot Number: GR317174-7, Supplier: Abcam, Dilution: 1:200
Marker: CD163, Clone: EDHu-1, Catalogue Number: NB110-40686, Lot Number: 149022B, Supplier: Novus Biologicals, Dilution: 1:200
Marker: αSMA, Clone: Polyclonal, Catalogue Number: ab5694, Lot Number: GR3356867-4, Supplier: Abcam, Dilution: 1:200
Marker: Vimentin, Clone: RV202, Catalogue Number: 550513, Lot Number: 6316850, Supplier: BDBiosciences, Dilution: 1:200

Validation

CD3e, CD8, CD20, CD4, CD31, Ki-67, PCK and TP63 were purchased as ready to use, conjugated and validated antibodies from AKOYA (https://www.akoyabio.com/phenocycler/assays/). Details of AKOYA validation can be found at the following link: https://www.akoyabio.com/wp-content/uploads/2022/01/Phenocycler_Technical-Note_Validation-of-Commercial_DN-00140.pdf

CK18, FSP1, CD163, αSMA and Vimentin were purchased as purified antibodies, which required conjugation for use in the PhenoCycler Fusion platform.

For conjugated antibodies, validation process was as follows:
- To ensure the antibody is working under the required conditions and to determine an ideal concentration for each antibody, a standard Immunohistochemistry (IHC) assay was run on marker specific positive and negative control tissues.
- Following a successful IHC assay, the antibodies were conjugated to their assigned barcode. An electrophoresis gel was run to validate the conjugation.
- To ensure that each of the conjugated antibodies could be imaged adequately by the PhenoCycler Fusion, test runs of the individual markers and the complete multiplex IHC panel were performed on positive control tissue sections.
